# Lethal plague outbreaks in Lake Baikal hunter-gatherers 5,500 years ago

**DOI:** 10.1038/s41586-026-10540-5

**Published:** 2026-06-17

**Authors:** Ruairidh Macleod, Frederik V. Seersholm, Bianca De Sanctis, Angela Lieverse, Adrian Timpson, Rick Schulting, Jesper T. Stenderup, Charleen Gaunitz, Lasse Vinner, Olga Ivanovna Goriunova, Vladimir Ivanovich Bazaliiskii, Sergei V. Vasilyev, Erin Jessup, Yucheng Wang, Christopher Bronk Ramsey, Mark G. Thomas, Russell Corbett-Detig, Astrid K. N. Iversen, Andrzej W. Weber, Martin Sikora, Eske Willerslev

**Affiliations:** 1 Lundbeck Foundation Geogenetics Centre, Globe Institute, University of Copenhagen, Copenhagen, Denmark; 2 All Souls College, University of Oxford, Oxford, UK; 3 Centre for Ancient Environmental Genomics, Globe Institute, University of Copenhagen, Copenhagen, Denmark; 4 Department of Genetics, University of Cambridge, Cambridge, UK; 5 Department of Biomolecular Engineering, UC Santa Cruz, Santa Cruz, CA USA; 6 Genomics Institute, UC Santa Cruz, Santa Cruz, CA USA; 7 Department of Anthropology, University of Saskatchewan, Saskatoon, Saskatchewan Canada; 8 Research Department of Genetics, Evolution and Environment, University College London, London, UK; 9 School of Archaeology, University of Oxford, Oxford, UK; 10 Scientific Research Centre “Baikalskii Region”, Irkutsk State University, Irkutsk, Russia; 11 Institute of Ethnology and Anthropology, Russian Academy of Sciences, Moscow, Russia; 12 Department of Anthropology, University of Alberta, Edmonton, Alberta Canada; 13 Group of Alpine Paleoecology and Human Adaptation (ALPHA), State Key Laboratory of Tibetan Plateau Earth System, Environment and Resources (TPESER), Institute of Tibetan Plateau Research, Chinese Academy of Sciences, Beijing, China; 14 Nuffield Department of Clinical Neurosciences, John Radcliffe Hospital, Weatherall Institute of Molecular Medicine, University of Oxford, Oxford, UK

**Keywords:** Evolutionary biology, Archaeology, Infectious diseases

## Abstract

Plague is among the most devastating diseases in human history^1^. However, early strains of the plague-causing bacterium *Yersinia pestis* lacked virulence factors that are required for the bubonic form until around 3,800 years ago^2,3^. Consequently, the morbidity and mortality of early plague strains remain unclear. Here we describe early plague strains that are associated with two phases of outbreaks among mid-Holocene hunter-gatherers near Lake Baikal in southeast Siberia, beginning from about 5,500 years ago. These outbreaks occur across four hunter-gatherer cemeteries, with a 39% detection rate for plague infection. By reconstructing kinship pedigrees, we show that small familial groups were affected, consistent with human-to-human spread of disease, and that the first outbreak occurred within a single generation. The infections appear to have resulted in acute mortality, especially among children (aged 8 to 11 years). We further note functional differences, including in the *ypm* superantigen locus, which is also present in present day *Yersinia pseudotuberculosis*. The new strains diverge ancestrally to known *Y. pestis* and constrain the timing of its emergence, indicating that this happened before approximately 5,700 years ago. These findings show that plague outbreaks happened earlier than previously thought and were indeed lethal. We contend that the occurrence of outbreaks among mid-Holocene hunter-gatherer communities well outside the sphere of Late Neolithic Europe challenges the notion that higher population densities and lifestyle changes during the Neolithic agricultural transition were prerequisites for plague epidemics.

## Abstract (web summary)

Analyses of ancient DNA from hunter-gatherers near Lake Baikal in southeast Siberia around 5,500 years ago indicate that highly virulent *Yersinia pestis* emerged earlier than previously estimated, far from the next known cases of infection in Late Neolithic Europe.

## Main

The analysis of ancient pathogen genomes has significantly expanded our understanding of the evolutionary history of human infectious diseases (for example, *Salmonella enterica*^4^ and hepatitis B^5^), although this has principally been in the context of farming or pastoralist communities. *Y. pestis*, the aetiological agent of plague, is perhaps the most studied in this regard, and has had devastating consequences on human populations for millennia. Historical outbreaks of plague account for some of the most fatal events in human history^1^. The recovery of ancient DNA from plague victims has afforded extraordinary insights into the origins and evolution of plague at the time of these events^6,7^, and, remarkably, revealed infections in prehistoric individuals across Europe^8^. Historically and today, plague is associated with transmission via fleas from rodents, which successfully adapted to a human commensal niche in the Neolithic^9^. Genomic analysis of prehistoric plague indicates that in early diverging strains, key genetic adaptations required for flea-mediated transmission of the disease and bubonic infection are absent^2,3^, leading to uncertainty over the transmission route and severity of these strains.

The detection of early plague cases across multiple generations of Late Neolithic farmers has been used to link outbreaks of the disease to a prolonged demographic decline between about 5,300–4,900 calibrated years before the present (cal bp)^10,11^, although an alternative explanation attributes the decline to agricultural crisis^12,13^. The former interpretation has been controversial, with others suggesting infections as more closely resembling benign foodborne enteritis^14^. The similarity or otherwise of these early strains to *Y. pseudotuberculosis*—the closest relative of * Y. pestis*—has been an important point of interest through such discussions, and based on existing ancient genomes, *Y. pestis* has been estimated to have diverged from *Y. pseudotuberculosis* some time in the past 50,000 years (refs. ^8,11,15^).

Studies of prehistoric plague genomes from Late Neolithic and Bronze Age (LNBA) strains predominantly date to between 4700–2400 cal bp (refs. ^3,8,16^), and are typically defined as one of two lineages, depending on the presence (LNBA+) or absence (LNBA−) of the *ymt* gene^3^. *ymt* encodes *Yersinia* murine toxin, which enhances bacterial survival in the flea digestive tract during the transition period between rodent and human hosts, and thereby the flea bite-transmitted bubonic form of plague in humans^17^. Lineages of *Y. pestis* that diverged prior to these LNBA clades have also been identified in a handful of Neolithic Swedish individuals (5200-4850 cal bp)^10,11^ and a Latvian individual with western hunter-gatherer ancestry (5300–5050 cal bp)^15^. These genomes lack classic virulence genes (*YpfΦ* prophage and *ymt*), although pangenomic analysis revealed the presence of the locus encoding for *Y. pseudotuberculosis*-derived mitogen (YPM), a superantigenic toxin associated with *Y. pseudotuberculosis* (but not later *Y. pestis* strains). This raises intriguing questions about the possible severity of early strains of plague; subsequent LNBA− strains show substantial gene loss, although the virulence potential of these are unknown^3^. Evidence regarding the demographic impact of plague infection on prehistoric populations has so far been lacking in these studies.

**Fig. 1:**
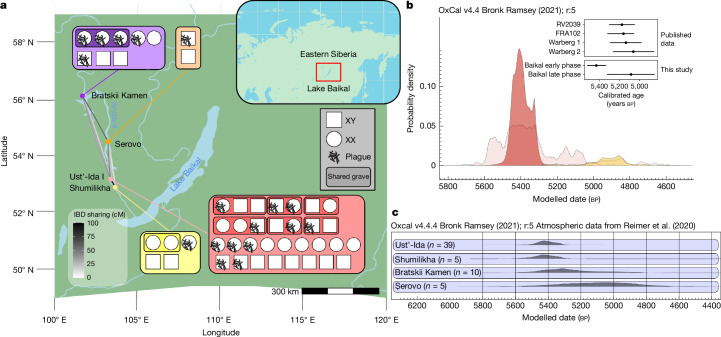
Overview of the spatiotemporal distribution of ancient humans and plague infections in this study. **a**, Locations of affected cemeteries on the Angara River northwest of Lake Baikal, and IBD sharing between the sampled occupants of cemeteries (pairwise sharing lines between sites; greyscale ramp indicates total IBD sharing in segments larger than 3 centimorgans (cM) totalling more than 10 cM in pairwise relationships between sites; Supplementary Note 2). Inset represent the sampled individuals at each site (31 Ust’-Ida I, 8 Bratskii Kamen, 2 Serovo and 5 Shumilkha), with plague detections indicated, and shared graves indicated by shaded areas around individuals. Maps created using Natural Earth Data. **b**, Kernel density estimates plotted within Bayesian models of the date ranges for the early (red) and late (dark yellow) phases of plague outbreaks at Baikal. Lighter shaded areas correspond to the summed probability distributions prior to modelling; dates used are from individuals identified with plague only. Inset, 95.4% confidence intervals for modelled date ranges at Baikal are shown compared with those from other pre-LNBA plague cases: RV2039 from Latvia^15^, Warberg 1 and Warberg 2 from Germany^61^ and FRA102 from Sweden^11^. **c**, Kernel density estimate modelled radiocarbon date distributions for the four cemetery sites, for all radiocarbon-dated post-weaning age humans (or associated deer tooth pendants), irrespective of DNA sampling. Radiocarbon date modelling undertaken with OxCal v.4.4.4^65,66^.

**Extended Data Fig. 1:**
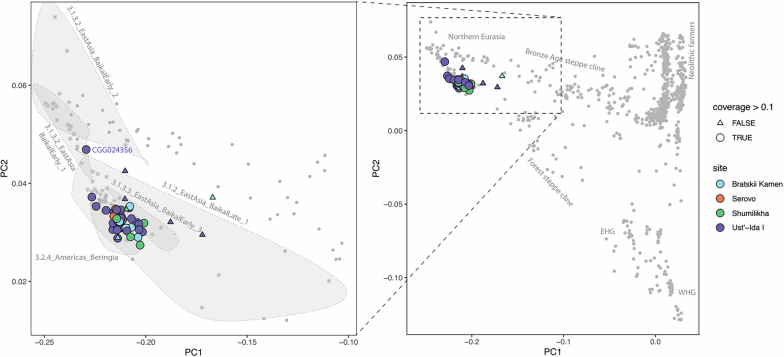
Principal Components Analysis (PCA) of ancient human genomes from Baikal hunter–gatherers studied here. Ancient genomes for 46 ancient individuals (31 Ust’-Ida I; 8 Bratskii Kamen; 2 Serovo; and 5 Shumilkha) are projected onto a background PCA using as a reference the dataset from Allentoft et al.^68^. Reference data is coloured grey, while test samples are colored by sample site. Low coverage samples are represented by triangles. On the left hand insert plot, the five populations with the highest similarity to our test samples have been highlighted. Further genetic ancestry analyses, including F-statistic tests, are described in Supplementary Note 2.

**Extended Data Fig. 2:**
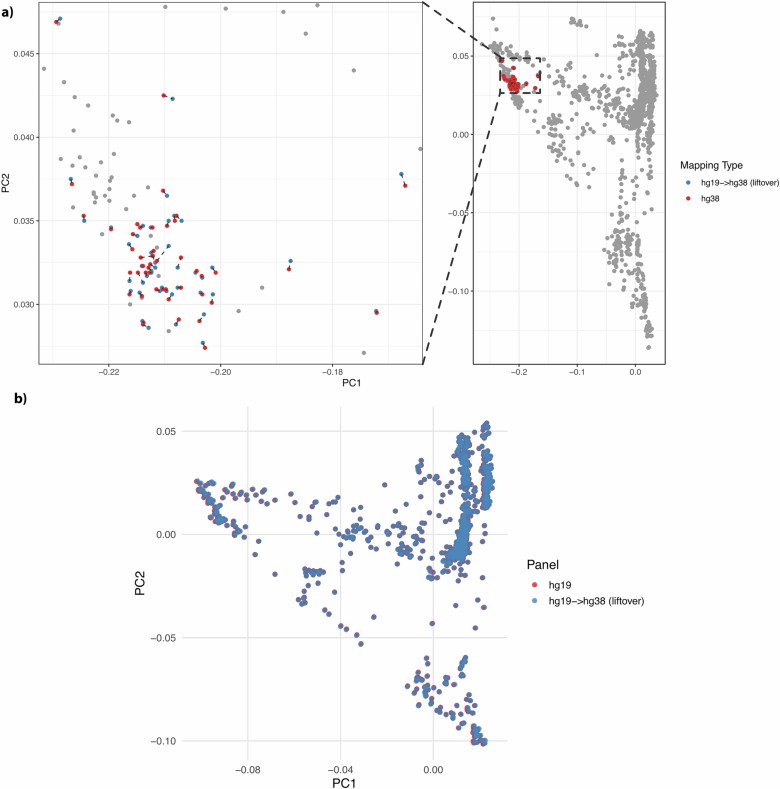
Comparing the effects of mapping to human genome builds hg19 and hg38. a) PCA analysis comparing test samples mapped directly to hg38 (red color) and mapped to hg19 and lifted over to hg38 (blue color), using smartPCA. b) The effect of hg19 to hg38 liftover using smartPCA, shown by procrustes analysis comparing the smart PCA space for our original panel mapped to hg37 and that same panel lifted over to hg38. Further details and results of comparisons (including comparisons of euclidean distances and F statistic tests) are provided in Supplementary Note 6.

Middle Holocene hunter-gatherers around Lake Baikal, southeast Siberia, have been the focus of intensive archaeological study by the Baikal Archaeology Project, yielding important datasets for framing prehistoric hunter-gatherer lifeways^18,19^. These groups demonstrate remarkable continuity of hunter-gatherer lifeways and subsistence, evidenced by an extensive archaeological record of mortuary sites from between about 8500–3500 cal bp (ref. ^20^). The genomes of sampled hunter-gatherers indicate a long-term continuum of Ancient North Eurasian and North East Asian ancestry until c. 4500–4000 cal bp (refs. ^21,22^) (Extended Data Figs. 1 and 2). By this period, cases of plague from human remains corresponding to the LNBA− strain are documented sporadically among Early Bronze Age burials^22,23^. Zoonotic spillover events causing plague infections in this region remain a major health concern to this day^24^. These are principally associated with marmots, the primary zoonotic reservoir of plague in the region^25,26^. To explore health and community structure in prehistoric hunter-gatherer groups, we analysed ancient human and pathogen DNA from four cemetery sites in Cis-Baikal (the lake’s western and northern region) across two separate outbreaks dated to 5520–5265 cal bp and 5315–4235 cal bp (95.4% confidence intervals for modelled date ranges based on individuals with detected plague cases, corrected for freshwater reservoir effects; Supplementary Note 4). The long tail for the second outbreak date range (Fig. 1b) is due to this only consisting of two direct dates, although the highest likelihood date range for this is approximately 5050–4850 cal bp.

## Outbreaks of basal plague strains

We generated shotgun-sequenced ancient DNA from 46 Late Neolithic individuals and examined this data for presence of pathogens (Methods). This revealed a conspicuously high occurrence of *Y. pestis* among these individuals, more so than any other pathogen. *Y. pestis* was detected in 18 individuals, indicating 2 distinct phases of outbreaks of plague infection—separated by between 4 and 6 centuries—in 4 cemeteries (Fig. 1a,b). These occur across two phases at Shumilikha, Ust’-Ida I, Bratskii Kamen and Serovo (see Fig. 1c), with cases from Bratskii Kamen in both the first and second phases. These sites are all located on banks of the River Angara, a major watercourse draining from Lake Baikal, with a rich fishery^27^. Stable carbon and nitrogen isotopic data from individuals at Ust’-Ida I evidence consumption of both local fish and terrestrial game^27^. Burials at Ust’-Ida I and Shumilikha correspond to the Isakovo mortuary tradition (characterized by bodies that are typically oriented parallel to the river, and the presence of grave goods such as mitre-shaped clay vessels, lithic arrowheads and bone or antler points), whereas those at Bratskii Kamen and Serovo correspond to the Serovo mortuary tradition (with bodies frequently oriented perpendicular to the river; bifaces and egg-shaped pots as grave goods are the main features; see also Supplementary Note 1). At Ust’-Ida I, we also detect reads aligned to the zoonotic pathogen *Brucella*, the cause of brucellosis, in one individual (#26.04; Supplementary Note 3). The two plague outbreaks are grouped by the predominant burial practices at each cemetery: Isakovo-style graves in the first outbreak and Serovo-style graves in the second phase (Supplementary Note 1), which are contemporaneous at Lake Baikal between around 6000–5000 cal bp (ref. ^27^). This period is defined locally as the Late Neolithic, following Siberian archaeological terminology, where the Neolithic is defined on technological criteria such as the introduction of the bow and arrow, clay vessels and stone grinding techniques (domestic plants and animals other than dogs are absent), although these communities remain as hunter-gatherers until the encroachment of pastoralism in the Late Bronze Age. Grave sites comprise the vast majority of the Cis-Baikal archaeological record, and designations such as the Late Neolithic are later categorizations, applied to distinct sets of burial characteristics and grave goods that broadly correspond to different periods. All four cemeteries were also used during the Early Neolithic (7650–6660 cal bp) and Early Bronze Age (4970–3470 cal bp)^27^, although only their Late Neolithic components are considered here.

Pairwise sharing of identity-by-descent (IBD) segments between individuals at these cemeteries indicates recent shared ancestry. Although they are up to 340 km apart, the Angara river would readily have facilitated travel. Very low rates of inbreeding were detected and a high effective population size based on runs of homozygosity was inferred using hapROH (maximum likelihood estimate: 18,219 individuals, 95% confidence interval 9,445–42,062). This is consistent with the scenario of highly mobile, exogamous hunter-gatherer groups.

Within the hunter-gatherer individuals analysed here, the highest number of detected plague infections was at Ust’-Ida I, which is also the largest Isakovo mortuary site in Cis-Baikal. Here, we found a 35% detection rate (11 out of 31 individuals sequenced), including burials #14 and #56.01, for which human genome data were previously reported^21^. Across other sites, we identify one high-coverage plague genome at Shumilikha, four lower coverage genomes from Bratskii Kamen, and one medium coverage genome from Serovo. Overall, we observe a 39% detection rate across Late Neolithic individuals at these cemeteries (from dental cementum). In comparison, quantitative PCR screening of known Mediaeval plague victims at Smithfield, London, UK^28^ returned a detection rate of 5.7% from bone and 37% from dental pulp tissue (overall 20%), indicating a high rate of false negative plague detection using ancient DNA. To prevent misrepresentation of data, all ancient individuals with screening data from the affected sites are reported here (human autosomal genome coverage ranges from 0.001× to 1.9×, average 0.65×). Direct radiocarbon dates were obtained from nearly all the individuals within the Late Neolithic components of these cemeteries (a total of 58, including those previously reported from Ust’-Ida I^29^; Supplementary Data 7).

**Fig. 2:**
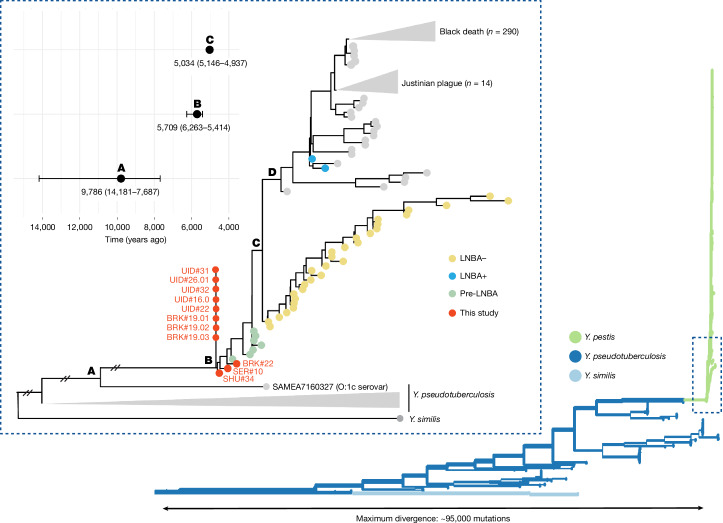
Phylogenetic relationships and inferred internal node dates between hunter-gatherer plague samples from this study and previously published data. Right, the overall topology of the *Y. pseudotuberculosis* species complex is shown from a mutation-annotated tree based on 448 genomes (branch lengths indicate distance by mutations). Inset, a simplified version of this phylogeny, with prehistoric plague strains shown in particular (some branch lengths truncated). The three Baikal samples with higher coverage were incorporated directly into the construction of a RAxML phylogeny, whereas the eight lower coverage samples were phylogenetically placed afterwards, all of which shared their most parsimonious placement at the most basal *Y. pestis* node. Their placement at this node does not constitute a branching position, thus their inclusion adjacent to this node. Top left, internal node dates estimated using BactDating. BRK, Bratskii Kamen; SER, Serovo; SHU, Shumilikha; UID, Ust’-Ida I.

**Extended Data Fig. 3:**
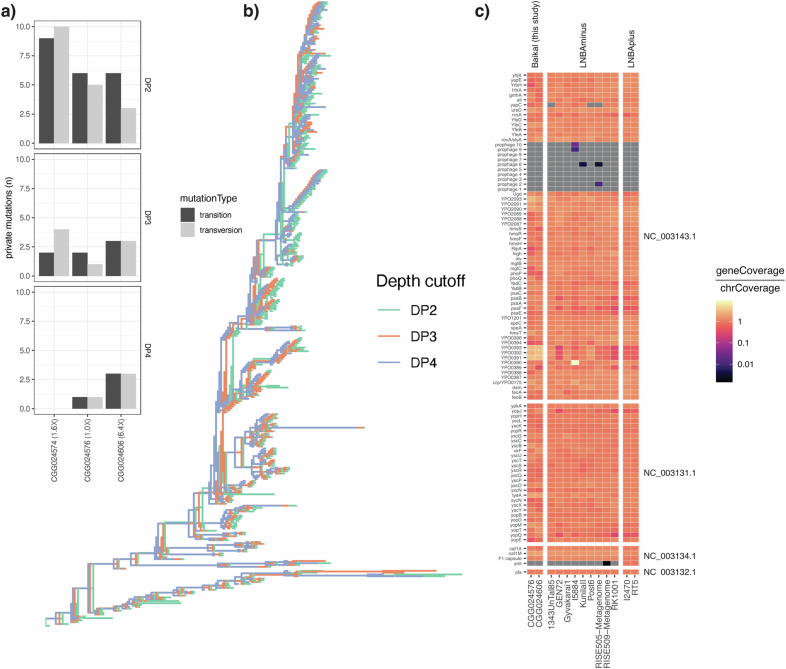
Genome analysis of *Yersinia pestis* sequences. a) Number of private mutations for each higher coverage plague genome using different depth cut offs of variant calling (DP2, DP3 and DP4 corresponding to minimum depth thresholds of 2x, 3x and 4x, respectively) stratified in transitions and transversions. b) Comparison of phylogenetic trees inferred with the same depth threshold values. Trees inferred using different depth thresholds for calling genotypes are overlaid on top of each other. c) Heatmap of coverage for classic virulence-associated genes from *Y. pestis*. Colour gradient corresponds to the average depth of coverage of the specified gene, divided by the average depth of coverage of the entire chromosome. The two highest coverage Baikal samples, Shumilikha #34 (CGG024606) and Serovo #10 (CGG024576) are shown at the bottom alongside previously LNBA- and LNBA+ strains.

*Y. pestis* genomes identified between the two Baikal phases of outbreaks were found to diverge ancestrally to the current known clade of ancient and modern plague strains (Fig. 2). We confidently assign these to *Y. pestis* from their phylogenetic position, and also the presence of virulence genes and plasmids characteristic of *Y. pestis* (Extended Data Fig. 3 and Supplementary Note 3). This phylogeny was built using genomes obtained from Shumilikha Burial #34 (6.4× coverage) from the first phase, and from Bratskii Kamen Burial #22 (1.6×) and Serovo Burial #10 (1.0×) from the second phase. Eight lower coverage genomes were phylogenetically placed using UShER^30^. The UShER algorithm finds the most parsimonious placement on the tree, selecting the node with the greatest number of descendents if multiple are equally parsimonious, and ignores missing genotypes. Placement of all low-coverage genomes at the same basal node is partly due to data missingness, although consistent with the position of the three higher coverage Baikal genomes. Bayesian inference of node dates was undertaken following an approach to account for the effects of recombination within bacterial phylogenies^31,32^ (Methods and Supplementary Note 3). The emergence of *Y. pestis* as a clonal species of *Y. pseudotuberculosis* occurs some time between the divergence of the lineage that gives rise to *Y. pestis* (labelled node A in Fig. 2) and the most recent common ancestor of available *Y. pestis* genomes (node B in Fig. 2). The upper bound provided by the former is likely to be substantially affected by the paucity *Y. pseudotuberculosis* genomes sequences, and could well be more recent if phylogenetically closer serovars were identified. Nonetheless, this lower bound (with a mean date of 5,709 years ago) revises a previous divergence estimate of 4,810–5,122 years ago^33^, as would be expected by including *Y. pestis* genomes older than this range (other estimates of this have ranged from 6,000 to 50,000 years ago^8^ and 7,400 years ago^15^). The phylogeny supports the conclusion that *Y. pestis* first evolved from a variant of the O:1 *Y. pseudotuberculosis* strain (represented by a genome from serotype O:1c, European Nucleotide Archive (ENA) accession: SAMEA7160327), consistent with previous findings^34^ reporting the inactivation of the O-antigen gene cluster as a step towards the evolution of *Y. pestis*. Between the two phases, we observe small genetic differences between strains in distinct private mutations in the first and second phase strains (with strict filters for genotype calling; Methods and Supplementary Note 3); this is also clear from the position of nodes in Fig. 2. Although mutation rates in *Y. pestis* are known to be highly variable within different lineages^33^, this result is consistent with a scenario of related strains resulting from separate zoonotic spillover events from a local animal reservoir.

## Baikal hunter-gatherer plague mortality

**Fig. 3:**
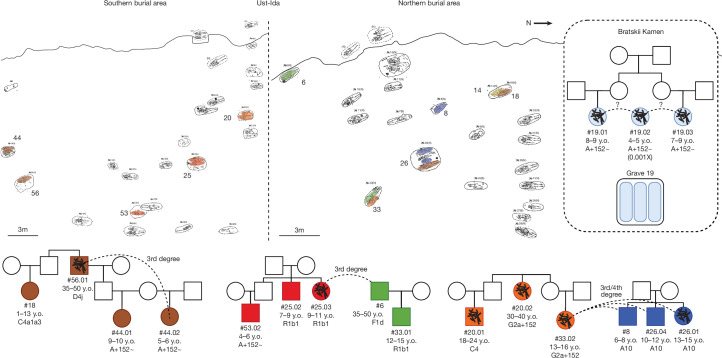
Familial pedigree groups identified from ancient genomes and the site plan for the Ust’-Ida I cemetery. Individuals detected for plague are marked with bacilli silhouettes. Pedigrees are drawn from 30 sampled individuals at Ust’-Ida I and 8 from Bratskii Kamen; only close familial relationships are shown; although many other third- or fourth-degree relationships are detected at Ust’-Ida I (see Supplementary Note 2). Pedigrees are reconstructed based on deaths occurring contemporaneously. The two samples from Serovo (not shown) were found to be fourth-degree relatives. y.o., years old.

**Extended Data Fig. 4:**
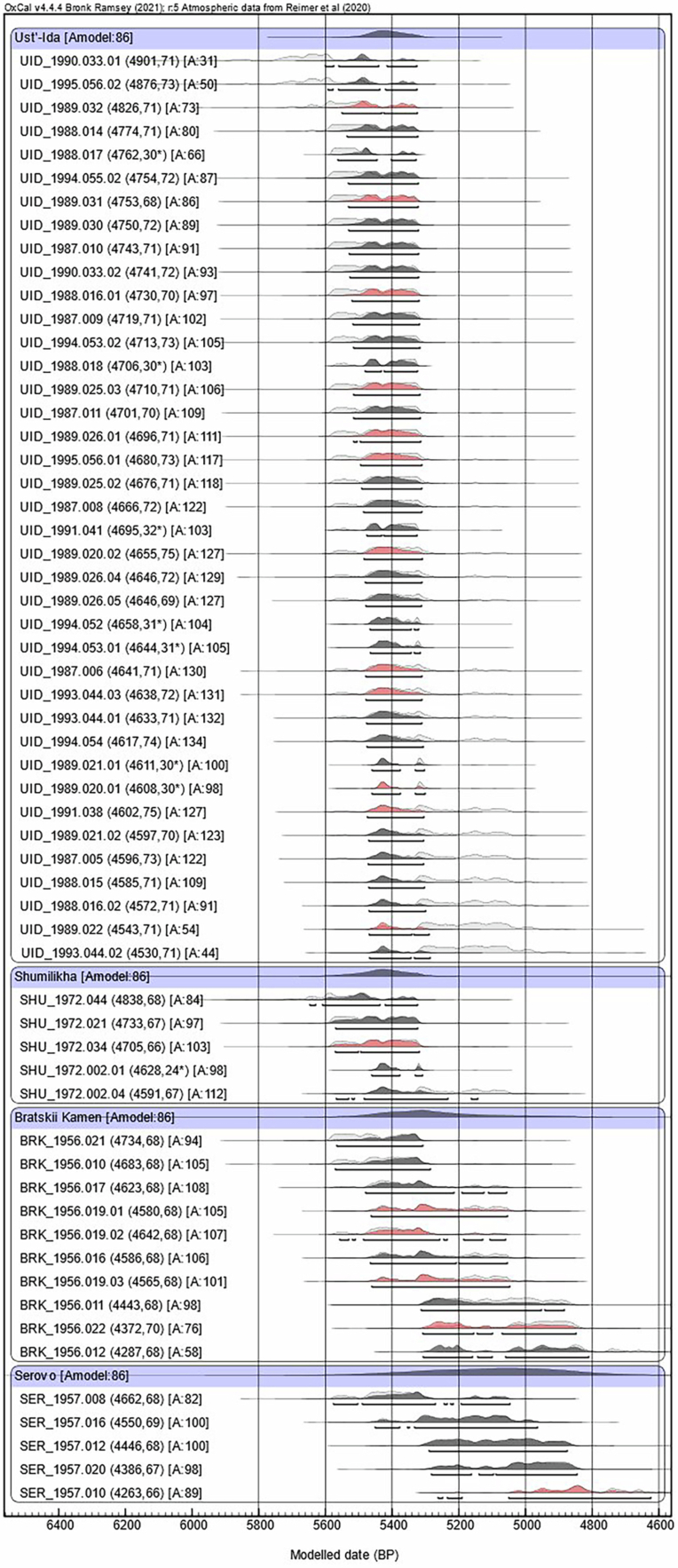
Radiocarbon dating results from remains studied here. Site-based KDE models of all radiocarbon-dated individuals (or associated red deer tooth pendants, marked by asterisks) from the four Late Neolithic cemeteries on the Angara River with plague victims (indicated in red). Radiocarbon dates obtained from human remains are corrected for the freshwater reservoir effect (see Supplementary Note 4).

To contextualize these plague outbreaks, we considered biological kinship patterns, burial treatment and age at death within the affected hunter-gatherer cemeteries. At the site with the highest positive detection of plague (and largest sample), Ust’-Ida I, radiocarbon dates for the Late Neolithic Isakovo component are exceptionally tightly clustered for a relatively large cemetery^29^ (Extended Data Fig. 4). Modelled date ranges for all the early phase plague victims indicate a very narrow temporal span, on the order of a few decades (Supplementary Note 4), supporting the scenario that these burials were contemporaneous. This is further corroborated by the high similarity among plague genomes consistent with plague infections occurring in a single outbreak, or over a very brief time span. By reconstructing the most likely familial pedigrees, we find that the relationships and ages of family members are consistent with a mortality event over a time span of less than a single generation (Fig. 3). None of the age at death–relationship pairings indicate, for example, children that reached a similar age to their parents, or siblings and half-siblings with very different ages (the greatest sibling age gap is nine years, separated by a middle sibling). Where multiple generations are present, their inferred age-at-death ranges are generally consistent with those expected if all relatives had died at the same time (for example, a 12–15 year old has a 35–50-year-old father).

**Fig. 4:**
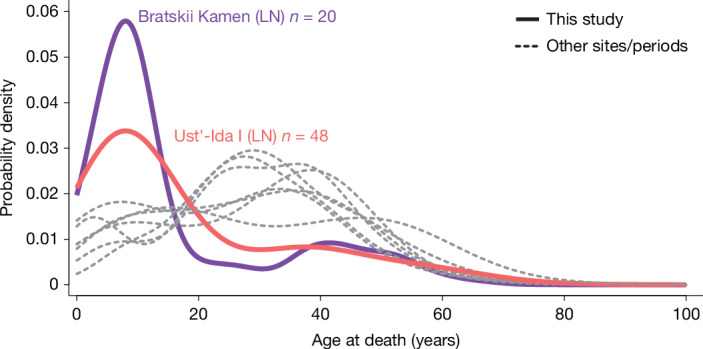
Mortality profiles at Bratskii Kamen and Ust’-Ida I compared with other Baikal hunter-gatherer cemetery populations. Kernel density plot of modelled age-at-death probabilities, based on a null model of a continuous probability of death at any age (see Supplementary Note 5). All relevant assemblages of human skeletal remains from the Cis-Baikal region studied by A.L. with more than 20 individuals are shown. Sample sizes for sites depicted: Bratskii Kamen (Late Neolithic (LN)), *n* = 20; Khuzhir-Nuge XIV, *n* = 81; Lokomotiv, *n* = 101; Shamanka II, *n* = 156; Shumilikha, *n* = 36 (Early Bronze Age); Ust’-Ida I, *n* = 48; Verkholensk, *n* = 27. Individuals are only included from the predominant period of mortuary use at each site. Outlier individuals from later or earlier mortuary traditions are excluded.

The Isakovo mortuary group at Ust’-Ida I is unusual in several other ways among Cis-Baikal hunter-gatherer cemeteries. In addition to the tightly clustered radiocarbon dates, childhood mortality is disproportionately high (also observed at Bratskii Kamen, see Fig. 4), and there is a high incidence of multiple-interment graves (more than half at the site) with no evidence of subsequent grave opening and addition of new burials. This suggests co-occurrence of deaths within shared graves, consistent with a catastrophic mortality event. An avuncular relationship was also detected using KIN^35^ between the Ust’-Ida I and Shumilkha cemeteries, but this was not substantiated by the expected IBD-sharing pattern (Supplementary Note 2). Nonetheless, the high degree of IBD sharing between individuals (Fig. 1) across a distance of only 37 km along the Angara river, suggests that the concurrent plague outbreaks might be linked to the groups being in close contact at this time point.

In terms of plague detection within grave groups, we find no statistically significant pattern of plague co-occurrence among relatives (Supplementary Note 3), although affected individuals appear to be associated in a number of cases. The burial at Bratskii Kamen features a shared grave of 3 young girls, aged between 4 and 9 years (Fig. 3, left), with similar radiocarbon dates (Supplementary Note 4). Two of them (#19.01 and #19.03) were inferred as third-degree related (most probably cousins); the third had insufficient DNA preservation to confidently infer relatedness, but all three shared a mitochondrial haplotype with three rare private mutations and so were likely to be close maternal relatives. Genome data for *Y. pestis* were identified in all three, suggesting an outbreak of plague infection in a family, with synchronous deaths of the three children. Similarly, at Ust’-Ida I, a nephew and aunt (#20.01 and #20.02) are buried in a shared grave, with *Y. pestis* identified from both (Fig. 3, orange pedigree). The teenage niece of the aunt, however, is buried in a different shared grave with an unrelated teenage male (possibly suggesting non-biological kinship); his father in turn (green pedigree) is buried in an entirely separate grave.

Additionally, some pairs of siblings who are buried together in shared graves show only one individual detected as positive for plague, as is the case with the siblings in grave #25 (Fig. 3, red pedigree). In another example, for a sister (#26.01) and brother (#26.04), the sister is inferred as positive, whereas the brother is not (although *Y. pestis* reads are detected at just below the threshold for confident identification; Supplementary Data). These observations are consistent with a high false negative detection rate in the palaeogenomic analysis of plague^28^. The brother was also infected with probably non-lethal brucellosis (Supplementary Note 3). In several cases close family members are found in different graves within the cemetery, for example, Burial #8, the third sibling of the pair in Grave 26. A pattern is visible, where two closely related family members are buried together, and one or more others are buried further away. This may be consistent with a more drawn-out sequence of deaths instead of a single mortality event if shared graves indicate concurrent deaths, reflecting a scenario of delayed person-to-person disease transmission. No causes of death were apparent other than genetically detected plague infection (although other microbes detected might reflect bacterial coinfections at the time of death; Supplementary Note 3). Notably, survivors must have existed to bury the deceased, with the typical Isakovo mortuary treatment and grave goods as well as acknowledgement of biological kinship suggesting a more prolonged sequence of mortality events.

## Epidemiological implications

At Baikal, the principal contemporary zoonotic reservoir of plague is the marmot (*Marmota sibirica*), and marmot hunting for meat and fur has historically resulted in perennial plague infections especially in young men, who are exposed during skinning and butchery^36^. Since the nineteenth century, marmots were the most targeted game species by Indigenous hunters in this region, originally by trapping^37^, and there are extensive historical accounts of ‘tarbagan plague’ from consumption of infected marmots around Lake Baikal^38^. Prehistoric hunter-gatherer marmot procurement is clearly evidenced by the presence of numerous marmot teeth as grave goods in Early Neolithic Kitoi graves^19,39^, although these have not been found in Late Neolithic graves. Consumption of raw or undercooked marmot organs results in the septicaemic form of infection following the faecal–oral transmission route, whereas close contact with marmots infected by present-day *Y. pestis* strains causes bubonic or pneumonic plague (or often both), with the latter often occurring secondarily to septicaemic infection^40^ or inhalation of infectious blood droplets during, for example, skinning^41^. The incidence of detected infections among co-buried kin described above would be consistent with the transmission of plague among humans, particularly via pneumonic transmission in the scenario of concurrent deaths.

A striking aspect of the osteological age-at-death data at Ust’-Ida I and Bratskii Kamen—the two cemeteries with multiple instances of plague detected—is that their demographic profiles are highly skewed towards childhood mortality. These both show a peak in mortality at the age range of 7.5–11 years—that is, in children before puberty (Supplementary Note 1). In an analysis of mortality profiles across mid-Holocene Cis-Baikal hunter-gatherer cemeteries, these two cemeteries are clearly outliers in terms of the proportion of childhood deaths (Fig. 4). This result was found to be highly statistically significant given a null model of mortality profiles (Supplementary Note 5). Conversely, the 20–25-year age range shows the lowest mortality at Ust’-Ida I, and deaths between 20–35 years of age are completely absent at Bratskii Kamen (Supplementary Fig. 4). Parents are also conspicuously absent from the pedigree groups; although there are many sibling and cousin relationships, there is only one instance of a parent–offspring relationship. The sex ratio in these individuals appears unaffected however (22 XY and 24 XX).

In the context of widespread infection with a plague strain of unknown virulence, this differential mortality between children and adults might be interpreted in a number of ways, given the available bioarchaeological data and current understanding of human immunity. First, adults could largely consist of those who had already been exposed to and recovered from the plague as children, and consequently acquired protective immunity, preventing reinfection or death. This would imply that outbreaks were regularly recurring, which our findings are not able to attest to, and would also imply that older individuals would be more likely to have acquired immunity, yet mortality actually increases slightly after the age range of 20–35 years (after the primary peak around 10 years of age). Alternatively, variation in mortality due to behavioural differences between age groups (for example, division of group tasks or roles by age, resulting in higher childhood exposure to marmots) cannot be ruled out, although there is little analogous precedent for this with regard to marmots specifically, and this is not supported by the lack of heightened childhood mortality in any other Baikal hunter-gatherer cemeteries (Fig. 4). Finally, it is possible that children could be at a greater risk of death owing to inherent differences in immune responses between adults and prepubescent children. Children are known to be more susceptible to infection from Gram-negative bacteria^42^, as evidenced by the epidemiological profile of *Yersinia enterocolitica* and *Y. pseudotuberculosis* infections today^43^.

## Functional variants in basal *Y. pestis*

**Fig. 5:**
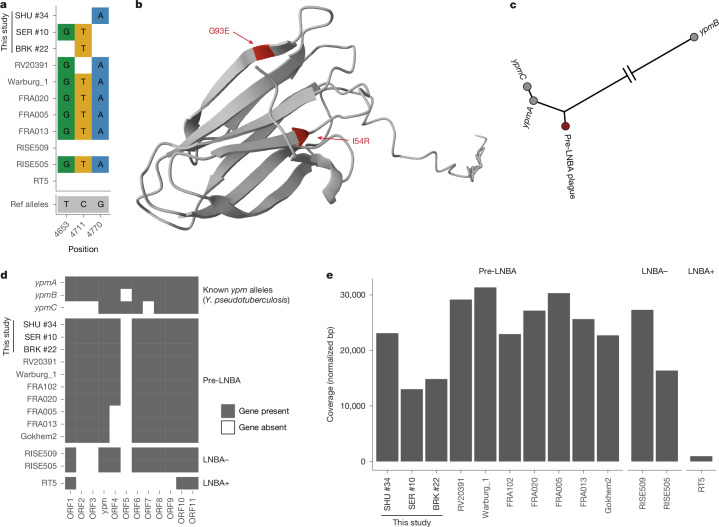
Variation around the *ypm* locus. **a**, SNPs within the *ypm* gene (missing genotypes shown as blank). Ref, reference. **b**, Positions of SNPs (red arrows) on the protein structure of YPMa (Protein Data Bank: 1PM4). This figure was made using data from the AlphaFold database, accession A0A0U1QV71. **c**, Unrooted neighbour-joining tree from the gene sequences of *ypm* variants, including the pre-LNBA plague form described here. **d**, Presence of open reading frames around the *ypm* locus. **e**, Comparison of ancestral gene content between prehistoric plague strains from reference graph alignments, using normalized breadth of coverage.

The evolution of *Y. pestis* lineages is shaped considerably by processes of gene loss^44^, a pattern typical of pathogenic bacteria in the transitional process to obligate parasitism, which has also been identified across the LNBA− plague strains^3^. From analysis of the coverage of the classic plague virulence genes, we find that virulence genes absent in published LNBA− and pre-LNBA strains from Riņņukalns (RV 2039) and Falbygden^11^ are also absent at Baikal (*ymt* and *YpfΦ* prophage; Fig. 5), prohibiting the manifestation of bubonic plague (Extended Data Fig. 3). However, since this virulence gene analysis relies on traditional single-reference mapping, it is restricted to genetic content that is present in the modern reference. To characterize possible ancestral *Y. pseudotuberculosis* variation in the Cis-Baikal strains which might contribute to our interpretation of their pathogenicity, we mapped sequenced reads to a pan-genome variation graph representing genetic diversity across 82 complete assemblies of the *Y. pseudotuberculosis* species complex (56 *Y. pestis*, 24 *Y. pseudotuberculosis* and 1 *Yersinia* *similis*, based on ref. ^11^). We found that the two plague strains from Lake Baikal carried similar levels of ancestral *Yersinia* diversity that were only found in *Y. pseudotuberculosis* and *Y. similis* as other pre-LNBA strains (Fig. 5e). For example, we detected the presence of *ypm*, the gene encoding the YPM superantigen known from modern-day *Y. pseudotuberculosis* strains^45^, and recently observed in pre-LNBA and LNBA− plague strains^11^. Three alleles of this gene exist in modern *Y. pseudotuberculosis*: *ypmA*, *ypmB* and *ypmC*, with *ypmA* being regarded as the most virulent form of the gene^46^.

YPM binds to the invariant region of human leukocyte antigen (HLA) class II molecules and interacts with the variable domain of the β chain of the T cell receptor. By bridging HLA class II and a T cell receptor, YPM promotes T cell activation and the release of a range of proinflammatory cytokines, further amplifying the immune response^47,48^.

These YPM-associated immune responses have been suggested to be the cause of various inflammatory complications, including encephalopathy, Far East scarlet-like fever (FESLF; also known as Izumi fever; especially associated with *ypmA*) and a Kawasaki-like syndrome^47,49–51^. Today, FESLF occurs primarily in children aged less than 14 years and Kawasaki disease occurs mostly in children aged 5 years or less. However, Kawasaki disease following *Y. pseudotuberculosis* infection may also affect older children^52^. These YPM-related inflammatory complications are likely to also have primarily affected prepubescent children in the past, further exacerbating the early *Y. pestis* induced morbidity and mortality in the young.

Notably, we find that the *ypm* gene from the two plague strains from Cis-Baikal is closest in sequence similarity to *ypmA*, differing at only 3 base positions: 4653 (T>G (isoleucine>arginine)), 4711 (C>T (synonymous threonine)) and 4770 (G>A (glycine>glutamate); Fig. 5a). These three single nucleotide polymorphisms (SNPs) appear to be fixed in all plague strains in which the gene is present (pre-LNBA and LNBA− strains; Fig. 5a). Since two of these three variants are non-synonymous mutations (I54R and G93E are located in distinct beta-sheets of the YPM structure), they could potentially influence protein secondary, tertiary or quaternary structure, protein–protein interactions, and recognition by the immune system (Fig. 5b). Furthermore, by reconstructing the most likely phylogeny of the three known *ypm* variants together with our data, we found that the *ypmB* version of the gene is highly divergent from *ypmA*, *ypmC *and the ancient plague *ypm*. Under the assumption that the root of the tree is located between *ypmB *and the remaining diversity, the *ypm* from ancient plague appears to diverge ancestrally to both *ypmA* and *ypmC* (Fig. 5c).

In addition, we identified ten open reading frames (ORFs) around the *ypm* locus that are present in the ancestral form of the plague but are absent in later forms. The ORF region is similar to an unstable region of the *Y. pseudotuberculosis* genome with notable low GC content. We found that these ORFs in the Baikal *Y. pestis* genomes were similar to those surrounding the *ypmB* variant as reported in other pre-LNBA strains^11^ (Fig. 5d). This pattern with a *ypm* gene similar to *ypmA* combined with a *ypm* locus similar to *ypmB* has—to our knowledge—not been observed previously. A possible explanation could be that this diversity in the Lake Baikal pre-LNBA strains, the most basal of the sampled plague strains, reflects ongoing local adaptation to marmots and other rodent hosts to a greater extent than humans because the regional animal host reservoir presumably at this time far exceeded that of humans. This, so far unique, combination might affect, for example, gene methylation and *ypm* transcription levels.

These genetic features of the Baikal pre-LNBA strains might, together with age-dependent differences in the immune system, partly help explain why prepubescent children predominate among the plague victims, although assessing their actual impact requires functional studies.

## Discussion

Our findings demonstrate that the earliest known outbreaks of plague occurred in prehistoric hunter-gatherers centuries before infections are observed in Neolithic farmers. These outbreaks were probably the result of zoonotic spillover from wild marmot populations at Lake Baikal. These results support a central or northeast Asian origin for plague, whereas previously the earliest samples had only been reported in northern Europe^11,15^. This is in line with estimates based on the analysis of modern *Y. pestis* diversity^53^. Our phylogenetic analysis reveals that these virulent plague strains are temporally relatively close to the most recent common ancestor of *Y. pestis* and *Y. pseudotuberculosis*, possibly indicating rapid diversification with the transfer to rodent hosts from one or several of the other animal hosts of *Y. pseudotuberculosis*. Additionally, this raises questions around the differentiation of taxa within the *Y. pseudotuberculosis* species complex (which includes *Y. pestis* and *Y. similis*) that ancient genome data alone may not be adequate to answer (given that conventional distinctions can be based on pathogenic potential and host range as well). Furthermore, the inferred high lethality of these outbreaks is directly evidenced by mortality profiles and coinciding radiocarbon dates in affected burial sites, indicating that children and adolescents were especially vulnerable; these are insights that were previously lacking for prehistoric plague infections. Until now, the earliest detected strains of plague were of uncertain pathogenicity; their virulence has been the focus of considerable debate, based on genetic data alone^3,10,15,16,54^. Here we have integrated multiple lines of evidence from mortuary sites affected by plague (including plague genomes, biological kinship patterns, mortality profiles and modelled radiocarbon date ranges) to characterize what we argue are the lethal consequences of infection during this period.

The context of these outbreaks is important for the interpretation of health and epidemiology in the past. That these outbreaks occur in relatively small, mobile prehistoric hunter-gatherer groups emphasizes that increased population density, animal domestication and lifestyle changes resulting from the Neolithic transition are not necessary conditions for significant zoonotic outbreaks. This further revises interpretations of plague as a unique factor contributing to demographic decline during the Late Neolithic in Europe, as previously suggested^10,11^, especially given the apparent severity of outbreaks identified here. The mortality profile at Baikal also contrasts sharply with the expectation of proponents of the Neolithic epidemiological transition theory that the greatest burden of zoonotic disease in prehistoric hunter-gatherers would fall on producers (20 to 40 year olds)^55^. Our findings further reveal insights into the social dimension of these communities during outbreaks, evidencing care for the dead (from the co-interment of close relatives and apparently contemporaneous victims), and concurrently detected infections among kin which evince interpersonal contact in life. This evidence of person-to-person transmission contrasts with previous expectations for basal plague strains^15^.

Of note, children seem to have borne the brunt of lethality from plague infections at Cis-Baikal. Different mortality rates among age groups have been observed in historical records of plague outbreaks. Similar to our findings, Parish records from the bubonic plague outbreak in London (UK) in 1603 showed a considerably higher child mortality rate (around 5× higher)^56,57^. One distinct difference between the Cis-Baikal outbreaks and mediaeval bubonic plague epidemic is the probable transmission route. Although airborne and faecal–oral transmission might have occurred in both, flea bite transmission associated with bubonic plague is unlikely in the Cis-Baikal outbreaks (given the absence of *ymt*). Spread of infectious droplets or aerosols through coughing is documented as the primary transmission mode of pneumonic plague^58^, matching our findings for human-to-human spread inferred from the biological kinship and archaeological data. Notably, our results are consistent with previous interpretations that early *Y. pestis* strains could have presented as fatal respiratory pathogens^54^.

Our results indicate that the earliest observed zoonotic spillover was not a one-off event but re-occurred several centuries later, highlighting the prominence of zoonotic infections in prehistoric communities across many different cultural and environmental settings. Additionally, a low-coverage identification of brucellosis suggests evidence of animal-to-human zoonotic transmission in these groups (infection is acquired by direct contact with infected animals^59^, Supplementary Note 3). Recurrent outbreaks of ancestrally diverged plague strains in Cis-Baikal groups between 5500–5000 cal bp further suggests a long history of wild rodents as a perennial reservoir for plague spillover. The subsequent plague strains genetically closest to those at Cis-Baikal are from around 5,000 km west in northern Europe. Given this distance, and that there is little evidence for external contact with non-hunter-gatherer groups at this time, this supports the hypothesis that a substantial, continent-spanning rodent reservoir of *Y. pestis* might account for frequent isolated spillover events in the subsequent millennia, instead of continuous human-to-human transmission. Moreover, the potential association of prehistoric spillover with human procurement of marmots at Cis-Baikal emphasizes the probable key role of rodent species other in the composition of *Y. pestis* reservoirs. Some 352 reservoir species have been identified from present-day surveillance, a number of which are ecologically long established (for example, ground squirrels and gerbils)^60^. The scenario of a persistent prehistoric reservoir aligns both with previous findings of rapid repeated infections of diverged plague strains within the same familial lineage from Neolithic Sweden^11^ as well as at Cis-Baikal. We note that recent findings show that early plague cases in domesticates^61,62^ may equally be congruous with reverse zoonosis, although given the long history of rodent species reported as reservoirs for plague^60^, we feel that this currently remains the most parsimonious source for these outbreaks.

Together, our findings underscore the universality of zoonotic infection, given the markedly different lifeways of prehistoric hunter-gatherers from European Neolithic farmers. These insights are as relevant for the challenges faced by the world today as they were 5,500 years ago, with 75% of new human pathogens emerging from animal transmission^63^. Insights into the evolutionary history of pathogens across periods of substantial demographic and technological change (before the impact of the Neolithic transition in this case) can provide data to contextualize the major challenges humanity is currently facing, such as the climate change-driven disruption of ecological niches around the world^64^.

## Methods

### Laboratory work

Ancient DNA was extracted from the dental cementum of molar or premolar teeth from archaeological skeletal remains studied by the Baikal Archaeology Project. Sampling for ancient DNA (aDNA) was undertaken in dedicated clean laboratory facilities at the Lundbeck Foundation Centre for GeoGenetics (Copenhagen) and at the Institute of Archaeology, University College London (London). Cementum was isolated specifically from the roots of teeth^67^ using a sterilized handheld rotary saw, and pulverized prior to demineralization and enzymatic digestion. Sampled aliquots were approximately 50–100 mg of material. Extraction, purification and library preparation of aDNA for shotgun sequencing followed the approach described in Allentoft et al.^68^, using a double-stranded library protocol following Margaryan et al.^69^ in the first instance, and the ‘Santa Cruz Reaction’ single-stranded library protocol^70^ for samples with low template DNA content. Concentrations for resulting libraries were obtained using an Agilent FragmentAnalyzer and pooled at equimolar concentration for sequencing on Illumina NovaSeq 6000 S4 flowcells (100 bp paired-end reads) at the GeoGenetics Sequencing Core (Copenhagen). All samples were screened without partial Uracil-DNA Glycosylase (UDG) treatment, and in some cases subsequent additional libraries were built with UDG treatment (following^71^).

For libraries where screening sequencing indicated the presence of *Y. pestis* DNA, in-solution capture enrichment was undertaken. Hybridization capture was performed using the Arbor Sciences myBaits kit following Wagner et al.^72^, using the manufacturer’s High Sensitivity protocol, but only with a single round of enrichment. Pooled libraries from the capture reactions were then re-amplified for 16 cycles and sequenced on the same platform as above.

### Preliminary bioinformatics

Following base-calling of Illumina data using CASAVA (v.1.8.2)^73^, adapter sequences and polyN tails were trimmed from demultiplexed fastq files using AdapterRemoval (v.2.0). Reads were aligned to the human reference genome GRCh38 using bwa aln (v.0.7.18)^74^ (reference genome hg19 was also used for hapRoH analysis, see below). Aligned reads were converted to BAM files, merged across libraries at sample level, sorted, filtered and indexed using Samtools (v.1.21)^75^, then duplicates identified using MarkDuplicates from Picard (v2.18.7), with the following options in place: ‘OPTICAL_DUPLICATE_PIXEL_DISTANCE = 12000 REMOVE_DUPLICATES = false TAGGING_POLICY = All VALIDATION_STRINGENCY = LENIENT’. Duplicate reads were then filtered out using Samtools alongside reads with a mapping quality of <30. Summary statistics for sequencing depth and coverage were generated using BEDtools (v2.23.0)^76^ and pysam (https://github.com/pysam-developers/pysam). Estimation of human DNA contamination and damage patterns were performed at a library level, using contamMix^77^, ANGSD (v.0.940)^78^, and mapDamage2.0^79^.

### Human DNA Analysis

Chromosomal sex was inferred based on the ratio of Y and X chromosome aligned reads, following existing confidence intervals^80^. Chromosomal aneuploidies were not detected. Mitochondrial haplogroups were assigned using haplogrep (v.2.4.0)^81^ following annotation of variants using mutserve (v.1.3.0)^82^. Y chromosomal haplogroups were assigned following the approach in ref. ^11^.

For exploratory analysis of ancestry through principal component analysis (PCA), pseudohaploid genotypes were called by randomly selecting a variant from a pileup generated with Samtools. Samples were then projected into the variation space obtained from using smartpca^83^ to undertake PCA on 2,086,279 SNPs (filtered for transversions only and with minor allele frequency >0.1%) from a reference panel of ancient Eurasian populations^68^. The latter was lifted over from hg19 using hgLiftOver (https://genome.ucsc.edu/cgi-bin/hgLiftOver), and the effects of liftover evaluated (Extended Data Fig. 2). All reported samples were included in the PCA by projection.

Diploid genotypes were called using bcftools (v.1.21), and for the analysis of IBD segment sharing, missing diploid genotypes were imputed using GLIMPSE^84^ (for samples with a minimum autosomal genome coverage of 0.1×) following the approach in ref. ^68^, and IBD segments called using IBDseq (v.r1206)^85^, followed by genetic clustering by IBD^68^.

Runs of homozygosity were detected from homozygous-by-descent segments obtained from IBDseq, and from pseudohaploid data subset to the 1240k SNP positions using hapRoH (v.1)^86^. Biological kinship was inferred using KIN^35^, initially running KINgaroo on filtered BAM files targeting the 2,086,279 SNPs described above, and then validated based on IBD sharing inferred from IBDseq. Pedigrees were then reconstructed taking into account the resulting log-likelihood estimates for kinship scenarios, uniparental haplotypes, sex and age-at-death (Supplementary Note 2).

### Screening for pathogen taxa

Shotgun sequencing data generated from dental cementum was screened for the presence of known human pathogens using the pathopipe workflow (https://github.com/martinsikora/pathopipe/) detailed in Sikora et al.^87^. Reads were classified using a fast k-mer approach, KrakenUniq^88^ (v.0.5.8), based on a custom database of human pathogens and environmental microbes. For each genus identified in each sample, pairwise alignments using bowtie2^89^ (v.2.5.4) were made for all reads classified to that genus against all available species reference genomes for the same genus. Assignments are then made for the presence of pathogen taxa on the basis of the following detection thresholds: unique read count >30, k-mer rank = 1, corrected coverage ratio >0.5, average nucleotide identity >0.97, average number of soft clipped bases <8, based on those applied by Seersholm et al.^11^.

### *Y. pestis* DNA analysis

For samples where plague infection was identified through the pathogen screening pipeline, we carried out traditional single-reference mapping with Bowtie2^89^ against the plague reference genome (CO92; GCA_000009065.1), with the parameters ‘-D 20 -R 3 -N 1 -L 20 -i S,1,0.50–end-to-end–no-unal’. Next, duplicate reads and low mapping quality reads (MQ < 30) were removed with samtools^75^, followed by calculations of the average depth of coverage in each sample using BEDtools genomecov^76^. We characterized plague cases based on their coverage as either: tentative detections (<0.01×), lower-coverage plague cases (0.01-1×) or higher-coverage plague genomes (>1×).

For the three higher coverage genomes we called genotypes in a sample-wise manner using HaplotypeCaller from GATK^90^, followed by a subsequent step of joint haplotype calling using GenotypeGVCFs on the merged dataset. Using VariantFiltration (GATK) we removed low-confidence calls of either: low genotype quality (<50), an allele balance of less than 0.9, a read depth of less than 3 or a read depth higher than 1,000. Next, we converted the dataset to multifasta format using bcftools consensus. In doing so, we applied a mask across regions containing the highest proportions of zero mapping quality reads, typically localized in repetitive regions, following the approach in Seersholm et al.^11^. To maintain the coordinates of the reference genome and for consistency with the other aligned samples, sequencing data for published reference genomes for *Y. pseudotuberculosis* and *Y. similis* were downloaded from ENA and the reads were realigned to the *Y. pestis* reference genome GCA_000009065. Genotypes were then called and filtered as described above, yielding a multiple sequence alignment for the full * Yersinia* chromosome with 448 sequences on the coordinates of the reference genome. A phylogenetic tree was inferred from the full alignment file including all reference sequences and the three high-coverage samples from this study using RAxML-NG^91^ with the GTR + G substitution model and using the *Y. similis* reference genome (SAMEA5779183) as an outgroup (see Extended Data Fig. 3). We converted the multiple sequence alignment to a haploid VCF using faToVcf^30^ with the *Y. pestis* reference genome NC_003143.1.fa as a reference, then built a mutation-annotated tree object from this VCF and the RAxML phylogeny using UShER^30^. This new UShER phylogeny maintains the original topology but directly assigns substitutions in the VCF to branches on the tree using the Fitch-Sankoff algorithm^92,93^, so that edge lengths are in units of real substitutions. We used matUtils^94^ to extract a.json file from the mutation-annotated tree protobuf, available interactively online at https://bit.ly/Ypestis_MAT.

For the 8 lower coverage samples, we called a SNP-only vcf using bcftools, filtering for a minimum mapping quality of 30 and bases, a minimum base quality of 30, and a maximum depth of 1,000. We only kept sites which were variable in the reference panel or in which more than one lower coverage sample had a variant called, and further removed all variant sites in the low mapping quality mask described above, yielding a filtered vcf for the lower coverage samples. This low-coverage vcf was used to phylogenetically place the low-coverage samples into the mutation-annotated tree using UShER. All 8 lower coverage samples shared a single, maximally parsimonious placement at the root node of the *Y. pestis* clade.

Finally, we ran Gubbins (v.3.4.3)^31^ on a multifasta of all LNBA− and pre-LNBA genomes, as well as the O:1c serovar genome (SAMEA7160327) and the SAMN03121000 genome as outgroup (see Supplementary Fig. 14). To date the phylogeny, while taking recombination into account, we ran BactDating^32^ on the output tree from Gubbins based on non-recombinant variation. We used 100,000 iterations and a relaxed gamma model as suggested in ref. ^33^. Convergence was confirmed through the trace file as shown in the Supplement. We report median age estimates and 95% confidence intervals for nodes of interest in Fig. 2.

### Variation graph analysis

To characterize the full diversity of ancient plague we built a pan-genome variation graph of all known diversity within the *Y. pseudotuberculosis* species complex (*Y. pestis*, *Y. pseudotuberculosis* and *Y. similis*). We used Pangenome Graph Builder (pggb)^95^ on all available *Y. pseudotuberculosis* complex assemblies from NCBI with assembly level characterized as either ‘chromosome’ or ‘complete’. To ensure correct construction of the graph around the plasmids, we built separate graphs for the chromosome and the plasmids and merged these afterwards using vg tools^96^. Next, we indexed the graph and carried out Giraffe^97^ short read mapping to the variation graph of the data from this study and all publicly available ancient shotgun data. Lastly, we identified graph nodes present in the Lake Baikal plague strains but absent in all modern plague assemblies and classified these based on their presence/absence pattern in *Y. pseudotuberculosis* and *Y. similis*. Each node was classified as either ancestral (present in both *Y. pseudotuberculosis* and *Y. similis*) or either *Y. pseudotuberculosis*-derived or *Y. similis*-derived.

### Age-at-death estimation

Age-at-death estimation was based on a variety of established anthropological methods. For non-adult individuals (generally <20 years), it was assessed through dental formation and eruption, epiphyseal and long bone diaphyseal measurements, and epiphyseal union, as summarized in refs. ^49–51,98,99^. Adult age estimation focused on skeletal morphological changes, namely those of the pubic symphysis^100,101^ and iliac auricular surface^102–104^, but also palatine and ectocranial suture closure^105–107^. For all individuals, as many methods as possible were considered based on the state of skeletal and/or dental preservation.

### Radiocarbon dating

Radiocarbon dating was undertaken at the Oxford Radiocarbon Accelerator Unit following an established protocol at that facility^108^. New determinations from Bratskii Kamen, Serovo and Shumilkha are presented here for the first time, alongside previously published radiocarbon dates^27,109^ (Extended Data Fig. 4 and Supplementary Table 1). All human dates are corrected for the freshwater reservoir effect (FRE), using the regression equation for southwest Baikal/Angara^110,111^ (Supplementary Note 4). A small number of dates on red deer tooth pendants are preferred over human dates where available, as they avoid the FRE. Bayesian modelling of the radiocarbon dates was undertaken in OxCal 4.4^65^, using non-informative, single-phase models with uniform boundaries. To visualize summed multiple dates, kernel density estimation (KDE) models and plots within Bayesian models were employed^112^.

### Reporting summary

Further information on research design is available in the Nature Portfolio Reporting Summary linked to this article.

## Online content

Any methods, additional references, Nature Portfolio reporting summaries, source data, extended data, supplementary information, acknowledgements, peer review information; details of author contributions and competing interests; and statements of data and code availability are available at 10.1038/s41586-026-10540-5.

## Supplementary information

Supplementary Information This file contains Supplementary Notes 1–6, including Supplementary Figs. 1–39, legends for Supplementary Tables 1–7 and additional references.

Reporting Summary

Supplementary Tables Supplementary Tables 1–7

## Data Availability

Raw sequencing data generated for the analysis of ancient genomes here is available on European Nucleotide Archive under accession PRJEB111316. Aligned sequences and the mutation annotated tree described here can be accessed at https://github.com/ramacleod/Prehistoric_plague_MAT.
