# Supplementary material for: Lethal plague outbreaks in Lake Baikal hunter-gatherers 5,500 years ago
Source: Nature. 2026 Jun 17;654(8119):697–705. doi: 10.1038/s41586-026-10540-5 (PMC13275307; doi:10.1038/s41586-026-10540-5)
Supplement: Supplementary file 1 — This file contains Supplementary Notes 1–6, including Supplementary Figs. 1–39, legends for Supplementary Tables 1–7 and additional references. [file 41586_2026_10540_MOESM1_ESM.pdf]

nature portfolio

https://doi.org/10.1038/s41586-026-10540-5

Supplementary information

Lethal plague outbreaks in Lake Baikal hunter-gatherers 5,500 years ago

In the format provided by the authors and unedited

Supplementary Information for ‘Lethal Plague Outbreaks in Lake Baikal Hunter–gatherers 5500 Years Ago’

Supplementary Notes 1-6
Note 1: Archaeological Context; Note 2: Ancient Human Genome Analysis; Note 3: Ancient Pathogen DNA Analysis; Note 4: Radiocarbon Chronology; Note 5: Mortality Profile Modelling; Note 6: The Effects of hg19 to hg38 Liftover.

Supplementary Data

Supplementary Data 1. Genetic and archaeological metadata of ancient individuals, and reported radiocarbon dates.

Supplementary Data 2. Plague detection results from screening of shotgun sequencing data.

Supplementary Data 3. Other pathogen detection results from screening of shotgun sequencing data.

Supplementary Data 4. Results of pairwise IBD-sharing.

Supplementary Data 5. Pairwise kinship results

Supplementary Data 6. DNA sequencing library information

Supplementary Data 7. Radiocarbon dating and stable isotopic data

Contents

Supplementary Note 1: Archaeological Context **5**

Figure S1: Site map of excavated graves at Shumilkha, indicating its location on the Angara River. From Svinin 198113, page 53. 7

Figure S2: Features of the Serovo mortuary grouping at Bratskii Kamen. a, plan of Grave 7, featuring a bow above the skeleton; b, composite point with lithic inserts; c, clay vessel. Redrawn by V. Bazaliiskii after Okladnikov15. 8

Figure S3: Mortality profile inferred from age-at-death estimates for Late Neolithic individuals at Ust'-Ida I. 9

Figure S4: Mortality profile inferred from age-at-death estimates for Late Neolithic individuals at Bratskii Kamen. Data for 12 of the 20 individuals provided by NN Mamonova. 9

Figure S5: Mortality profile inferred from age-at-death estimates for Late Neolithic individuals at Serovo. Data for 7 of the 16 individuals provided by NN Mamonova. 10

Supplementary Note 2: Ancient Human Genome Analysis **10**

Figure S7. Proportions of uniparental haplotypes observed in the 46 (24 XX, 22 XY) ancient individuals reported here. 11

Figure S8: Summed RoHs from Ust'-Ida and Shumilikha cemeteries, generated from hapRoH and IBDseq. No RoHs >2cM were identified from individuals at Bratskii Kamen or Serovo. 12

Figure S9: Total IBD-sharing between pairs of individuals in this study. IBD sharing was calculated using IBDseq and each pair is coloured by their relationship as inferred by KIN. One clear outlier is observed where KIN and IBDseq disagrees: the putative avuncular relationship between CGG024591 and DA355 (circled with a stippled line). This relationship was further investigated by the tools KING and ngsRelate which both inferred CGG024591 and DA355 to be unrelated. Accordingly this relationship has been manually changed from 'avuncular' to 'unrelated' in Supplementary Table 5. 14

Figure S10. Outgroup F3 statistics of the form $f3$(outgroup; Baikal plague group, listed test group;) comparing our test samples with IBD groups from Allentoft et al. 2024. As the outgroup we used the IBD group '5.1_Africa_SAPast_1'. Only the 20 closest populations to our samples are shown. 15

Supplementary Note 3: Ancient Pathogen DNA Analysis **16**

Table S1. Coverages for plague genomes with sufficient data to be included in the phylogeny (Fig. 2). 17

Figure S12: Blast analysis of all (96) Brucella reads from CGG024495. Bars represent the total number of reads with a best match to each species. Only the most common species are shown. 19

Figure S13: The trace from BactDating after 100,000 iterations. This shows convergence for the estimate of molecular divergence dates. Iterations were sampled every 1000 iterations. 21

Figure S14: Recombination events within Y.pestis and Y.pseudotuberculosis. Left hand side: Phylogenetic tree built on non-recombinant regions of each genome. Right hand side: Heatmap of recombination events within each genome. 21

Figure S15: Variant positions between the three higher coverage Lake Baikal genomes. Number of called SNPs differing between any given combination of two samples normalised to the total number of shared SNPs between those samples. 22

Figure S16: Positions differing between between BRK #22 and SER #10. Red bar

indicates the variant position. Visualised using IGV. 23

Figure S17: IGV visualisation of the three YPM variants identified. For all high coverage samples in this study - Shumilikha #34 (CGG024606), Serovo #10 (CGG024576) and Bratskii Kamen #22 (CGG024574) - all three variants within the YPM gene (positions 4653, 4711 and 4779), were visualised. Red bar indicates the variant position. Visualised using IGV. 24

Figure S18: EPA-ng placement of lower coverage plague detections from Lake Baikal. Each subplot depicts the placement of one lower coverage sample onto the plague phylogeny from Figure 2. Branch width and color indicate the likelihood weight ratio of the placement. 25

Supplementary Note 4: Radiocarbon Chronology 26

Equation 1. Regression equation for the correction of radiocarbon dates from human skeletal tissue in mitigation of the Freshwater Reservoir Effect in the Angara Valley region of Cis-Baikal. 26

Figure S19. Site-based groupings for all LN individuals positive for plague. Early phase is indicated in red, later phase in yellow. 27

Figure S20. Combined dates for early phase plague victims (n = 16) 28

Figure S21. Combined dates for late phase plague victims (n = 2) 28

Figure S22. Bayesian model for all Late Neolithic individuals with plague, divided into 'early' and 'late' phases. 29

Figure S23. KDE plots within Bayesian models for the early and late plague victims. The lightly shaded areas show the summed probability distributions prior to modelling; red indicates the earlier plague outbreak phase, whilst yellow indicates the later phase. 30

Figure S24. Modelled span of early plague victims. 30

Figure S25. Modelled difference between the early and late plague phases (using the 'Difference' function in OxCal 4.4). 31

Figure S26. Modelled difference between the last date in the early phase and the first date in the late phase of plague. The light gray shaded region shows the unmodelled probability distribution. 32

Figure S27. Combined dates for Ust'-Ida I (n = 36). This excludes three burials (#33.01, #56.02 and #44.02) identified in a Bayesian model as having low agreement indices. 32

Supplementary Note 5: Mortality Profile Modelling 39

Figure S28. Continuous null model of age-at-death. Fitted to n=625 individuals with osteometric age ranges. Best fitting model was a two-mixture model of a half-gaussian (SD = 0.201) and a truncated Gaussian (mean = 30.492, SD =14.883, zero probability for age<=0), with the half-gaussian comprising 16.63% of the mixture. 40

Figure S29. Age-at-death modelling for Bratskii Kamen (LN). Left: null distribution of mean age (top), test phase distribution of mean age (bottom). Right: null distribution of percentage of juvenile percentage (top), test phase distribution of juvenile percentage (bottom). 41

Figure S30. Age-at-death modelling for Ust'-Ida I (LN). Left: null distribution of mean age (top), test phase distribution of mean age (bottom). Right: null distribution of percentage of juvenile percentage (top), test phase distribution of juvenile percentage (bottom). 41

Figure S31. Age-at-death modelling for Kuzhir-Nuge XIV. Left: null distribution of mean age (top), test phase distribution of mean age (bottom). Right: null distribution of percentage of juvenile percentage (top), test phase distribution of juvenile

percentage (bottom). 41
Figure S32. Age-at-death modelling for Lokomotiv. Left: null distribution of mean age (top), test phase distribution of mean age (bottom). Right: null distribution of percentage of juvenile percentage (top), test phase distribution of juvenile percentage (bottom). 42
Figure S33. Age-at-death modelling for Lokomotiv Raisovet. Left: null distribution of mean age (top), test phase distribution of mean age (bottom). Right: null distribution of percentage of juvenile percentage (top), test phase distribution of juvenile percentage (bottom). 42
Figure S34. Age-at-death modelling for Sarminskii Mys. Left: null distribution of mean age (top), test phase distribution of mean age (bottom). Right: null distribution of percentage of juvenile percentage (top), test phase distribution of juvenile percentage (bottom). 43
Figure S35. Age-at-death modelling for Shamanka II. Left: null distribution of mean age (top), test phase distribution of mean age (bottom). Right: null distribution of percentage of juvenile percentage (top), test phase distribution of juvenile percentage (bottom). 43
Figure S36. Age-at-death modelling for Shumilikha. Left: null distribution of mean age (top), test phase distribution of mean age (bottom). Right: null distribution of percentage of juvenile percentage (top), test phase distribution of juvenile percentage (bottom). 44
Figure S37. Age-at-death modelling for Verkholensk. Left: null distribution of mean age (top), test phase distribution of mean age (bottom). Right: null distribution of percentage of juvenile percentage (top), test phase distribution of juvenile percentage (bottom). 44
Supplementary Note 6: The effects of hg19 to hg38 liftover 45
Figure S38. Distance between points mapped to hg19 and hg38, respectively. Histogram of the distances between points at PC1 to PC10. 46
Figure S39. D-stats comparing the effect of mapping to hg19 and hg38. Sample-wise D-statistics. 47
References 47

Supplementary Note 1: Archaeological Context

Hunter–gatherer cemeteries in the Cis-Baikal region (the area on the northwest side of Lake Baikal including the Angara River Valley and Upper Lena River) have been the focus of intensive archaeological research by the Baikal Archaeology Project since the mid-1990s, though most were first excavated in the 19th and 20th centuries, in particular under the Soviet archaeologist Alexei Pavlovich Okladnikov. Okladnikov defined the distinct cultural attributes of graves typologically as mortuary traditions[1]. His model was later revised based on direct radiocarbon dating of hundreds of human burials by the Baikal Archaeology Project[2,3], resulting in the following sequence: Late Mesolithic (*Khin* mortuary group), Early Neolithic (*Kitoi* mortuary tradition), Late Neolithic (*Isakovo* and *Serovo* mortuary traditions), and Early Bronze Age (*Glazkovo* mortuary tradition). As noted in the main text, the Neolithic in Siberian archaeology is defined, following the Russian archaeological tradition, on technological grounds, that is by the introduction of the bow-and-arrow, clay vessels, and groundstone technology. There is no evidence for formal cemeteries from the Middle Neolithic and so it is effectively lacking from this sequence of mortuary sites. The present study focuses on the following Late Neolithic (5600–5000 years cal. BP) burial sites: Ust'-Ida I and Shumilikha (*Isakovo* mortuary tradition), and Bratskii Kamen and Serovo (*Serovo* mortuary tradition). On the basis of previously published stable isotope data, some evidence of dietary trends in these groups have been identified, with the *Isakovo* sample deriving primarily from Ust'-Ida I (Angara river valley). Stable isotope data for *Isakovo* individuals from Ust'-Ida I demonstrate reliance on local middle Angara River fish and terrestrial game[4]. Data for the direct comparison of dietary isotopic trends between Late Neolithic cemeteries in the Angara River valley are limited but some new results have been recently obtained (including Bratskii Kamen, Serovo, and Shumilikha cemeteries) and will be published in near future.

Okladnikov originally believed that the *Isakovo* tradition preceded the *Serovo*[1], though these are now known to be contemporaneous[5]. *Isakovo* burials are predominantly found along the Angara River (which flows north from Lake Baikal and forms a tributary of the Yenisei), and many burials are oriented parallel to the river with heads pointing upstream although variation in this regard does exist. Bodies are positioned extended and supine, and cemeteries are typically of a small to medium size (fewer than 25 individuals). Most graves comprise single inhumations, though shared graves are not unusual, and may include children in particular. Graves display stone structures and grave good assemblages commonly consist of mitre-shaped clay vessels, in addition to lithic arrowheads, bone and antler points or composite tools (fishing gear is rare, despite the proximity to the river, and red ochre is absent).

Serovo burials are likewise distributed along the Angara River but also along the Upper Lena River (where they are sometimes referred to as the Archaic mortuary tradition[6]), as well as in the Little Sea microregion. The *Serovo* mortuary tradition is similar to *Isakovo* in terms of cemetery size (with the exception of Ust'-Ida I where its Isakovo component is the largest known of either tradition anywhere in Cis-Baikal), use of stone structures and extended supine body position, but many burials are instead oriented perpendicular to the river, with the head pointing away (but variations from this pattern are not uncommon), and there are differences in grave good assemblages. In particular, large lithic bifaces and egg-shaped clay pots appear to distinguish *Serovo* burials from *Isakovo*. Use of red ochre is

rare and limited mostly to isolated stains, and there is possibly a higher incidence of rich grave goods associated with *Serovo* burials than *Isakovo*.

Ust'-Ida I

Ust'-Ida I is a large cemetery located on the east bank of the Angara River approximately 250 km north of Lake Baikal, first identified from the discovery of a single grave by A.P. Okladnikov in the 1950s, and subsequently several more in the 1980s. The site was systematically excavated under the direction of Vladimir I. Bazaliiskii from 1987 to 1995, resulting in the discovery of multiple phases of use: Early Neolithic *Kitoi* with one grave; 32 Late Neolithic *Isakovo* graves (comprising 48 individuals, the most substantial component of the cemetery), and 18 Early Bronze Age *Glazkovo* graves (comprising 19 individuals). Unfortunately, this cemetery has not yet been published as a monograph and is known only from a short site report[7]. Comprehensive radiocarbon dating of the *Isakovo* component of this cemetery was undertaken by the Baikal Archaeology Project[3,4,8], resulting in direct dates for 36 individuals (see Supplementary Note 4). Stable isotope data indicate a significant dietary contribution from local fishes from the middle Angara[4]. Activity-induced dental modifications are observed exclusively in males at Ust'-Ida I[9]. These occlusal grooves are linked to the processing of fibres for the production of thread (possibly for the construction of nets). Grave goods are highly repetitive and include clay pots with net impressions, lithic arrowheads, ground adzes and knives made of slate and nephrite, bifacial triangular knives, composite insert tools, bone points of various forms, harpoons, and red deer canine pendants and a rare anthropomorphic figurine[7].

Genetic data have previously been published from this site in an early study of mitochondrial DNA haplotype variability, amplifying a 440bp region of the mitochondrial genome in 19 ancient individuals[10], and subsequently from the mtDNA coding region in 39 ancient individuals[11]. Ancient genome data were previously reported for four individuals[12], corresponding to Burials #22, #30, #56.01, and #14 (published sample numbers DA342, DA344, DA345, and DA355 respectively).

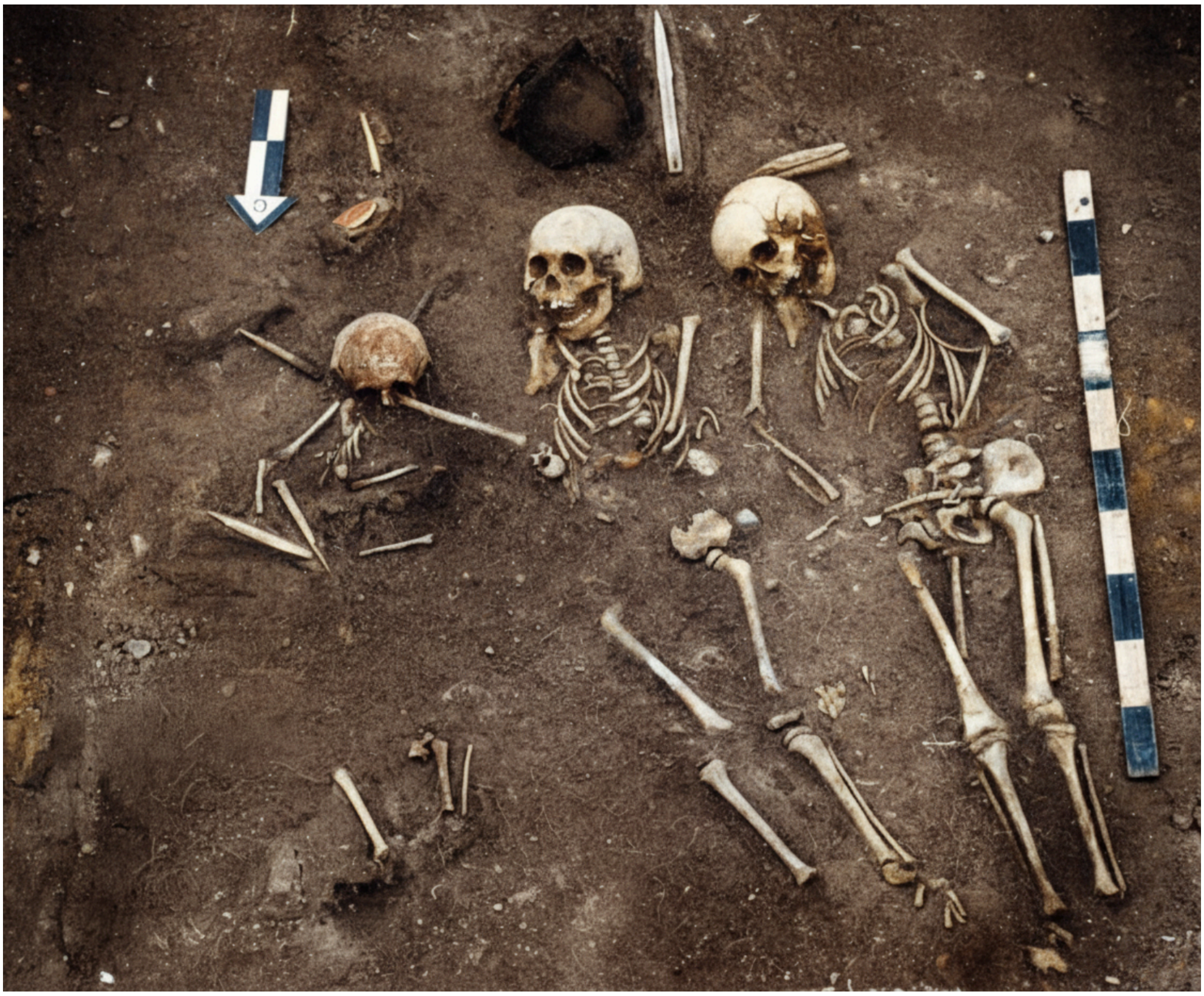

Figure S1: Features of the *Isakovo* mortuary grouping at Ust'-Ida I. Photograph from the excavation of Grave 25 at Ust'-Ida; note the presence of a pottery vessel above the head of the middle individual, ground stone tools, and worked bone points.

Shumilikha

Shumilikha, also sometimes referred to in the archaeological literature as Ust'-Belaia II (distinct from Ust'-Belaia), is located at the confluence of the Angara and the Belaia Rivers (Figure S2). This site was excavated in 1972–1973, and published in a monograph edited by V. V. Svinin in 1981[13]. It predominantly comprises Early Bronze Age *Glazkovo* graves (39 inhumations within 37 graves, with two instances of shared graves). Additionally, there are two single Early Neolithic *Kitoi* graves, and 10 burials across six Late Neolithic graves, three of which have been tentatively classified as *Isakovo*.

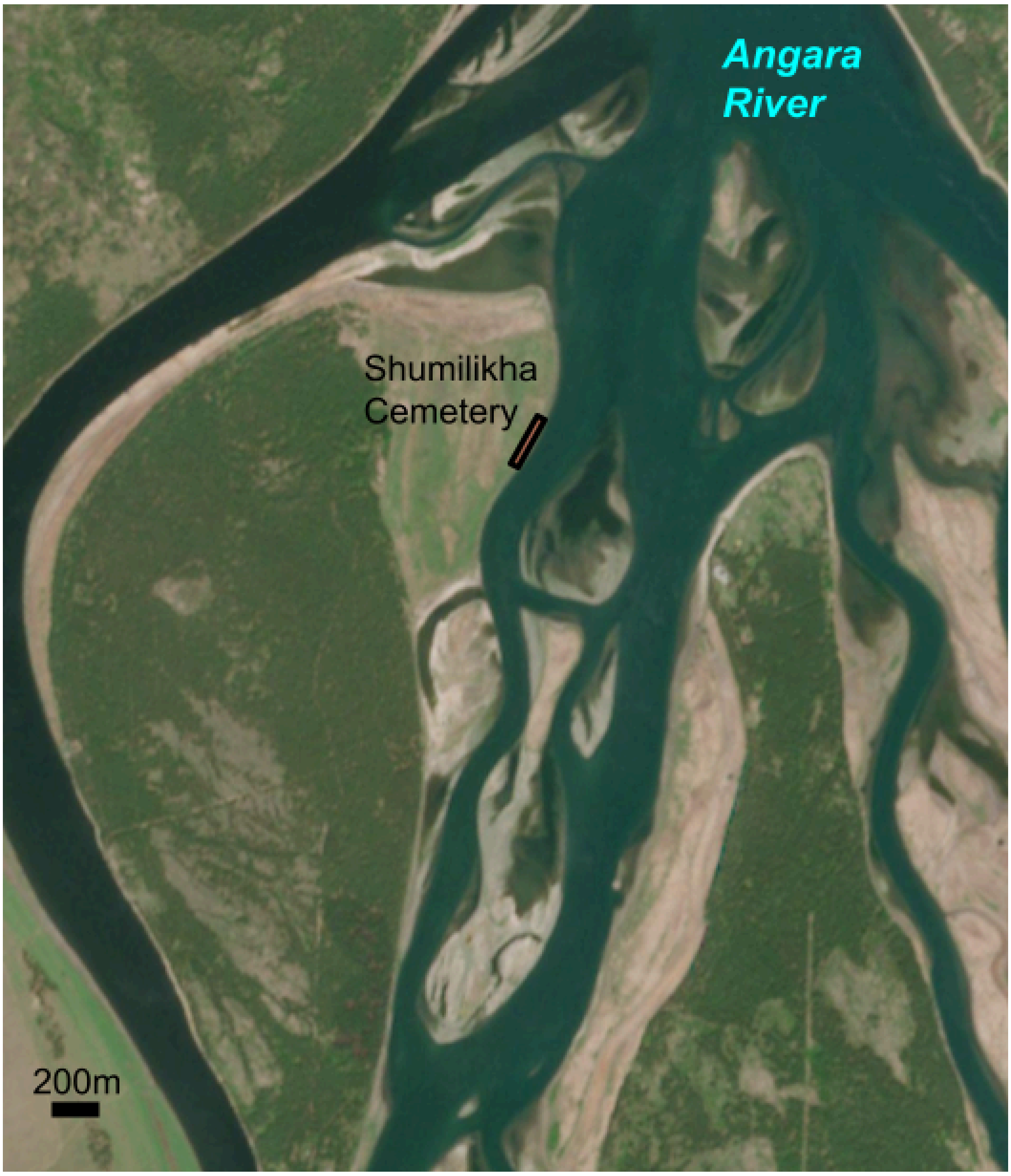

Figure S2: Site map of excavated graves at Shumilkha, indicating its location on the Angara River. Map created using Copernicus Sentinel-2 L2A data 2026, imagery from 2025-06-05, true colour (credit: European Union, processed with Copernicus browser). See also Svinin 1981[13], page 53.

Bratskii Kamen

The site of Bratskii Kamen is located south of the city of Bratsk in the now flooded area of the Bratsk Reservoir, which was dammed for a hydroelectric power plant completed in 1967. The site predominantly comprises Late Neolithic graves (21 individuals within 16 graves: three assigned to the *Isakovo* mortuary tradition and 12 to *Serovo*), though also includes several other graves: one Early Neolithic, one Early Bronze Age *Glazkovo* grave, and four additional graves lacking diagnostic characteristics (all single burial graves). The site was initially excavated in 1932, and excavated again in 1955–1956 by A. P. Okladnikov prior to the flooding of the area[14,15].

Serovo

Serovo (sometimes ‘Zverevo’) is the type site upon which the *Serovo* mortuary tradition is based. It is located on the west bank of the Angara river, 145 km north (downstream) of Ust’-Ida I, and over 200km south by southeast from Bratskii Kamen. Inhumations at Serovo were first excavated in the 1930s, and the site was extensively excavated in 1957 by A. P. Okladnikov[15]. Burials are predominantly in the Late Neolithic *Serovo* tradition (16 individuals in 15 graves), though with two Early Neolithic and two Early Bronze Age *Glazkovo* graves, and five missing contextual identification.

Bioarchaeology of Human Remains

Demographic profiles of the Late Neolithic remains from Ust’-Ida I, Bratskii Kamen, and Serovo (Figures S3–S5) reveal their unusual nature, all being dominated by non-adult individuals (those under the age of 20 years at death). In particular, 65–75% of individuals interred at Ust’-Ida I and Bratskii Kamen represent prepubescent children under the age of 15 years. While Serovo (Figure S5) has relatively fewer non-adult individuals (44%), their proportion is still considerably higher than is typical for the Middle Holocene Cis-Baikal cemeteries. As a comparison, non-adult individuals from EBA cemeteries in the region, such as Khuzhir-Nuge XIV and the EBA component of Ust’-Ida I, represent 26–33% of those interred[16–18]. Demographic data are not yet available for Shumilikha. Explaining the apparently high mortality of infants and children in the Late Neolithic is not possible from macroscopic analyses of human remains. Unfortunately, the vast majority of pathological conditions—especially acute infections causing death—do not affect skeletal and dental tissues[19]. Indeed, paleopathological data from these four sites reveal low frequencies of skeletal lesions, most representing degenerative changes, such as osteoarthritis, on adult individuals. Lesions consistent with physiological stress or “poor health” reflect chronic infections (e.g., sinusitis) or metabolic conditions, being documented on fewer than 20% of individuals at any site. Paleopathological data are similar among the four sites, and typical, again, for the Middle Holocene Cis-Baikal[16–18].

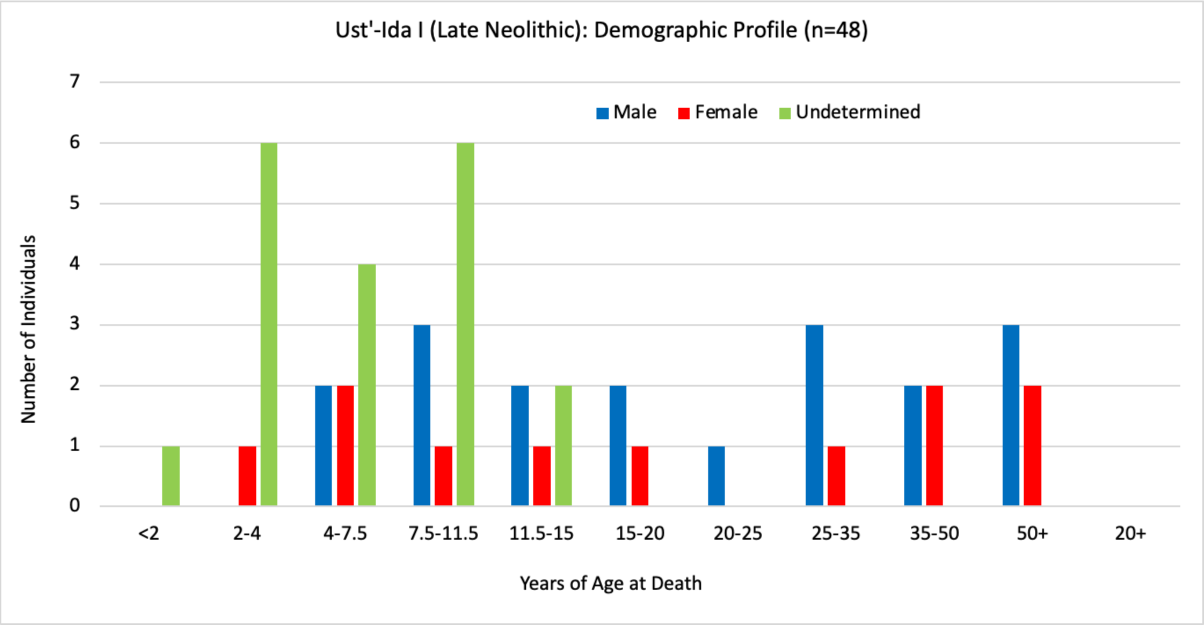

Figure S3: Mortality profile inferred from age-at-death estimates for Late Neolithic individuals at Ust'-Ida I.

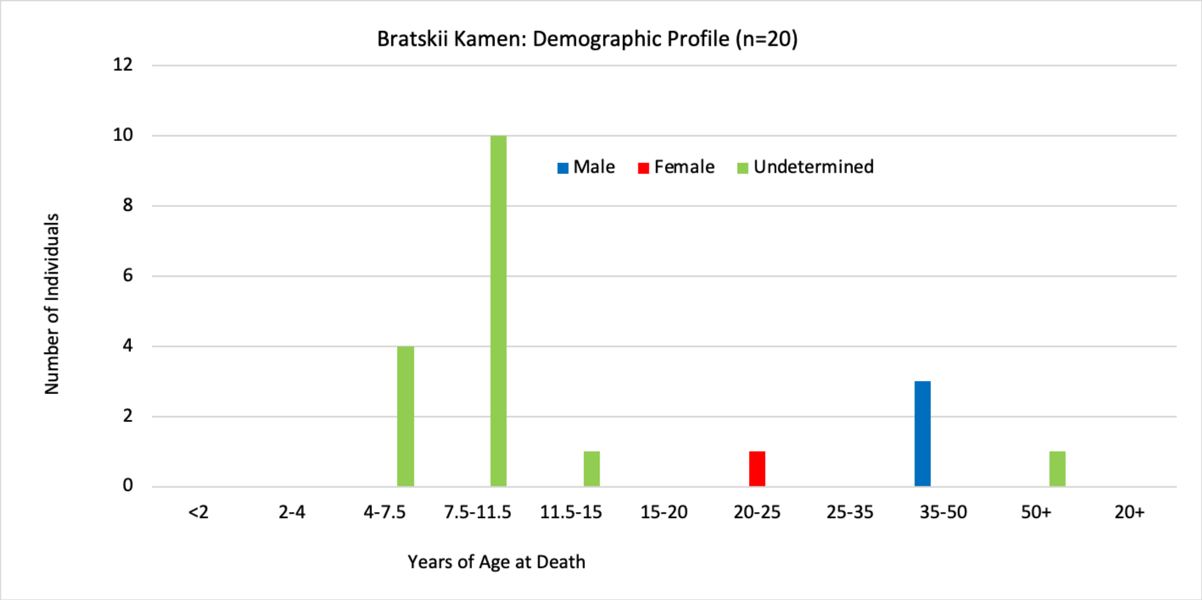

Figure S4: Mortality profile inferred from age-at-death estimates for Late Neolithic individuals at Bratskii Kamen. Data for 12 of the 20 individuals provided by NN Mamonova.

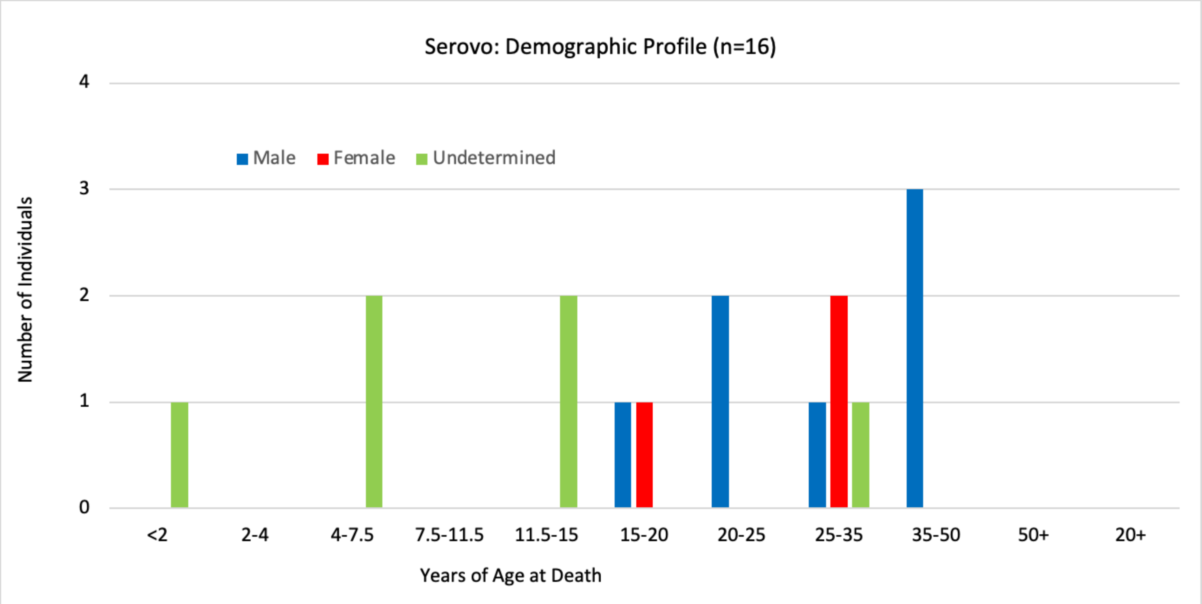

Figure S5: Mortality profile inferred from age-at-death estimates for Late Neolithic individuals at Serovo. Data for 7 of the 16 individuals provided by NN Mamonova.

Supplementary Note 2: Ancient Human Genome Analysis

Uniparental Haplogroups

Uniparental haplogroups were assigned using mutserve and haplogrep for mitochondrial haplogroups as detailed above, and an in-house pipeline described in Seersholm et al.[20] for Y chromosome haplogroups. For the latter, individuals identified as XY genetic sex (*n*=22) with Y chromosome coverage >0.002 had Y chromosome-mapped reads SNP-called using bcftools mpileup and call, and the resulting genotypes used to assign haplogroups based on the corresponding derived alleles observed in ISOGG subgroups. Assigned haplogroups, quality scores and Y haplogroup paths are included in the Supplementary Data.

Y haplogroups were dominated by subgroups of Q1b~ (see Figure S7), consistent with previous results showing the prevalence of this haplotype following the Middle Neolithic hiatus in the Cis-Baikal region[12] (Schulting et al. in prep.), Contrastingly, there is a much higher degree of mitochondrial haplotype diversity, even taking into account the higher sample size (*n*=46), an observation consistent with the scenario of male endogamy, and possible Y chromosomal bottleneck in the demographic history of this population.

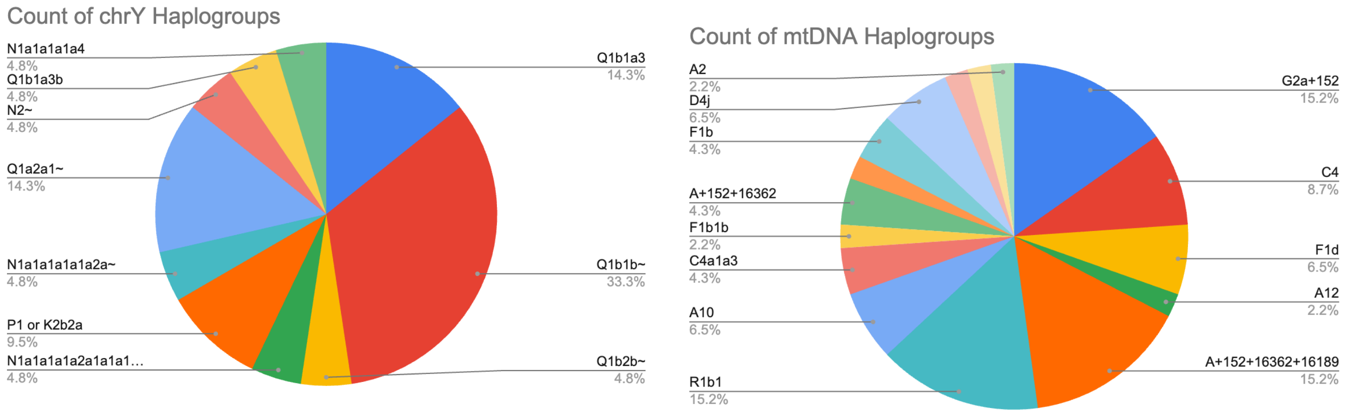

Figure S7. Proportions of uniparental haplotypes observed in the 46 (24 XX, 22 XY) ancient individuals reported here.

Inbreeding

Runs of Homozygosity (RoH) indicative of inbreeding were detected using two approaches: as Homozygous-by-Descent (HBD) segments reported by IBDseq based on imputed genotypes (see Methods section in main text), and with pseudohaploid genotypes by comparison with a large database of reference haplotypes using hapRoH. For hapRoH analysis, pseudohaploid genotypes were generated at the 1240K positions (autosomes only) using bcftools mpileup and call to create a VCF, and then vcf2eigenstrat from gdc (https://github.com/mathii/gdc). Samples with fewer than 400,000 SNPs represented were discarded from analysis. HapRoH primarily reports RoHs longer than 4cM, for which very few were detected across all the studied individuals (see Figure S8). Only five individuals had ROH segments longer than 4cM: Ust'Ida #25.03 (CGG024154) with 9.6cM; Ust'Ida #22 (DA342) with 8.6cM; Ust'Ida #56.01 (DA345) with 4.5cM; and Ust'Ida #14 (DA355) with 4.1cM. When the minimum RoH length threshold was reduced to 2cM, all individuals were

found to have RoHs in their genome, supporting the interpretation that this result is indicative of an overall large effective population size. This was estimated using the maximum likelihood approach in hapROH and was found to be 18,219 individuals (95%CI: 9,445–42,062). HBD segments detected by IBDseq similarly showed a very low incidence of RoHs, with the large majority detected in the range of 0.5-1cM, and none individually longer than the 4-8cM range. Both analyses used both transition and transversion sites

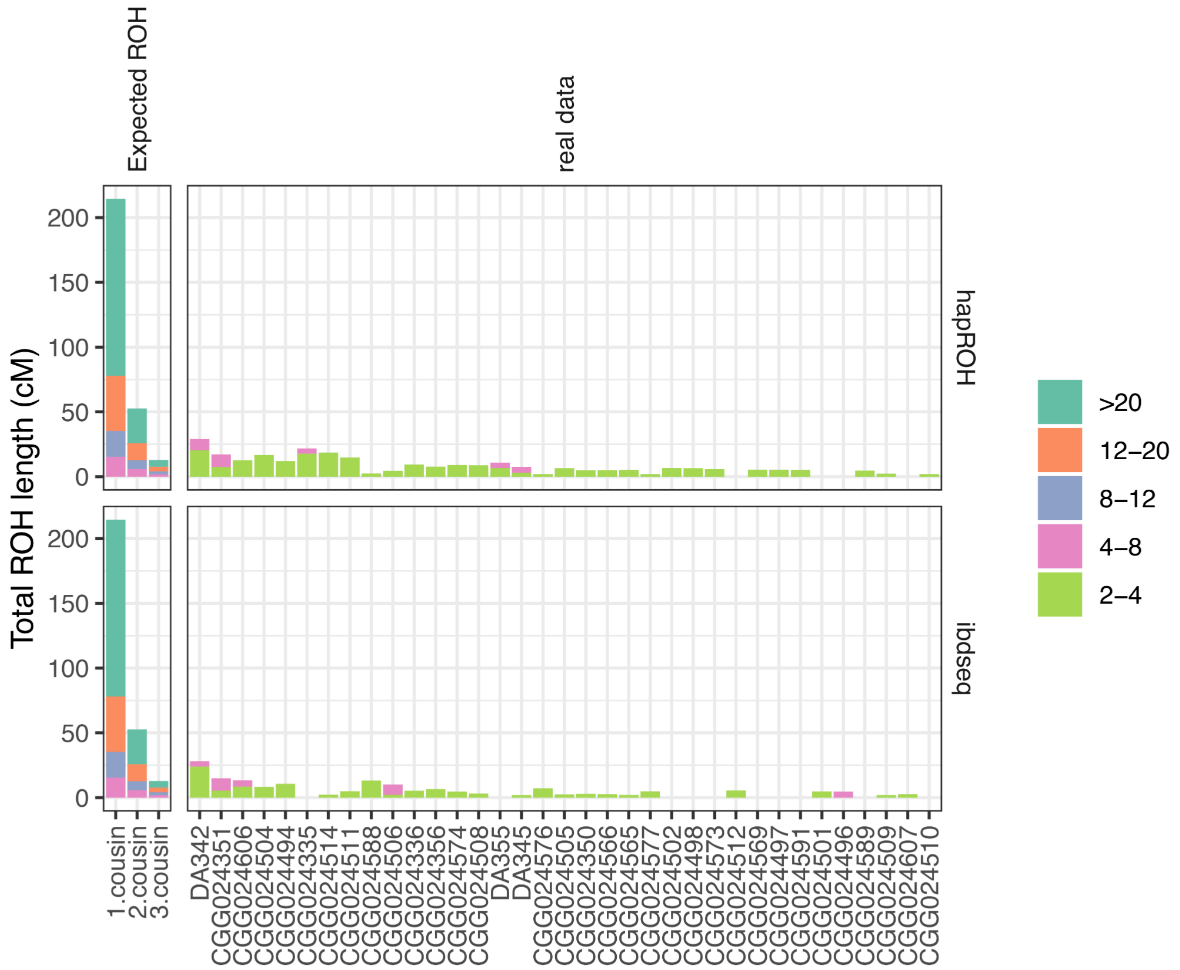

Figure S8: Summed RoHs from Ust'-Ida and Shumilikha cemeteries, generated from hapRoH and IBDseq. No RoHs >2cM were identified from individuals at Bratskii Kamen or Serovo.

<u>Kinship</u>

Pairwise biological kinship was inferred using KIN[21], which was found to have the highest reliability from a range of approaches previously tested in a Baikal hunter–gatherer population (Schulting et al. in prep.). Additionally, this approach is suitable for very low coverage data and was applied at the recommended minimum coverage of 0.05x. As such, the samples from Bratskii Kamen #17 (CGG024570) and Ust'-Ida I #26 (CGG024507) were omitted due to falling below this (0.006x and 0.01x respectively). This method applies a Hidden-Markov-Model approach to infer IBD fragments and estimate relatedness scenarios between pairs of individuals, returning log-likelihood estimates for the two most probable scenarios of relatedness. Pairwise kinship estimates and associated log-likelihoods are provided in Supplementary Data. These were then validated by comparison of KIN kinship

estimates with IBD-sharing results from IBDseq (Figure S9). On the basis of this, one avuncular relationship identified through KIN between Shumilikha #2.01 (CGG024591) and Ust'-Ida #14 (DA355) with shared Y chromosome haplotypes was omitted from the results presented here. Pedigrees were reconstructed assuming that all deaths occurred almost simultaneously, as follows:

Bratskii Kamen Pedigree (light blue): #19.01 (CGG024571) and #19.03 (CGG024573) are identified as third degree related; these share a grave and are inferred to have been buried contemporaneously at the same time (along with #19.02), and likely died contemporaneously; all are young girls. The ages of #19.01 and #19.03 are 8–9 years and 7–9 years respectively; these are therefore inferred as most likely cousins. #19.02 (CGG024572, 4–5 years) has autosomal coverage of 0.001, too low to infer kinship, but all three share the mitochondrial haplotype A+152+16362+16189, with three private mutations. As such, #19.01 and #19.03 are inferred to be maternal cousins, and #19.02 most likely related on their mothers' side.

Ust'-Ida Pedigrees:
(Blue): #8 (CGG024499), #26.01 (CGG024495) and #26.04 (CGG024510) are all identified as most likely full siblings of one another; all are of a similar age and share the mitochondrial haplogroup A10 (Y haplogroup could not be called for #8).

(Green): #6 (CGG024350), 35–50 years, is identified as most likely the father of #33.01 (CGG024505), 12–25 years; they share Y haplotypes.

(Red): #25.01 (CGG024514) and #25.02 (CGG024494) are full siblings (brother and sister) and share mitochondrial haplogroups; these are also 3rd/4th degree related to #6, and #25.01 shares Y haplotypes with him; as well as with #53.02 (CGG024501), 4–6 years, and has an avuncular relationship indicating #53.02 is a nephew on their brother's side.

(Orange): #20.02 (CGG024335), 30–40 years, and #20.01 (CGG024336), 18–24 years; and #20.01 and #33.02 (CGG024502), 13–16 years, both have avuncular relationships, and ages and uniparental haplotypes that indicate they are the nephew and niece on a brother and sister's side respectively. #20.01 and #33.02 are also identified as most likely third degree related (cousins).

(Brown): #18 (CGG0244498), 11–13 years, is the nephew of #56.01 (DA345), 35–50 years, on his father's side, on the basis of their avuncular relationship, haplogroups, and ages. #56.01 is also the grandfather of #44.01 (CGG024504), her father's father as they do not share mitochondrial haplogroups. In turn, #44.01 is the half-sibling of #44.02 (CGG024492) with a shared mother (having the same mitochondrial haplotypes), and she is also third degree related to DA345 (though not inbred).

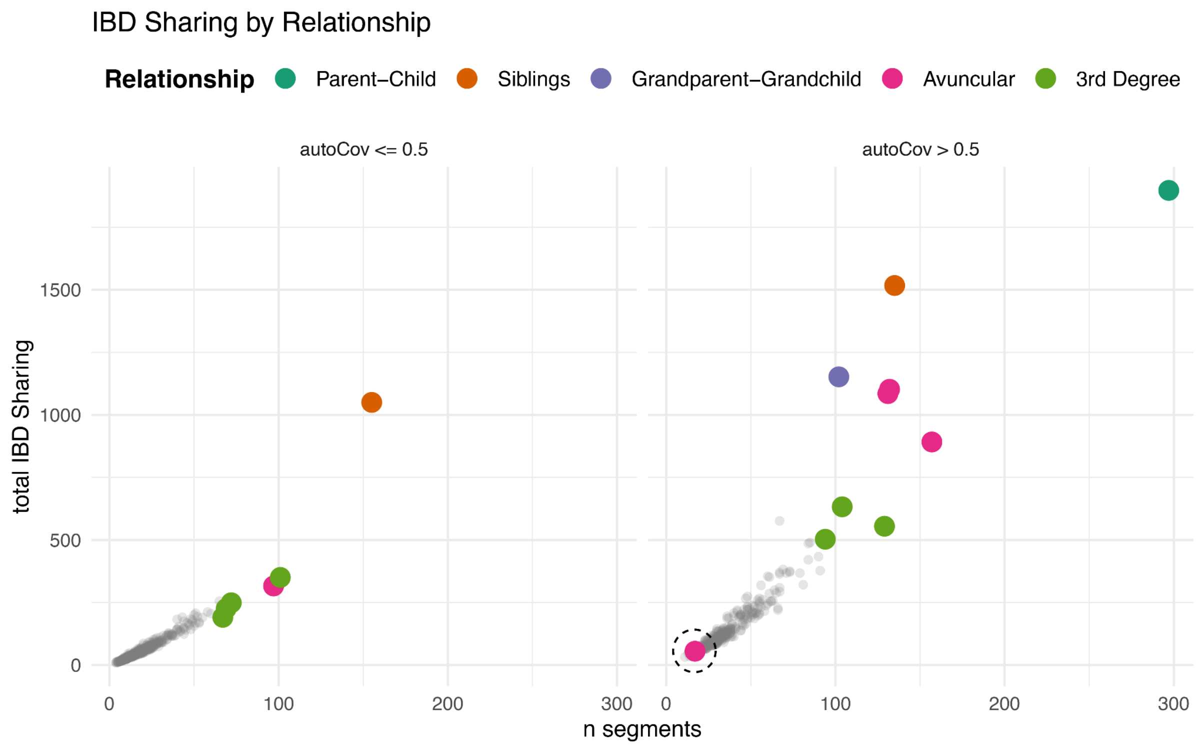

Figure S9: Total IBD-sharing between pairs of individuals in this study. IBD sharing was calculated using IBDseq and each pair is coloured by their relationship as inferred by KIN. One clear outlier is observed where KIN and IBDseq disagrees: the putative avuncular relationship between CGG024591 and DA355 (circled with a stippled line). This relationship was further investigated by the tools KING and ngsRelate which both inferred CGG024591 and DA355 to be unrelated. Accordingly this relationship has been manually changed from ‘avuncular’ to ‘unrelated’ in Supplementary Table 5.

Principal components Analysis

Ancestry of ancient individuals was explored by Principal Components Analysis (PCA) as detailed in the Methods. This showed that all individuals clustered as expected with previously published genomes from Late Neolithic and Early Bronze Age Baikal hunter–gatherers, at the ‘East’ end of the Eastern/Western Hunter–Gatherer ancestry cline (Extended Data Figure 1). An investigation of population affinities was also made using outgroup F3 statistics (Figure S10), which indicated results consistent with previous findings for individuals of this ancestry[12,22].

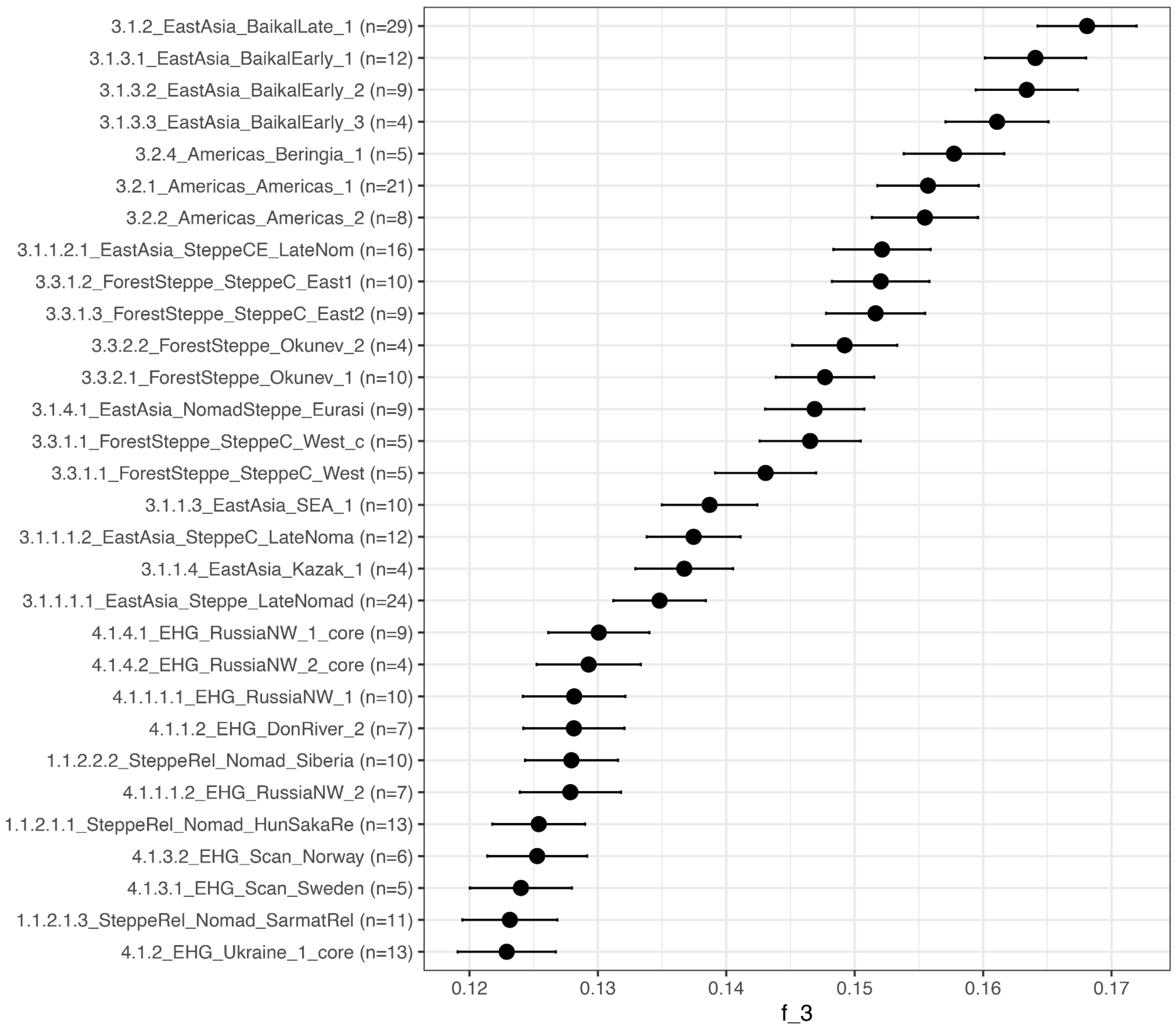

Figure S10. Outgroup F3 statistics of the form $f3$(outgroup; Baikal plague group, listed test group;) comparing our test samples with IBD groups from Allentoft et al. 2024. As the outgroup we used the IBD group '5.1_Africa_SAPast_1'. Only the 20 closest populations to our samples are shown.

Supplementary Note 3: Ancient Pathogen DNA Analysis

Metagenomic detection of Pathogens

Following filtering of pairwise alignment results with the thresholds described in Methods, the most predominant microbial pathogen identified among shotgun-sequenced ancient DNA data was *Yersinia pestis*, with 525,355 uniquely aligned reads across screening data from all samples (see Supplementary Data). Higher coverage plague genomes were utilised for downstream analysis, following merging with genome capture data where this was performed (Table S2). Aside from *Y. pestis* taxonomic identifications, the most frequently identified bacteria are of *Pseudomonas* spp. (from 22 individuals), followed by *Streptococcus spp.* and *Acinetobacter spp.* (from 15 individuals each). In most of these cases, it appears that these can be attributed to environmental strains of these taxa, for example Acinetobacter sp. DUT-2, where reference data were sequenced from marine sediments (https://www.ncbi.nlm.nih.gov/biosample/SAMN04525297). Nonetheless, it is potentially significant that overall, 74% of the identified microbes within these ancient samples are gram-negative. Though many of the non-environmental gram negative bacteria are harmless to humans (e.g. *Wolbachia sp.* and *Brevibacterium flavum*) or are oral symbionts (e.g. *Streptococcus vestibularis*), a number of these are of note as reported human pathogens.

The significance of the identification of *Streptococcus pseudopneumoniae* (identified in Bratskii Kamen #19.01, CGG024571) is also unclear; while it has been suggested this may have a clinical significance in pneumonia infection, this remains under debate[23,24]. It is important to note that pathogenesis is not only a microbial trait but an outcome of the interplay between a host and a microbe. While it is likely that *Y. pestis* caused severe disease in all those infected, the consequences of the presence of many other microbes, especially those sometimes termed opportunistic pathogens, are more challenging to appreciate. Today, we find that while some microbes, e.g. *Streptococcus pseudopneumoniae,* preferentially cause disease in those with weakened immune systems, they can also sometimes cause disease in those with healthy immune systems. Likewise, the immune system's efficiency can be affected by starvation or malnutrition in those not otherwise immunocompromised; this might facilitate serious infections by microbes that otherwise would not have been a likely cause of disease. In some cases, the host's immune response to infection causes the most severe symptoms, e.g., in infectious mononucleosis (primarily caused by Epstein-Barr virus (EBV) infection in teenagers), sepsis, or toxic shock syndrome, typically caused by some gram-positive bacteria or *Y. pseudotuberculosis*). Bacterial superinfections following viral infections or bacterial co-infections further complicate host susceptibility to a severe outcome. Moreover, the pathogenic potential of specific microbes in the past might not fully be reflected in the most similar variants today. Accordingly, the consequences of many of the microbes found in aDNA samples are challenging to appreciate fully.

sampleId	Site (burial number)	Shotgun coverage	merged capture and shotgun coverage
CGG024508	Ust'-Ida I (#31)	0.174	0.659
CGG024510	Ust'-Ida I (#26.0)	0.073	-

CGG024511	Ust'-Ida I (#32)	0.095	-
CGG024571	Bratskii Kamen (#19.01)	0.06	-
CGG024572	Bratskii Kamen (#19.02)	0.03	-
CGG024573	Bratskii Kamen (#19.03)	0.116	-
CGG024574	Bratskii Kamen (#22)	0.742	1.577
CGG024576	Serovo (#10)	0.935	0.976
CGG024588	Ust'-Ida I (#16.01)	0.304	0.733
CGG024606	Shumilikha (#34)	2.619	6.358

Table S1. Coverages for plague genomes with sufficient data to be included in the phylogeny (Fig. 2).

Brucellosis in a Prehistoric Hunter–gatherer

The identification of *Brucella sp.* in Ust'Ida I #26.04 (CGG024495) is notable in that very little genetic data presently exists for this pathogen from prehistoric contexts, particularly from human remains. Bacteria within the genus *Brucella* infect a range of mammalian hosts, and are causative of brucellosis infections which today account for health and economic impacts at a global scale, with up to 12.5 million cases in humans estimated annually[25]. Brucellosis occurs almost exclusively through direct zoonotic transmission from animals to humans, either through contaminated food (commonly unpasteurised milk) or contact with infected animals (extremely rare cases of human-to-human transmission occur mostly in newborn and breastfeeding infants[26]). As such, the identification of brucellosis in an ancient individual is almost certainly directly indicative of zoonotic transmission from an animal to a human. Typically, this transmission is associated with domestic animals, e.g. cattle (*B. abortus*), sheep (*B. melitensis*, *B. ovus*), pigs (*B. suis*), and dogs (*B. canis*). While brucellosis primarily causes fever and systemic infection, skeletal brucellosis is a common complication, and putative identifications from skeletal lesions from the archaeological record, have primarily been found in contexts of close human interaction with livestock domesticates[27]. A significant exception is a possible diagnosis of brucellosis based on lesions in a hominin fossil of *Australopithecus africanus*[28], though this was later interpreted as an anterior disc herniation[29].

Ancient genomic evidence of brucellosis infection has been reported for *Brucella melitensis* from a Neolithic sheep [30] and two humans, both from Medieval contexts: from a 14th Century Friar's kidney stone [31] and a calcified nodule in another 14th Century male [32]. An earlier study also identified the presence of *Brucella sp.* from the amplification of short marker sequences by sampling from the lytic bone lesions of two males from Albania [33].

Metagenomic analysis using the *pathopipe* pipeline[34], identified reads sequenced from CGG024495 as *B. canis*, the causative agent of canine brucellosis, a highly contagious infection in dogs and other canids transmitted through sexual contact or bodily fluids[35]. This identification is based on 96 uniquely aligned reads (covering 3009 bps in total) for this bacterium, passing all the additional filtering thresholds described above. While there are relatively few reads from which to estimate DNA damage patterns, we observe that the

damage associated misincorporation rate at the 3’ end of strands is 5.26%. Given the very small amount of genome coverage available, and high sequence similarity between *B. canis* and other *Brucella* species[36], we felt that a species-level identification could not be reported on the basis of these results alone.

Accordingly, we explored the *Brucella* signal further to establish whether it is possible to get closer to a species assignment with the data at hand. First, we mapped all *Brucella* reads from CGG024495 to the reference genomes of *B. canis* (GCF_000740335[37]). We compared this alignment to alignments of simulated reads generated from all *B. canis* and *B. suis* assemblies in our pathogen screening database to the same reference genome. We visualised all SNPs covered in CGG024495 that were not fixed across all samples (see Figure S11). From this analysis, we could not identify a clear pattern enabling us to assign the CGG024495 sample to either *B. canis* or *B. suis*.

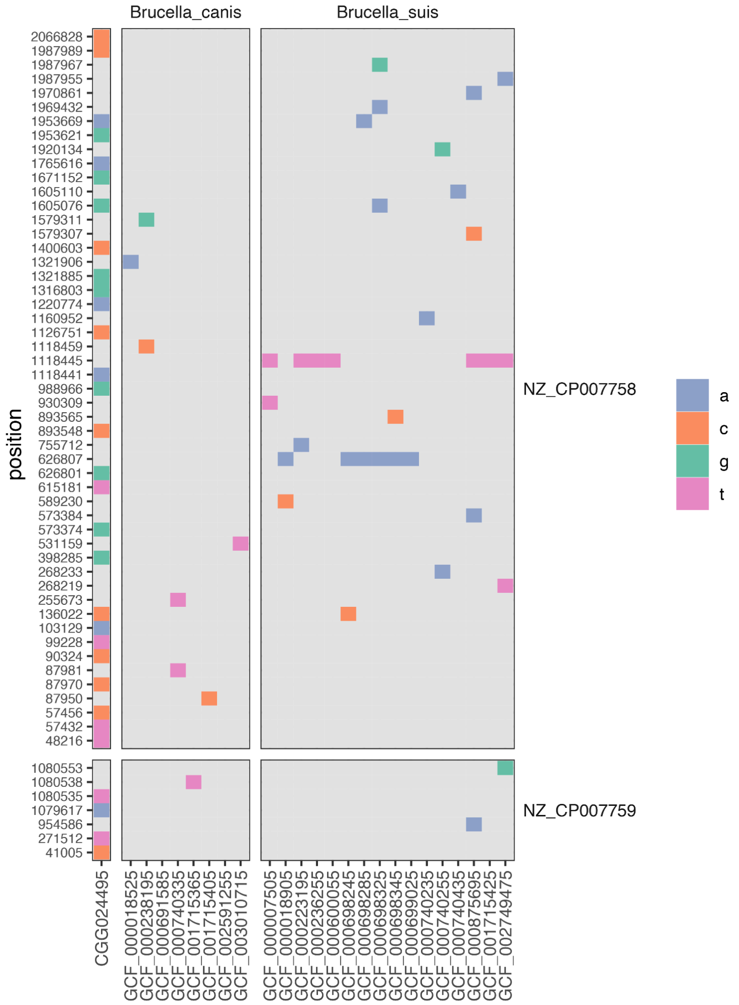

Figure S11. Variant positions of all Brucella reads from CGG024495. *Brucella* sp. reads from CGG024495 mapped to the reference genome of *Brucella canis* (GCF_000740335), compared to alignments of simulated reads generated from select *Brucella canis* and

Brucella suis assemblies. Only variants covered in CGG024495 that were not fixed across all samples are shown.

Furthermore, we blasted all 96 reads from CGG024495 against the *nt* database, and visualised the best hit for each read (Figure S12). This analysis showed the strongest support for *B. melitensis*, which contrasts with the results obtained from our pathopipe workflow. Consequently, from all the additional analyses undertaken, we do not believe that we have sufficient data to make any well supported claims on the specific *Brucella* species identified.

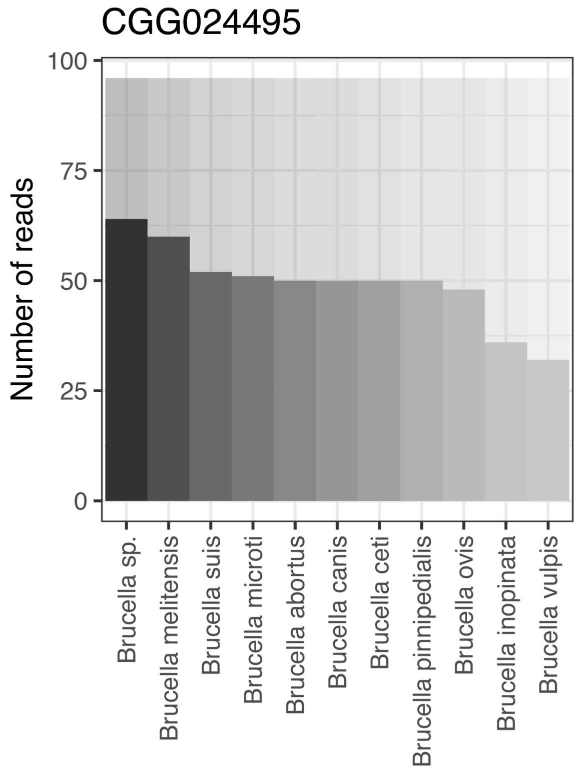

Figure S12: Blast analysis of all (96) *Brucella* reads from CGG024495. Bars represent the total number of reads with a best match to each species. Only the most common species are shown.

Nonetheless, of the principle species of animal hosts known to transmit the disease to humans today (cattle, buffalo, goats, sheep, camel, pigs and dogs[36]), only dogs (and other canids) are known from archaeological assemblages at Lake Baikal at this point in time. Infection can lead to infertility in both sexes, as well as, e.g., anorexia, pain, and lameness. Human infection results in a febrile syndrome characterised by general symptoms such as splenomegaly, fatigue, and weakness[38], and would have been very unlikely to have been the cause of death. The affected individual is a 10–12 years old male, directly dated to 5585–5065 cal. BP, and who is buried alongside his elder sister (positive for plague) in the shared grave #26, as well as having a brother located in the nearby grave #8. This individual was additionally noted as slightly below the threshold number of reads for confident identification of *Y. pestis*.

Dogs are well-attested archaeologically in association with mid-Holocene hunter–gatherers at Lake Baikal, and dog burials in human-like graves occurred in Early

Neolithic *Kitoi* cemetery sites, though no canid remains have been identified as dating to the Late Neolithic period at Baikal so far[39]. The mortuary treatment of dogs during this preceding period nonetheless implies the attachment of significant cultural importance to human relationships with these animals, whether symbolic or practical[40]. Additionally, wolves (*Canis lupus*) are known from burial sites in the Early Neolithic (and even a bear burial at Shamanka II), and although stable isotope analysis has suggested that dogs were eating a similar diet to humans[39], it is unclear as to the extent dogs filled a similarly undomesticated niche to wolves at this point in time or not. Infection could therefore have come equally from either a dog within a commensal or domesticate context of association with humans, or a wild dog or wolf. Consumption of canid remains, or the ritual exposure of bodily fluids (particularly in relation to the sexual organs) might explain this finding, especially in the context of earlier archaeological evidence for ritualised treatment of canid remains. Overall, this result further emphasises the significant role which zoonotic pathogens likely played in the lives of prehistoric hunter–gatherers.

Phylogenetic Modelling and Bayesian Estimation of Node Dates

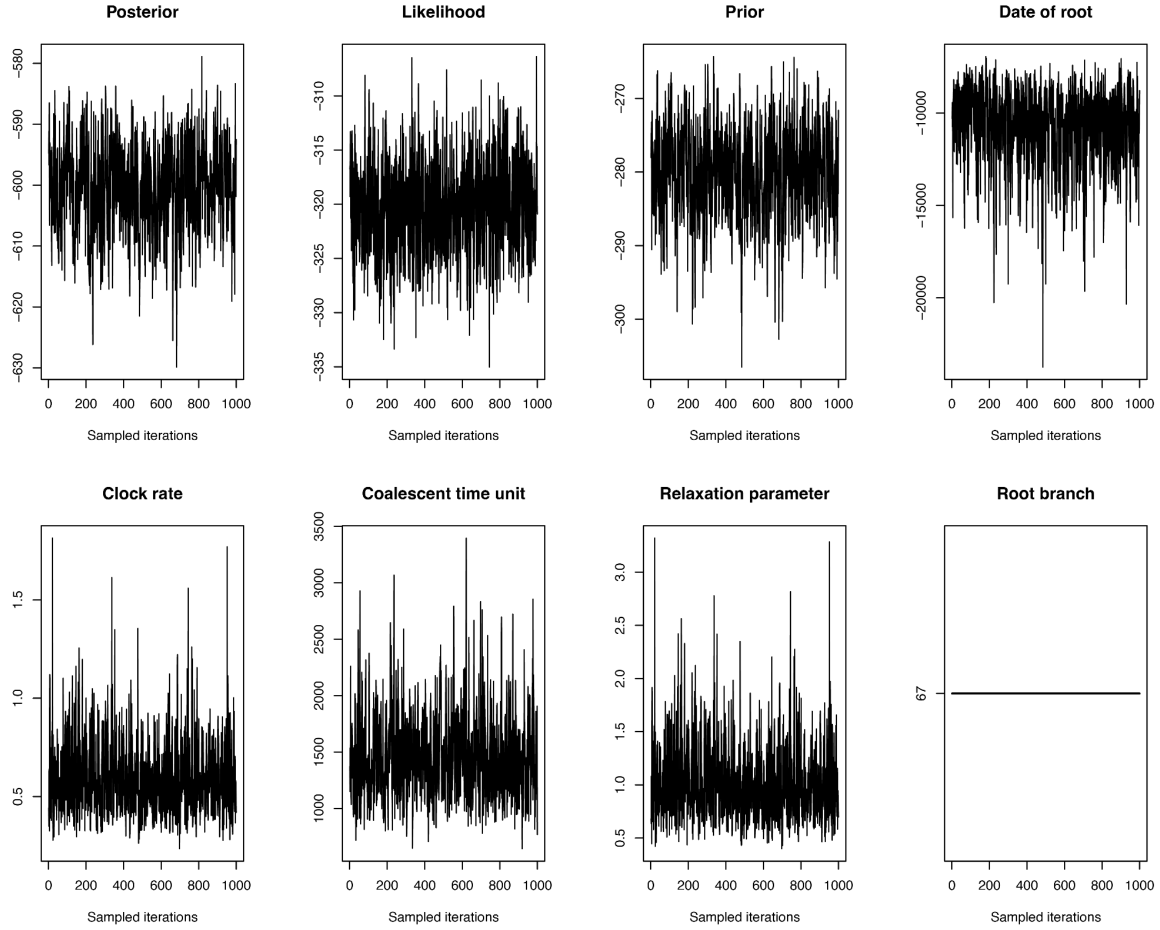

Figure S13: The trace from BactDating after 100,000 iterations. This shows convergence for the estimate of molecular divergence dates. Iterations were sampled every 1000 iterations.

The full phylogenetic tree created with RAxML was converted to a json file for the USHER tree annotated with substitution information - this can be viewed interactively through this github repository: https://github.com/ramacleod/Prehistoric_plague_MAT.

For the phylogenetic tree constructed using RAXML, we investigated the effects of differing read-depth cut-off used for calling genotypes, testing this for minimum read depths of two and four as well as three (Extended Data Figure 3). From this analysis, we found that the tree topology is not affected by the read cut-off. However, as expected, we do observe a higher rate of private mutations when the filter is very low (cutoff: 2). Based on this comparison, we conclude that it is unlikely that the current cut-off could have had an effect on divergence estimates.Our investigation of recombination within the subset phylogeny used for the Gubbins analysis shows a generally low incidence of recombination, with slightly more visible in the *Y. pseudotuberculosis* part of the phylogeny (Figure S14).

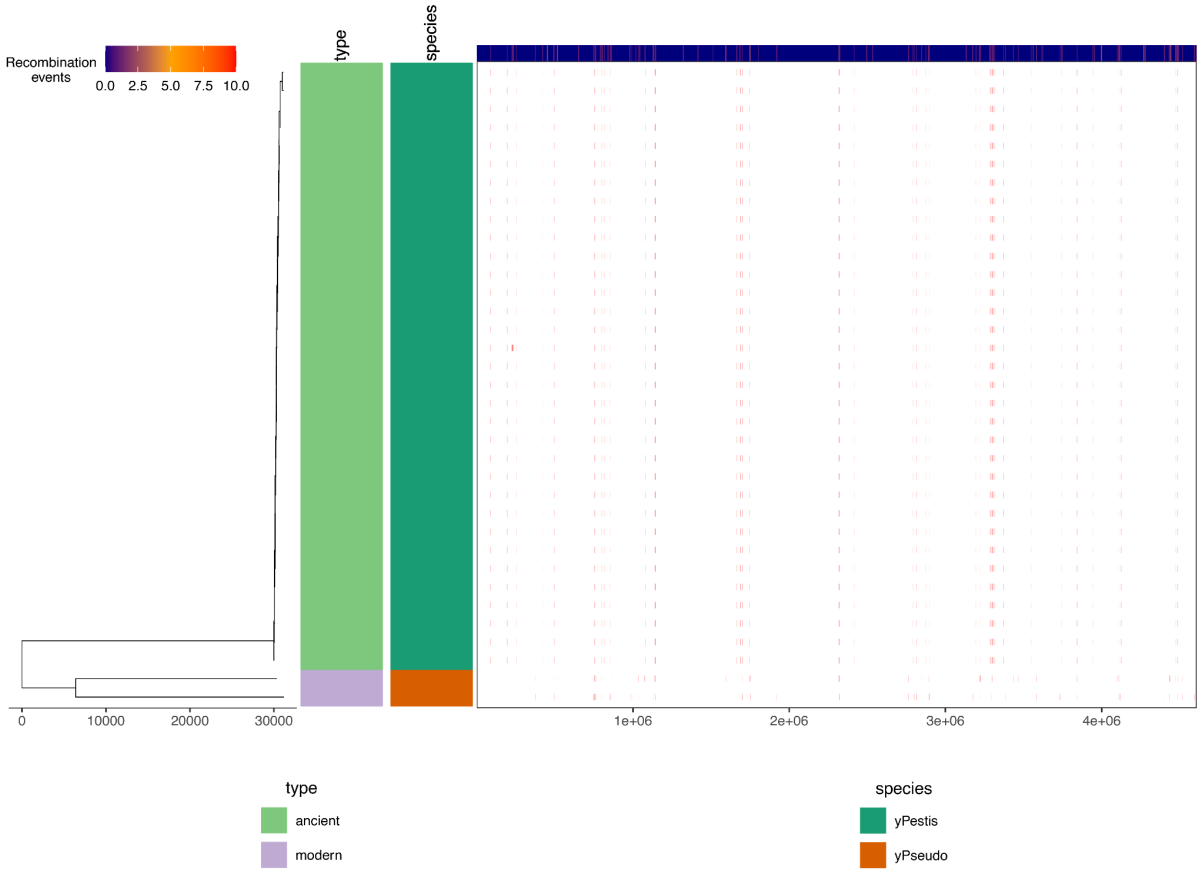

Figure S14: Recombination events within Y.pestis and Y.pseudotuberculosis. Left hand side: Phylogenetic tree built on non-recombinant regions of each genome. Right hand side: Heatmap of recombination events within each genome.

Investigation of variant position within the high coverage Lake Baikal genomes

We identify a total of 21 variant positions between Shumilikha #34 (CGG024606), Serovo #10 (CGG024576) and Bratskii Kamen #22 (CGG024574). Of these, the vast majority (14 positions) represents variants that differ between the two higher coverage genomes (Shumilikha #34 and Bratskii Kamen #22) with no data from Serovo #10. At three variant positions, we have data from all three genomes. However no clear pattern can be observed from these positions, as they represent one private mutate each, from each of the three genomes. Still, in agreement with the phylogenetic tree (Figure 2), we find that the two north cluster genomes are genetically more similar (2 variants out of 1,115 shared SNP positions), whereas, the earlier south cluster genome appear more distantly related to the two later ones (6/2,269 and 16/4,670 variants; Figure S15)

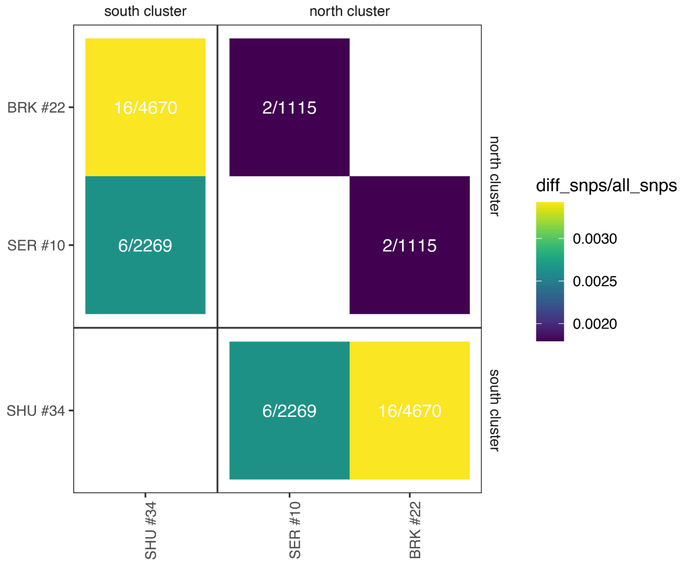

.

Figure S15: Variant positions between the three higher coverage Lake Baikal genomes. Number of called SNPs differing between any given combination of two samples normalised to the total number of shared SNPs between those samples.

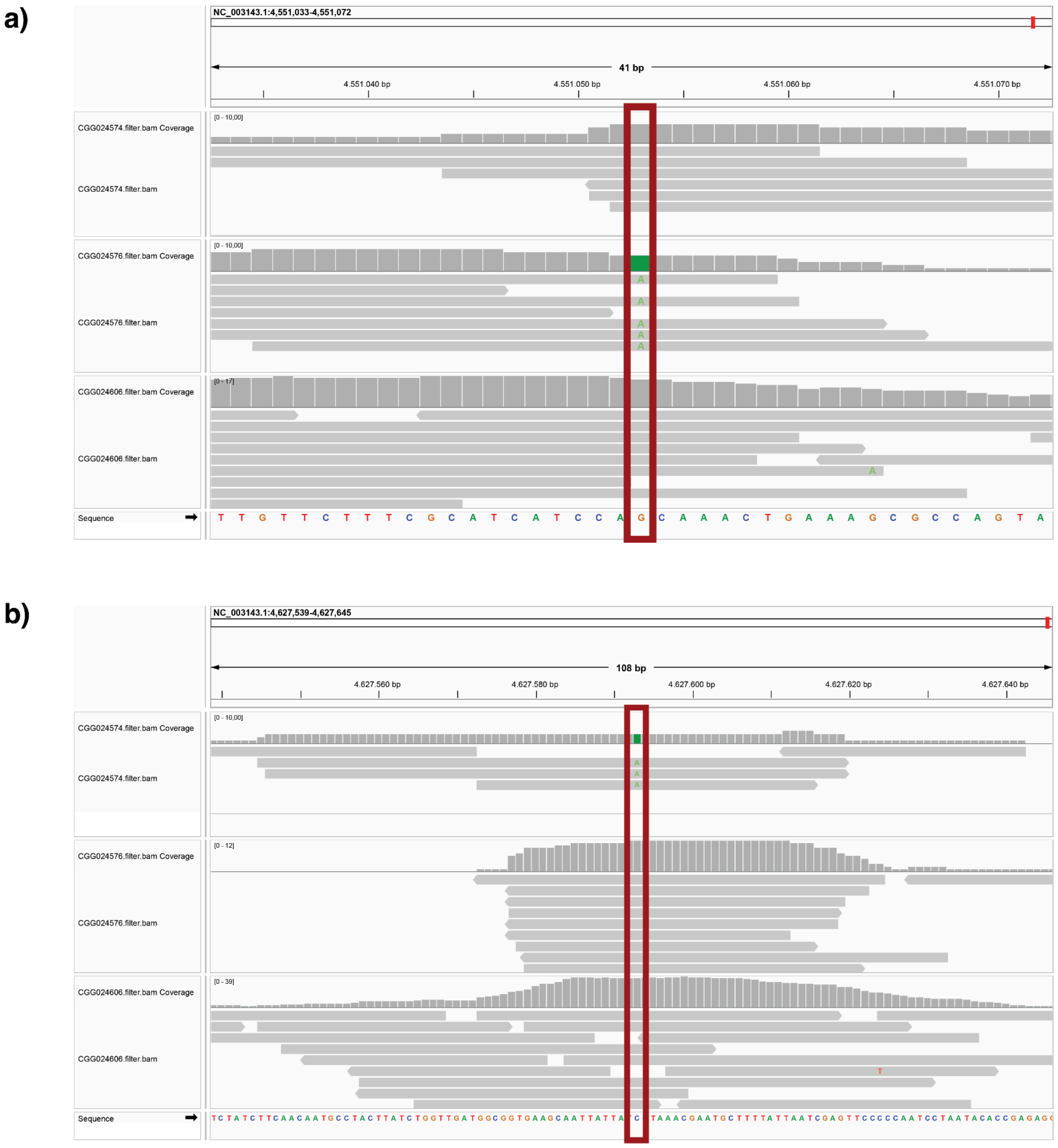

Figure S16: Positions differing between between BRK #22 and SER #10. Red bar indicates the variant position. Visualised using IGV.

Investigation of YPM variants

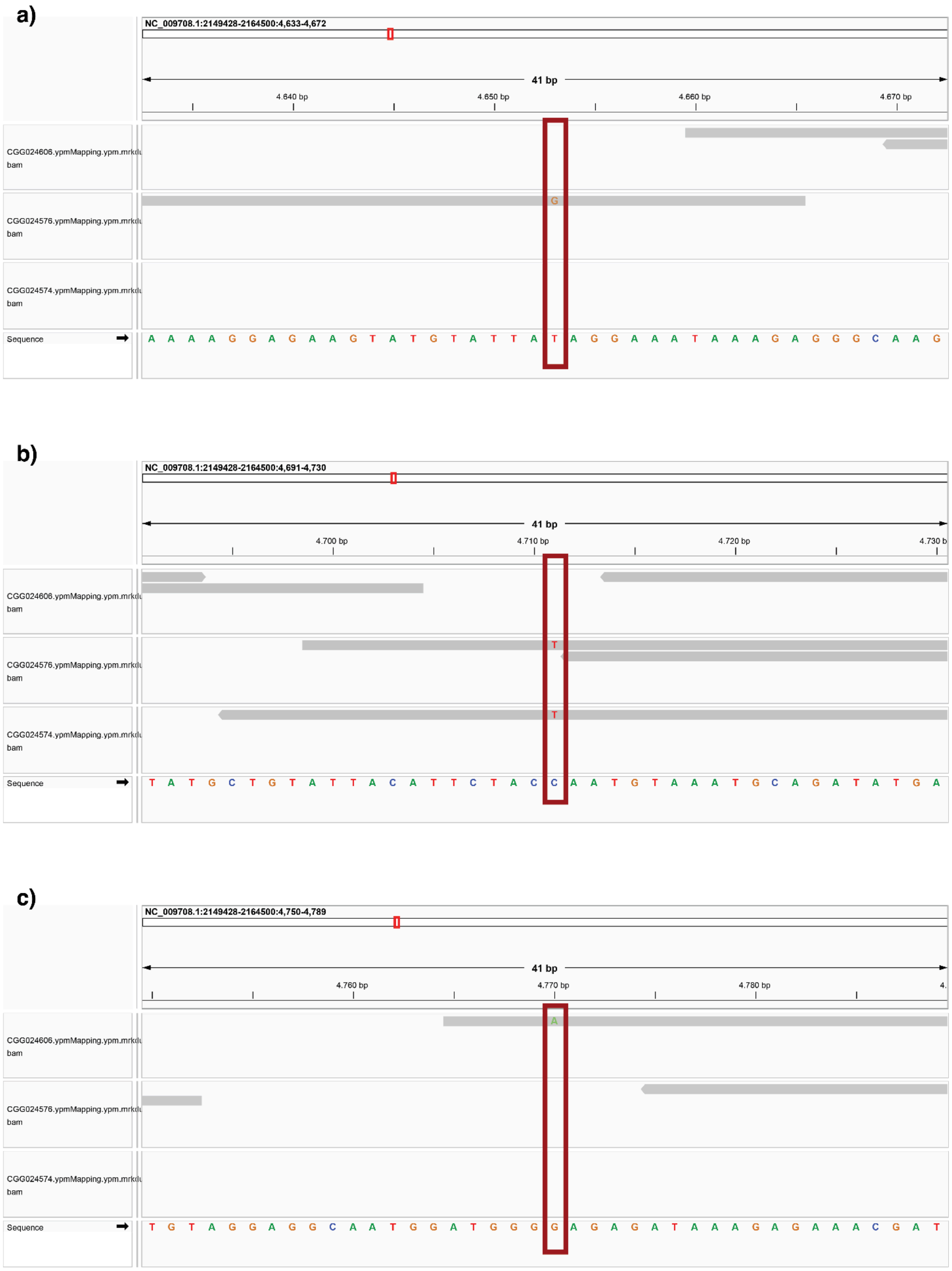

Figure S17: IGV visualisation of the three YPM variants identified. For all high coverage samples in this study - Shumilikha #34 (CGG024606), Serovo #10 (CGG024576) and Bratskii Kamen #22 (CGG024574) - all three variants within the YPM gene (positions 4653, 4711 and 4779), were visualised. Red bar indicates the variant position. Visualised using IGV.

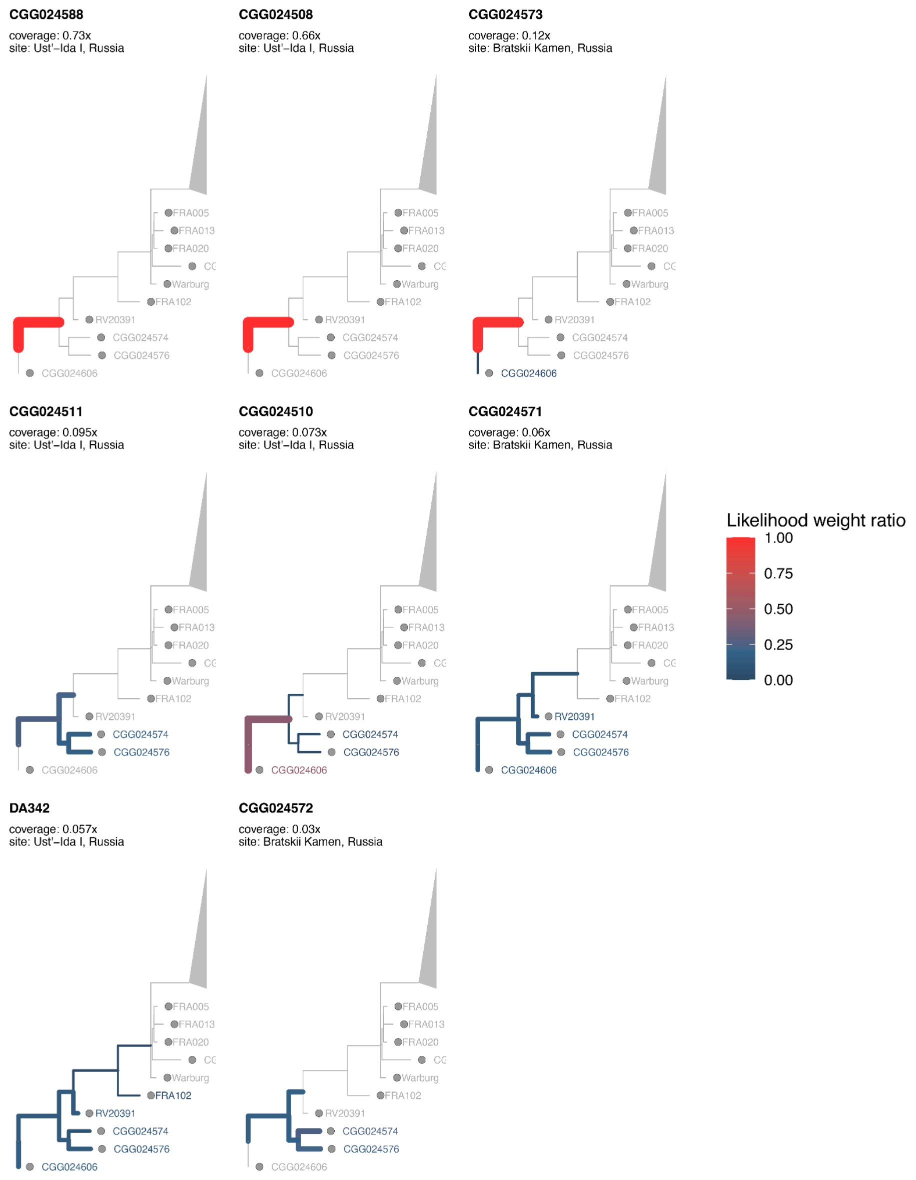

Figure S18: EPA-ng placement of lower coverage plague detections from Lake Baikal. Each subplot depicts the placement of one lower coverage sample onto the plague phylogeny from Figure 2. Branch width and color indicate the likelihood weight ratio of the placement.

<u>Permutation Test for Plague Presence Among Close Relatives</u>

In order to test whether there is a significant pattern of higher plague prevalence within families, we ran a permutation test. In our dataset, we observe a total of six related pairs of individuals where both have plague. To test whether this pattern would be expected if the plague was randomly distributed among all individuals, we ran a permutation test. We reshuffled plague presence across the dataset 10,000 times and recalculated the total number of double plague positive pairs for each permutation. From this test, we found that the number of double plague positive pairs was higher than or equal to the observed data in 1,064 permutations (p-value: 0.1). Accordingly, we conclude that the co-occurence of plague within families is not statistically significant.

Supplementary Note 4: Radiocarbon Chronology

Radiocarbon dating was undertaken through direct sampling of skeletal elements (principally either cranial, femoral or costal bone, see Supplementary Data). All subsampled material was processed at the Oxford Radiocarbon Accelerator Unit (ORAU) at the University of Oxford, UK. Carbon isotope measurements were made on ultra-filtered collagen following Brock et al.[41]. Additionally, collagen quality was assessed following the criteria of DeNiro[42] and van Klinken[43], with none of the reported samples rejected on the basis of these. Radiocarbon dates were calibrated in OxCal v4.4[44] using atmospheric data from the IntCal20 calibration curve[45]. A Kernel Density Estimate function ("KDE_Plot")[46] was used to display radiocarbon date ranges in OxCal.

A total of 70 radiocarbon dates were obtained and considered from the LN burials of the four studied sites. Of these, 27 radiocarbon dates are reported here for the first time; these comprise 10 from Late Neolithic individuals from Bratskii Kamen, 6 from Serovo, and 6 from Shumilikha. Raw dates, calibrated date ranges and metadata are provided for these samples, alongside the same for the 36 Late Neolithic individuals from Ust-'Ida I reported by Weber et al.[31], Weber et al.[47], Bronk Ramsey et al.[8]. To mitigate the Freshwater Reservoir Effect (FRE) resulting from human consumption of aquatic resources and metabolisation of older carbon, a correction was applied using the equation below, after Weber et al.[47], based on the regression model developed by Schulting et al.[48].

$$Y = -1388.8522 + 125.4503 \times \delta^{15}\mathrm{N}$$

Equation 1. Regression equation for the correction of radiocarbon dates from human skeletal tissue in mitigation of the Freshwater Reservoir Effect in the Angara Valley region of Cis-Baikal.

Bayesian modelling of the radiocarbon dates was undertaken in OxCal 4.4 (Bronk Ramsey 2009; 2024), using non-informative, single-phase models with uniform boundaries. Model results are rounded to the nearest half-decade (as they will vary stochastically from run to run) and presented in italics. To visualise summed multiple dates, kernel density estimation (KDE) models and plots within Bayesian models were employed (Bronk Ramsey 2017).

Eight radiocarbon dates are on red deer (*Cervus elaphus*) tooth pendants associated with specific individuals as grave inclusions. They are used in place of the human dates for these individuals, as they are more precise since they avoid the added uncertainties imparted by the freshwater reservoir correction. Also included are a small number of children aged 4–5 whose stable nitrogen isotope (δ^{15}N) values do not differ significantly from the adult mean at their respective sites, and hence are taken to be of post-weaning age (relevant for the calculation of a freshwater reservoir correction to their radiocarbon date, on which basis they were previously excluded).

In cases where multiple dates are available for the same individual, they were combined using the R_combine function in OxCal. If the duplicates were made on the same collagen preparation (as part of the Oxford Radiocarbon Accelerator Unit's internal quality control), they were combined prior to adjustment for the freshwater reservoir effect (FRE) (i.e., Method 1 in Weber et al. 2016). If the dates were done using different bone samples from the same individual, they were first FRE-corrected and then combined (Method 2 in Weber et al. 2016). This is to take into account the possibility that different skeletal elements

experience variable collagen turnover, and so may represent different times of life with potentially different uptake of fish, etc. Only a small number of individuals have multiple dates.

Individuals testing positive for the plague clearly fall into an early and a late group (Figure S19). All the individuals with plague from Ust'-Ida I can be successfully R_Combined and hence are not inconsistent with a single event (Ward and Wilson 1978). Three dates on infected individuals from Bratskii Kamen can be combined but the fourth (BRK_1956.022) is significantly later. Shumilikha (SHU_1972.034) and Serovo (SER_1957.010) are each represented only by a single case. All the Ust'-Ida I cases together with Shumilikha and the three early cases from Bratskii Kamen can be combined into an early phase (4663 ± 18 BP (χ^2, df=15, T=18.1 (5% 25.0)) (Figure S20). The more recent date from Bratskii Kamen and the single case from Serovo can be combined into a late phase (χ^2, df=15, T=1.3 (5% 3.8)) (Figure S21).

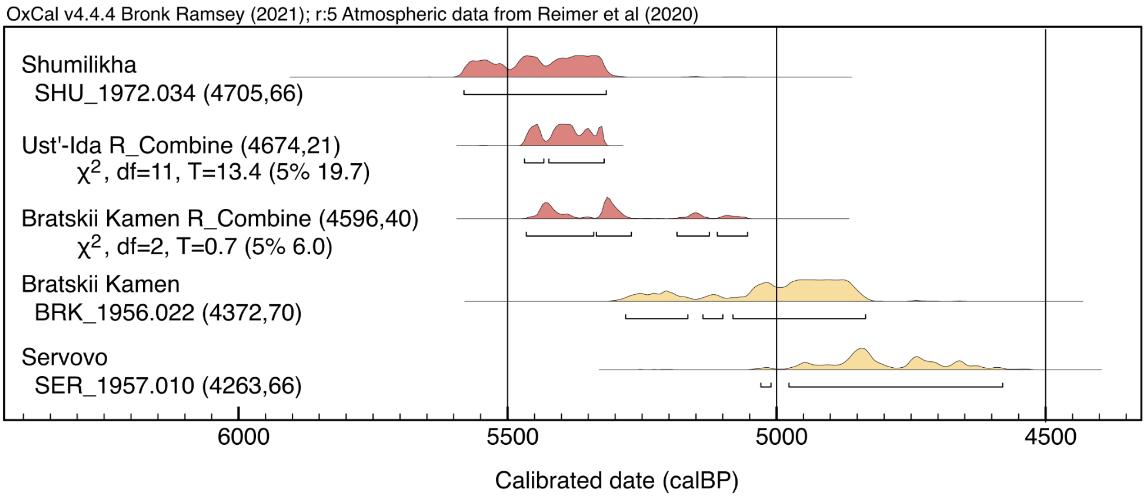

Figure S19. Site-based groupings for all LN individuals positive for plague. Early phase is indicated in red, later phase in yellow.

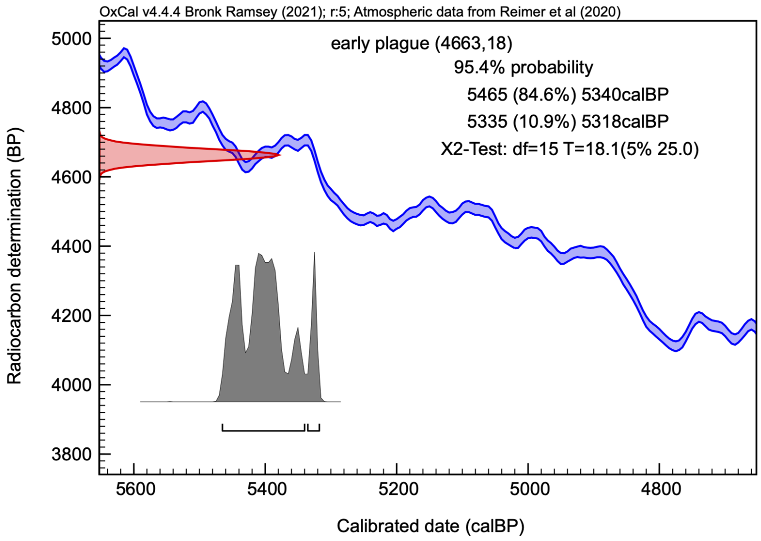

Figure S20. Combined dates for early phase plague victims (n = 16)

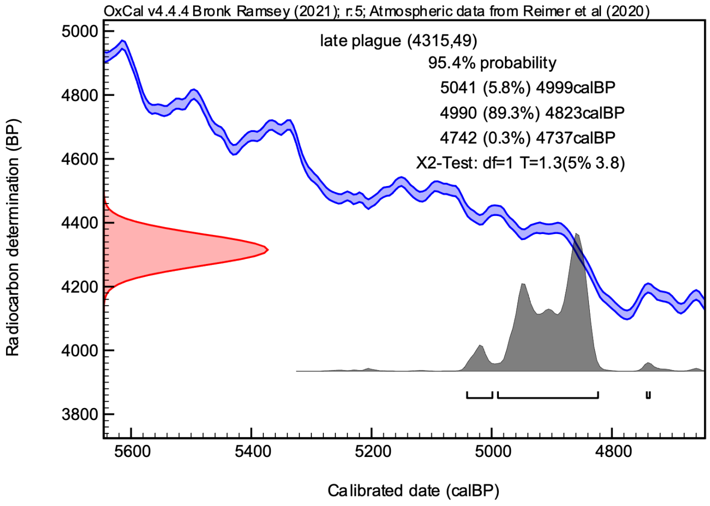

Figure S21. Combined dates for late phase plague victims (n = 2)

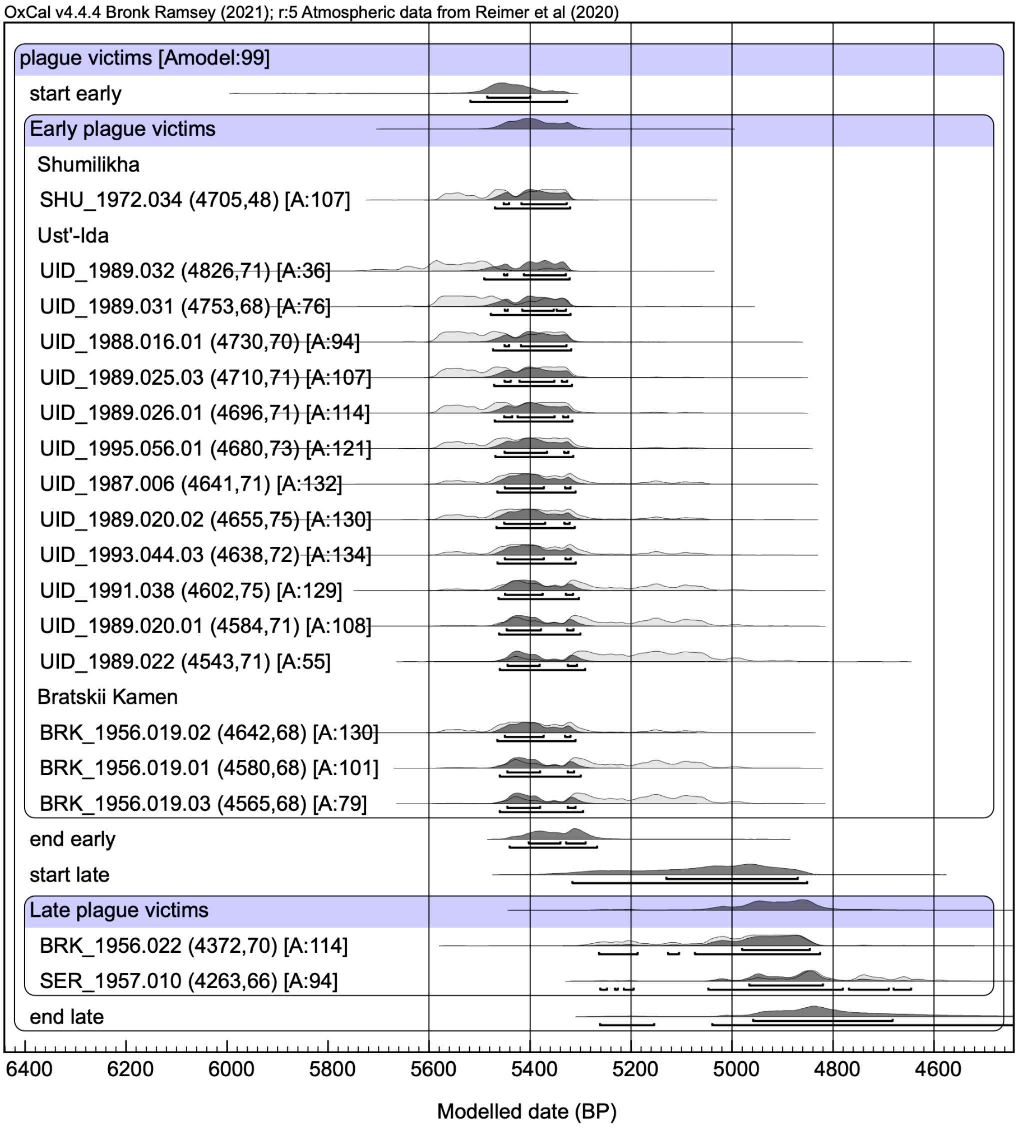

Figure S22. Bayesian model for all Late Neolithic individuals with plague, divided into 'early' and 'late' phases.

Bayesian modelling offers another approach to the chronology of the plague victims. Given the above discussion, two phases are modelled, using uniform boundaries. This strongly reinforces the impression of two short-lived mortality events (Figures S22 and S23). This is particularly convincing for the early phase, with a larger number of dates providing more precise start and end boundaries. The estimated span for the early phase plague victims is *0–102 years* (68.3%) or *0-195 years* (95.4%), with the highest probability being in the order of a few decades (Figure S24). This is not unexpected given the range of ages of the victims (i.e., from young children to mid-older adults).

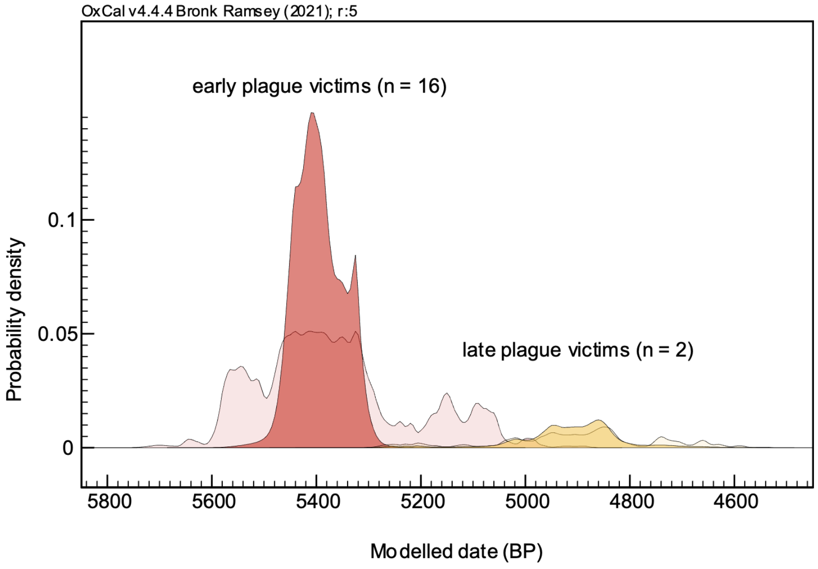

Figure S23. KDE plots within Bayesian models for the early and late plague victims. The lightly shaded areas show the summed probability distributions prior to modelling; red indicates the earlier plague outbreak phase, whilst yellow indicates the later phase.

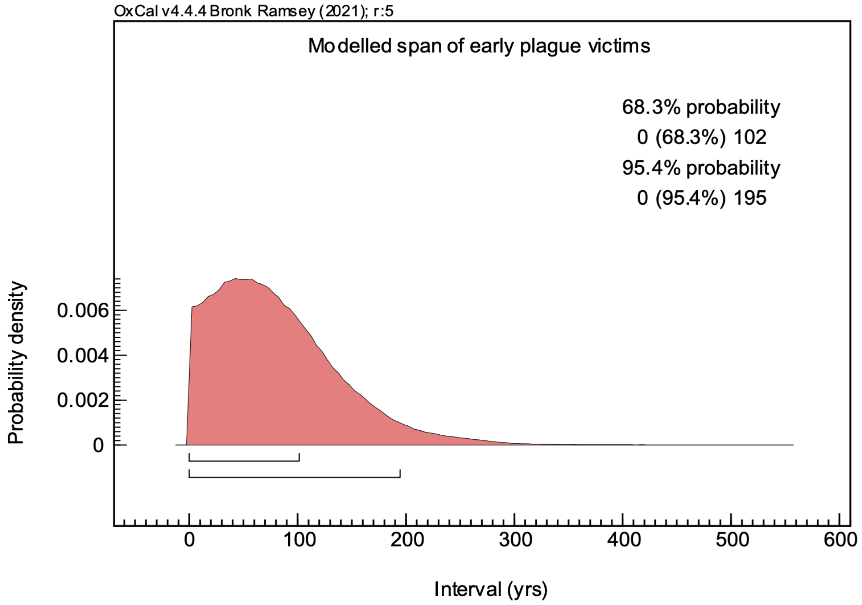

Figure S24. Modelled span of early plague victims.

Some centuries separate the early and late phases of plague. The second plague outbreak is represented by only two individuals (testing positive). Whether this speaks to the scale of the two outbreaks is unclear, since Ust'-Ida I – with the majority of cases – may be a very short-lived plague cemetery and so biasing the sample towards the earlier phase.

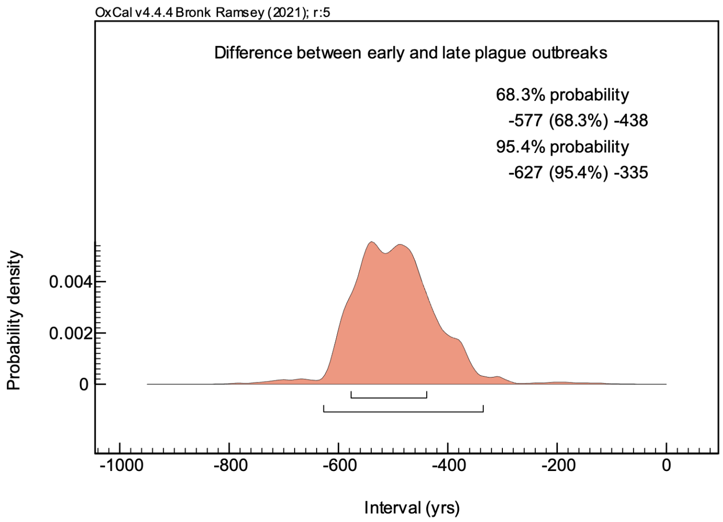

Figure S25. Modelled difference between the early and late plague phases (using the 'Difference' function in OxCal 4.4).

Using the combined dates heuristically (Figures S20 and S21), the early and late phases events are separated by *440–575 years* (68.3%) or *335–625 years* (95.4%) (Figure S25). It should be noted that the Combine function is not strictly speaking intended to be used in this way; rather, it is used to combine multiple dates on the same entity. Nevertheless, it provides a useful method for data exploration.

Another way to approach this is to estimate the difference between the last date in the first phase (BRK_1956.019.03) and the first date in the second phase (BRK_1956.022), both from Bratskii Kamen. This is more realistic than using the phase boundaries, since those for the late phase are poorly resolved given that it contains only two dates. The difference between the two dates is modelled as *390–560 years* (68.3%) or *190–610 years* (95.4%) (Figure S26). The two estimates are not that dissimilar. Thus, it can be proposed that between four and six centuries likely separated the two outbreaks.

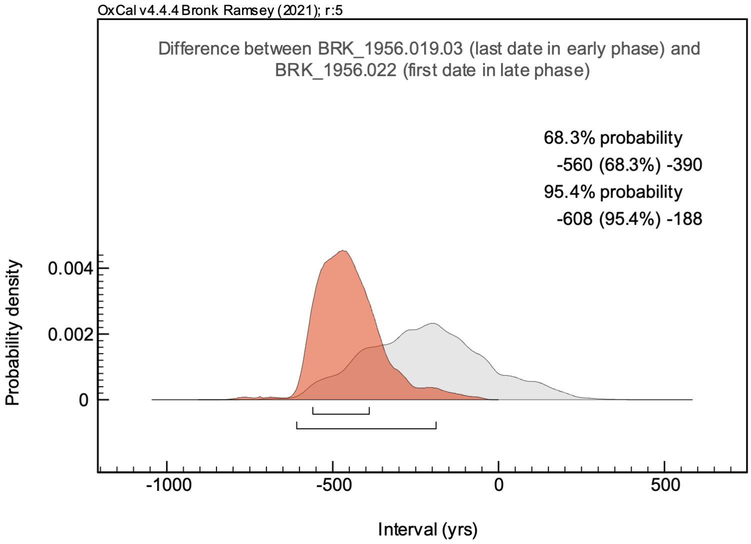

Figure S26. Modelled difference between the last date in the early phase and the first date in the late phase of plague. The light gray shaded region shows the unmodelled probability distribution.

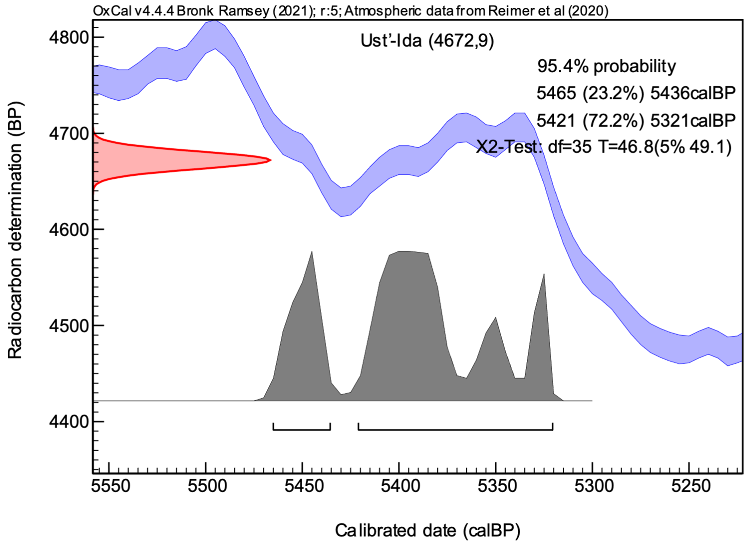

Figure S27. Combined dates for Ust'-Ida I (n = 36). This excludes three burials (#33.01, #56.02 and #44.02) identified in a Bayesian model as having low agreement indices.

Given that many of the graves at Ust'-Ida I in particular contained multiple individuals, and that in a number of cases at least one individual tested positive for plague, it is highly probable that other individuals that did not test positive may have nevertheless died of the plague at or near the same time. The individual skeletons in the multiple graves appear to have been undisturbed, which argues strongly against successive burial. In addition, the radiocarbon dates for the individuals in graves containing at least one plague victim can all be successfully combined in OxCal. In fact, this can be extended to 36 of the 39 directly dated LN individuals in the entire cemetery (Figure S27), representing 75% of its total 48 burials. As those not directly dated are in multiple graves with other individuals that have been dated, this effectively means that the dates for most, if not all (see below), of the individuals interred at Ust'-Ida I are not inconsistent with a single event (χ^2, df=35, T=46.8

(5% 49.1)), though a longer period of some decades to up to two centuries cannot be excluded on the basis of the radiocarbon dates alone. Much of this uncertainty is due to a small plateau in the radiocarbon calibration curve between ca. 5440 and 5330 cal BP.

Moreover, the possibility of a period of recurrent plague deaths over decades at Ust'-Ida I can be excluded by considering the numerous kinship relations at the site. Most importantly, it can be noted that the three individuals from Ust'-Ida I (#33.01, #56.02 and #44.02) with agreement indices less than the recommended 60% (Extended Data Figure 4) (Bronk Ramsey 2009) are either from the same grave as another individual with a good agreement index (Burials #56.01 and #56.02), or closely related to another individual with a good agreement index (Burial #6 is father to #33.01, and Burial #44.02 is a third degree relation of #56.01). In other words, these individuals are not early or late outliers as might otherwise be implied by their modelled radiocarbon dates.

OxCal code. Bayesian model for those individuals testing positive for plague, modelled as two phases (included lat and long at site level for moving visualisation)

```
Plot("Angara plague victims")
 {
  Sequence("plague victims")
  {
   Boundary("start early");
   KDE_Plot("EPV", )
   {
    Label("Shumilikha");
    R_Date("SHU_1972.034", 4705, 48)
    {
     latitude=52.9025;
     longitude=103.6525;
    };
    Label("Ust'-Ida");
    R_Date("UID_1989.032", 4826, 71)
    {
     latitude=53.182078;
     longitude=103.377079;
    };
    R_Date("UID_1989.031", 4753, 68)
    {
     latitude=53.182078;
     longitude=103.377079;
    };
    R_Date("UID_1988.016.01", 4730, 70)
    {
     latitude=53.182078;
     longitude=103.377079;
    };
    R_Date("UID_1989.025.03", 4710, 71)
    {
     latitude=53.182078;
     longitude=103.377079;
    };
    R_Date("UID_1989.026.01", 4696, 71)
    {
     latitude=53.182078;
     longitude=103.377079;
    };
    R_Date("UID_1995.056.01", 4680, 73)
    {
     latitude=53.182078;
     longitude=103.377079;
    };
    R_Date("UID_1987.006", 4641, 71)
    {
     latitude=53.182078;
     longitude=103.377079;
    };
    R_Date("UID_1989.020.02", 4655, 75)
    {
     latitude=53.182078;
```

```
  longitude=103.377079;
 };
 R_Date("UID_1993.044.03", 4638, 72)
 {
  latitude=53.182078;
  longitude=103.377079;
 };
 R_Date("UID_1991.038", 4602, 75)
 {
  latitude=53.182078;
  longitude=103.377079;
 };
 R_Date("UID_1989.020.01", 4584, 71)
 {
  latitude=53.182078;
  longitude=103.377079;
 };
 R_Date("UID_1989.022", 4543, 71)
 {
  latitude=53.182078;
  longitude=103.377079;
 };
 Label("Bratskii Kamen");
 R_Date("BRK_1956.019.02", 4642, 68)
 {
  latitude=56.122222;
  longitude=101.675;
 };
 R_Date("BRK_1956.019.01", 4580, 68)
 {
  latitude=56.122222;
  longitude=101.675;
 };
 R_Date("BRK_1956.019.03", 4565, 68)
 {
  latitude=56.122222;
  longitude=101.675;
 };
 First("First EPV");
 Last("Last EPV");
 Difference("", "end early", "start early");
 Difference("", "Last EPV", "First EPV");
 Span("early plague victims");
};
Boundary("end early");
Boundary("start late");
KDE_Plot("LPV", )
{
 R_Date("BRK_1956.022", 4372, 70)
 {
  latitude=56.122222;
  longitude=101.675;
 };
 R_Date("SER_1957.010", 4263, 66)
 {
  latitude=54.509722;
```

```
    longitude=103.222222;
   };
  };
  Boundary("end late");
  Difference("", "end early", "start late");
  Difference("", "BRK_1956.019.03", "BRK_1956.022");
 };
};
```

OxCal code for KDE models, including all post-weaning radiocarbon-dated humans (with FRE corrections), and on red deer tooth pendants where available.

```
Plot()
 {
  KDE_Model("Ust'-Ida", )
  {
   R_Date("UID_1990.033.01", 4901, 71);
   R_Date("UID_1995.056.02", 4876, 73);
   R_Date("UID_1989.032", 4826, 71);
   R_Date("UID_1988.014", 4774, 71);
   R_Date("UID_1988.017", 4762, 30);
   R_Date("UID_1994.055.02", 4754, 72);
   R_Date("UID_1989.031", 4753, 68);
   R_Date("UID_1989.030", 4750, 72);
   R_Date("UID_1987.010", 4743, 71);
   R_Date("UID_1990.033.02", 4741, 72);
   R_Date("UID_1988.016.01", 4730, 70);
   R_Date("UID_1987.009", 4719, 71);
   R_Date("UID_1994.053.02", 4713, 73);
   R_Date("UID_1988.018", 4706, 30);
   R_Date("UID_1989.025.03", 4710, 71);
   R_Date("UID_1987.011", 4701, 70);
   R_Date("UID_1989.026.01", 4696, 71);
   R_Date("UID_1995.056.01", 4680, 73);
   R_Date("UID_1989.025.02", 4676, 71);
   R_Date("UID_1987.008", 4666, 72);
   R_Date("UID_1991.041", 4695, 32);
   R_Date("UID_1989.020.02", 4655, 75);
   R_Date("UID_1989.026.04", 4646, 72);
   R_Date("UID_1989.026.05", 4646, 52);
   R_Date("UID_1994.052", 4658, 31);
   R_Date("UID_1994.053.01", 4644, 31);
   R_Date("UID_1987.006", 4641, 71);
   R_Date("UID_1993.044.03", 4638, 72);
   R_Date("UID_1993.044.01", 4633, 71);
   R_Date("UID_1994.054", 4617, 74);
   R_Date("UID_1989.021.01", 4611, 30);
   R_Date("UID_1989.020.01", 4608, 30);
   R_Date("UID_1991.038", 4602, 75);
   R_Date("UID_1989.021.02", 4597, 70);
   R_Date("UID_1987.005", 4596, 73);
   R_Date("UID_1988.015", 4585, 71);
   R_Date("UID_1988.016.02", 4572, 71);
   R_Date("UID_1989.022", 4543, 71);
   R_Date("UID_1993.044.02", 4530, 71);
  };
  KDE_Model("Shumilikha", )
```

```
 {
  R_DATE("SHU_1972.044",4838,68);
  R_DATE("SHU_1972.021",4733,67);
  R_DATE("SHU_1972.034",4705,48);
  R_Date("SHU_1972.002.01", 4628, 24);
  R_DATE("SHU_1972.002.04",4591,67);
 };
 KDE_Model("Bratskii Kamen", )
 {
  R_DATE("BRK_1956.021",4734,68);
  R_Date("BRK_1956.010", 4683, 68);
  R_DATE("BRK_1956.017",4623,68);
  R_DATE("BRK_1956.019.01",4580,68);
  R_Date("BRK_1956.019.02", 4642, 68);
  R_DATE("BRK_1956.016",4586,68);
  R_DATE("BRK_1956.019.03",4565,68);
  R_DATE("BRK_1956.011",4443,68);
  R_DATE("BRK_1956.022",4372,70);
  R_DATE("BRK_1956.012",4287,68);
 };
 KDE_Model("Serovo", )
 {
  R_DATE("SER_1957.008",4662,68);
  R_DATE("SER_1957.016",4550,69);
  R_DATE("SER_1957.012",4446,68);
  R_DATE("SER_1957.020",4386,67);
  R_DATE("SER_1957.010",4263,66);
 };
};
```

Supplementary Note 5: Mortality Profile Modelling

Analysis of Age-at-death Profiles

We tested two hypotheses. Firstly that plague phases had a lower than expected average age-at-death. Secondly, that plague phases had an unusually high proportion of juvenile deaths (8-12 yrs old), given the assumption that this specific age range was more susceptible.

Stage 1 requires building a null model for the continuous probability of death at any age. We excluded four individuals who died unborn in-utero. Four further individuals were reported with the false precision of a point age (p) rather than a range. These were given a range of 0.8p to 1.2(1/6 +p) where p was measured in years. This resulted in a dataset of 625 individuals with osteometric age ranges.

Preliminarily, several continuous parametric age-at-death models were considered. The best fitting was a two-mixture model of a half-gaussian (encompassing the high proportion of deaths expected soon after birth) and a truncated gaussian (encompassing deaths into normal adulthood). This model required four parameters: the SD of the half-gaussian, the mean and SD of the truncated gaussian, and the mixture proportion of the half-gaussian. Maximum Likelihood parameters were found using the JDEoptim function from the R package DEoptimR[49]. This was achieved by calculating the model PDF across the osteometric age range of each individual.

Phases were selected with n = 20 or more individuals, providing 9 phases to test. This was achieved by comparing the two summary statistics of interest (proportion of juvenile deaths in each phase, and mean age-at-death), with the same summary statistics when sampling n random individuals from the null distribution, where n is the total number of deaths in the test phase. This is conservative test, since the deaths in the test phase always contribute to the null distribution. Significance was calculated as follows.

To calculate summary statistics from the phase of interest:

1. Each individual was assigned a point age, by randomly sampling from the null across their osteometric age ranges.
2. Summary statistics were calculated.
3. Steps 1 and 2 were repeated 5000 times to give a distribution of each summary statistic.

To calculate summary statistics from the null distribution:

1. We sampled n individuals from the full dataset, where n is the number of individuals in the test phase.
2. Each individual was assigned a point age, by randomly sampling from the null across their osteometric age range.
3. Summary statistics were calculated.
4. Steps 1 to 3 were repeated 5000 times.

A one-tailed p-value for the mean age (lower than expected) was calculated by fitting a gaussian to the null distribution of mean ages, and calculating the proportion of the PDF below the observed mean age. This was averaged across all 5000 resamplings.

A one-tailed p-value for the proportion of juveniles (higher than expected) was calculated by fitting a binomial to the mean proportion of null juveniles, then calculating the proportion of

the PDF above the observed proportion of juveniles. This was averaged across all 5000 resamplings.

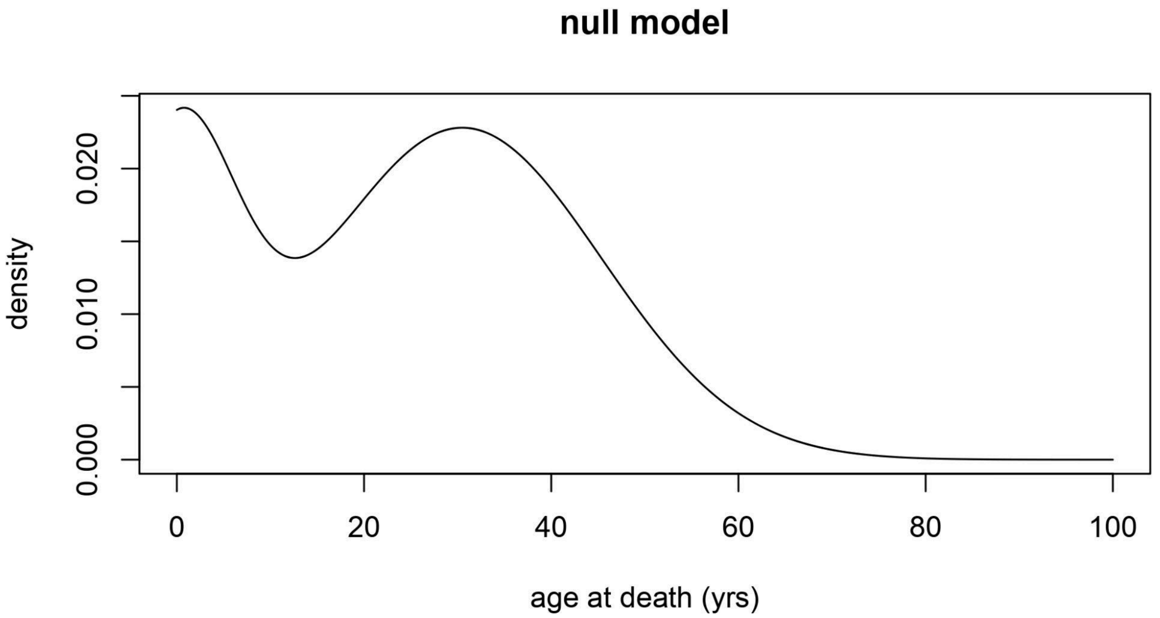

Figure S28. Continuous null model of age-at-death. Fitted to n=625 individuals with osteometric age ranges. Best fitting model was a two-mixture model of a half-gaussian (SD = 0.201) and a truncated Gaussian (mean = 30.492, SD =14.883, zero probability for age<=0), with the half-gaussian comprising 16.63% of the mixture.

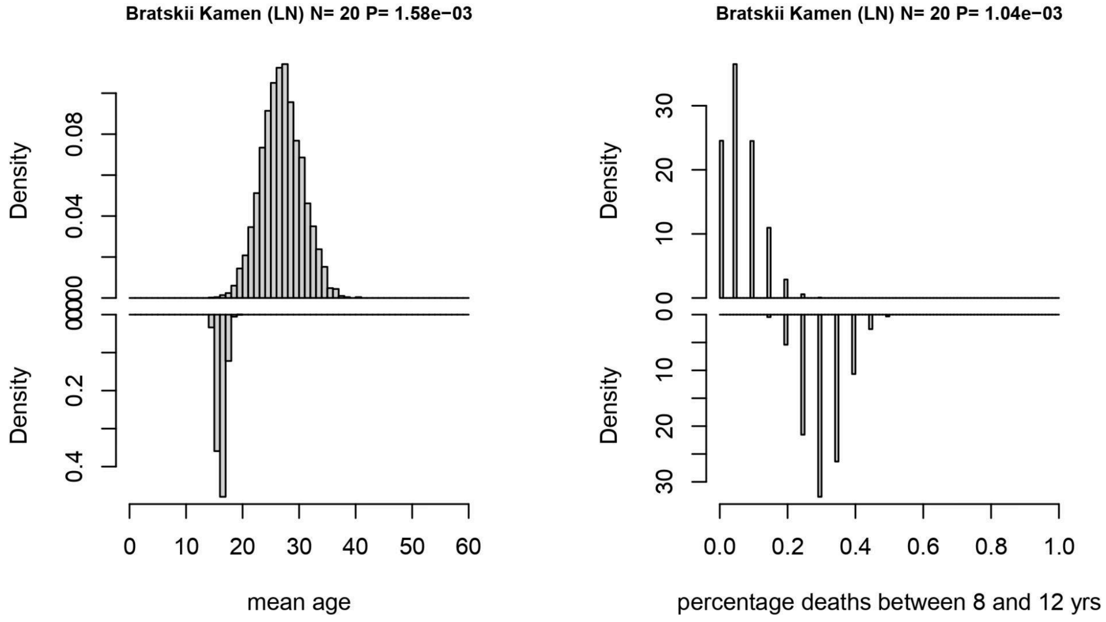

Figure S29. Age-at-death modelling for Bratskii Kamen (LN). Left: null distribution of mean age (top), test phase distribution of mean age (bottom). Right: null distribution of percentage of juvenile percentage (top), test phase distribution of juvenile percentage (bottom).

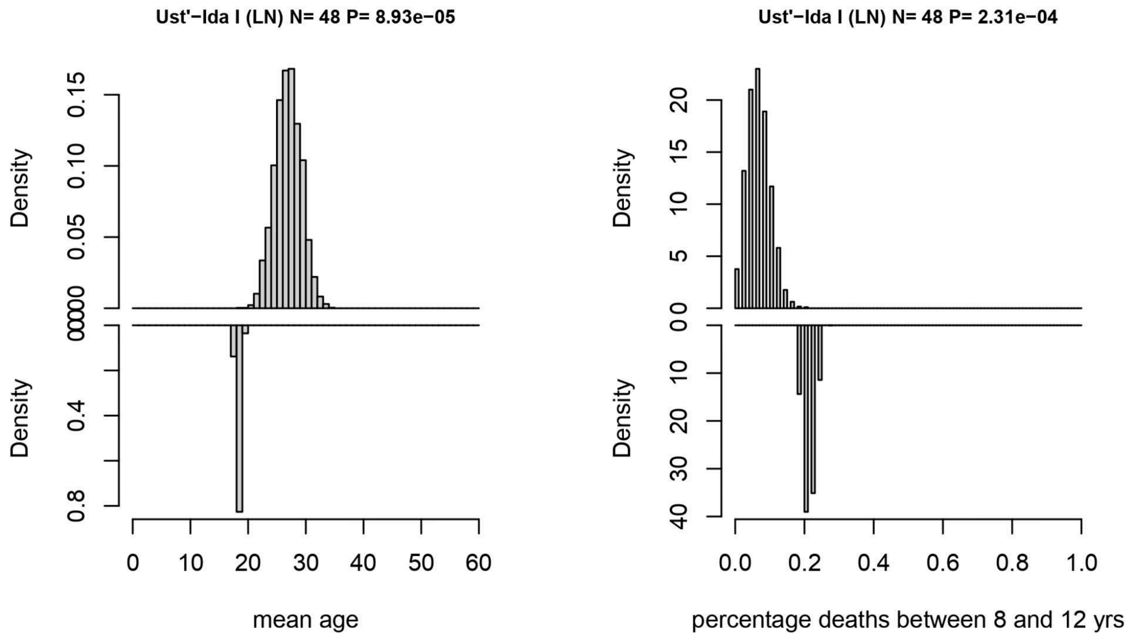

Figure S30. Age-at-death modelling for Ust'-Ida I (LN). Left: null distribution of mean age (top), test phase distribution of mean age (bottom). Right: null distribution of percentage of juvenile percentage (top), test phase distribution of juvenile percentage (bottom).

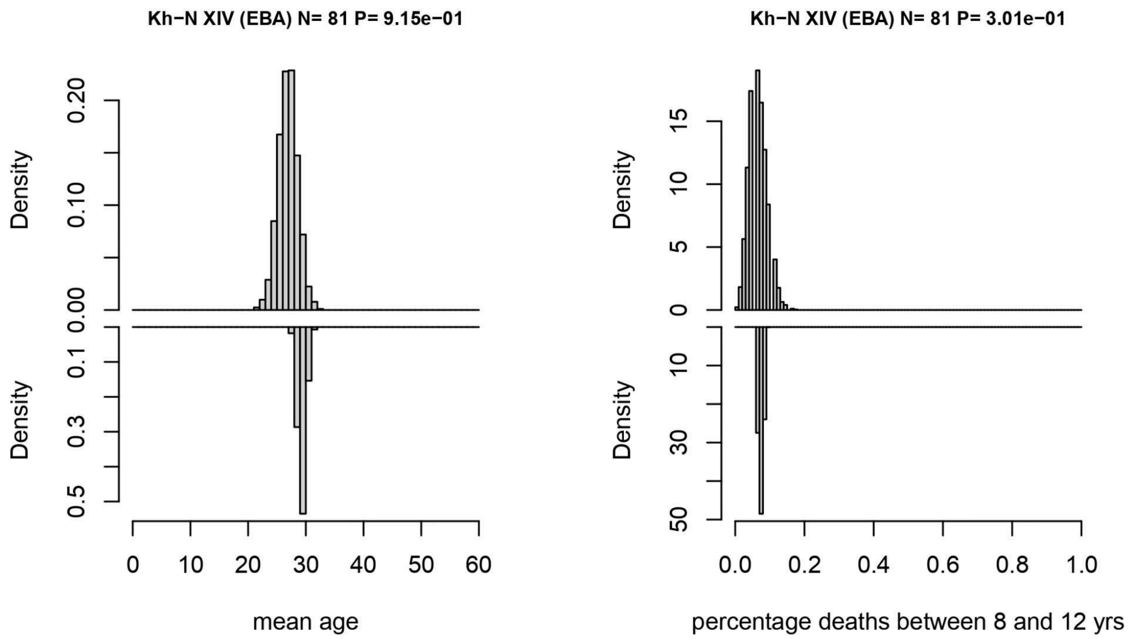

Figure S31. Age-at-death modelling for Kuzhir-Nuge XIV. Left: null distribution of mean age (top), test phase distribution of mean age (bottom). Right: null distribution of percentage of juvenile percentage (top), test phase distribution of juvenile percentage (bottom).

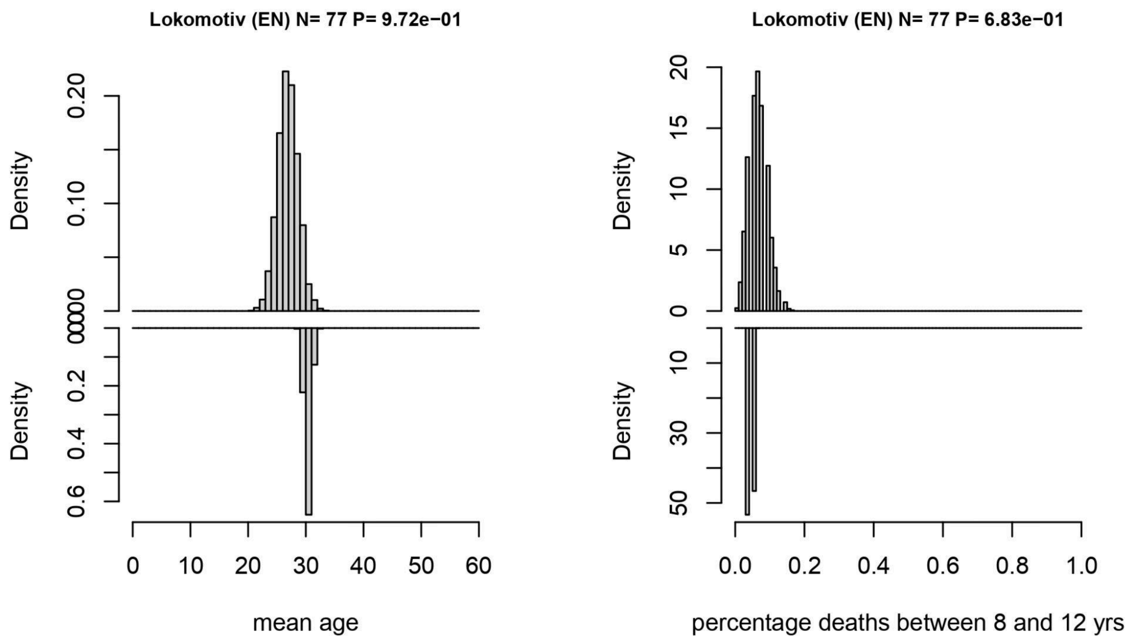

Figure S32. Age-at-death modelling for Lokomotiv. Left: null distribution of mean age (top), test phase distribution of mean age (bottom). Right: null distribution of percentage of juvenile percentage (top), test phase distribution of juvenile percentage (bottom).

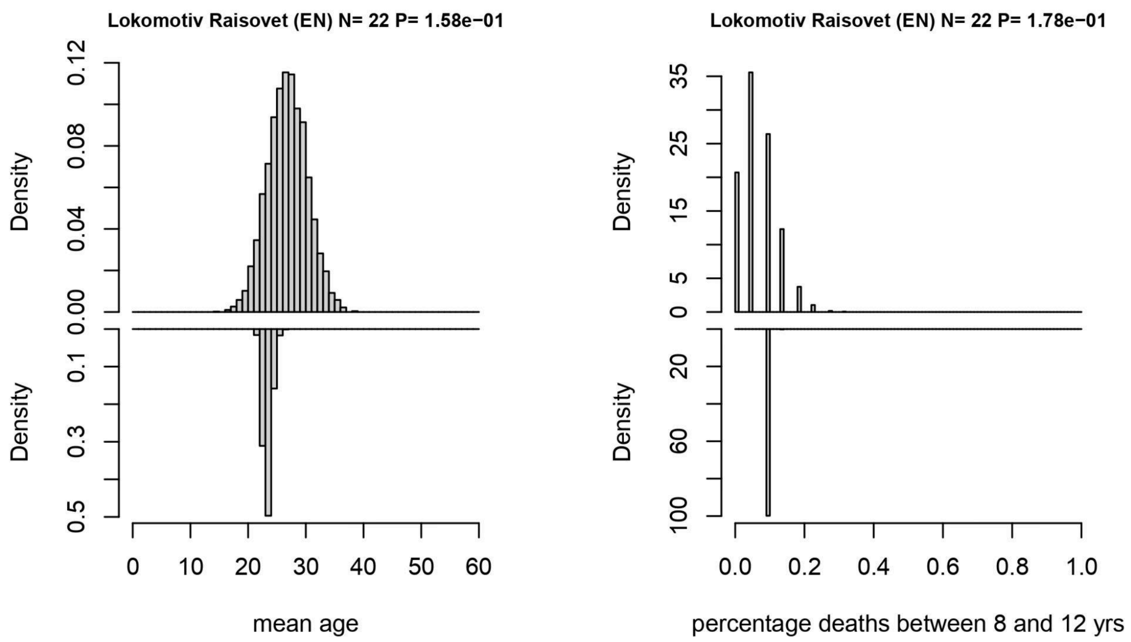

Figure S33. Age-at-death modelling for Lokomotiv Raisovet. Left: null distribution of mean age (top), test phase distribution of mean age (bottom). Right: null distribution of percentage of juvenile percentage (top), test phase distribution of juvenile percentage (bottom).

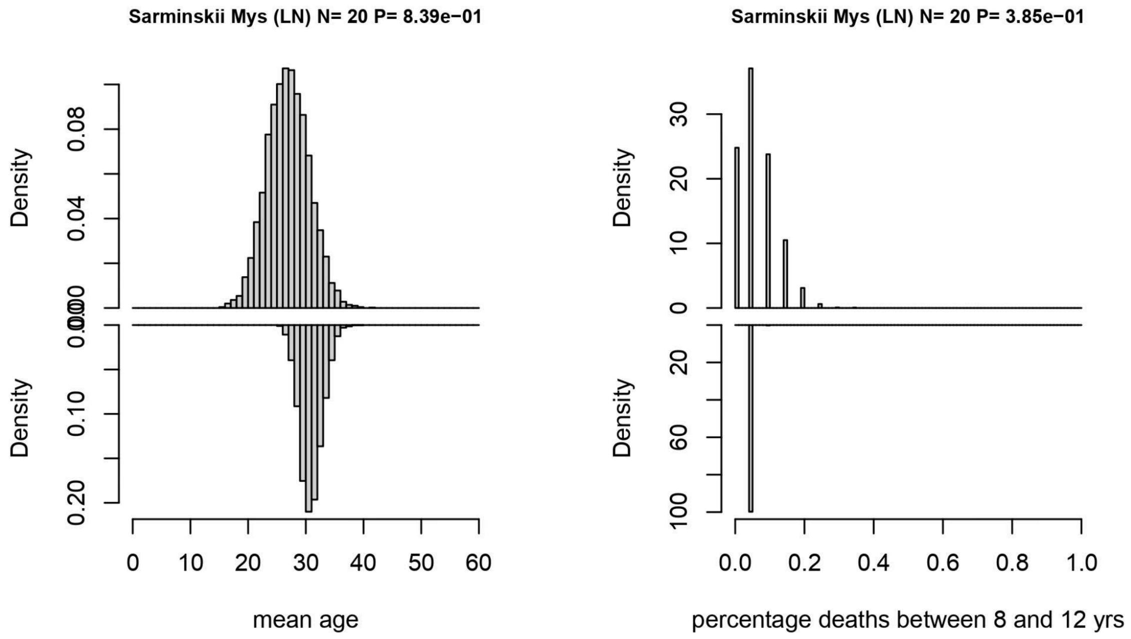

Figure S34. Age-at-death modelling for Sarminskii Mys. Left: null distribution of mean age (top), test phase distribution of mean age (bottom). Right: null distribution of percentage of juvenile percentage (top), test phase distribution of juvenile percentage (bottom).

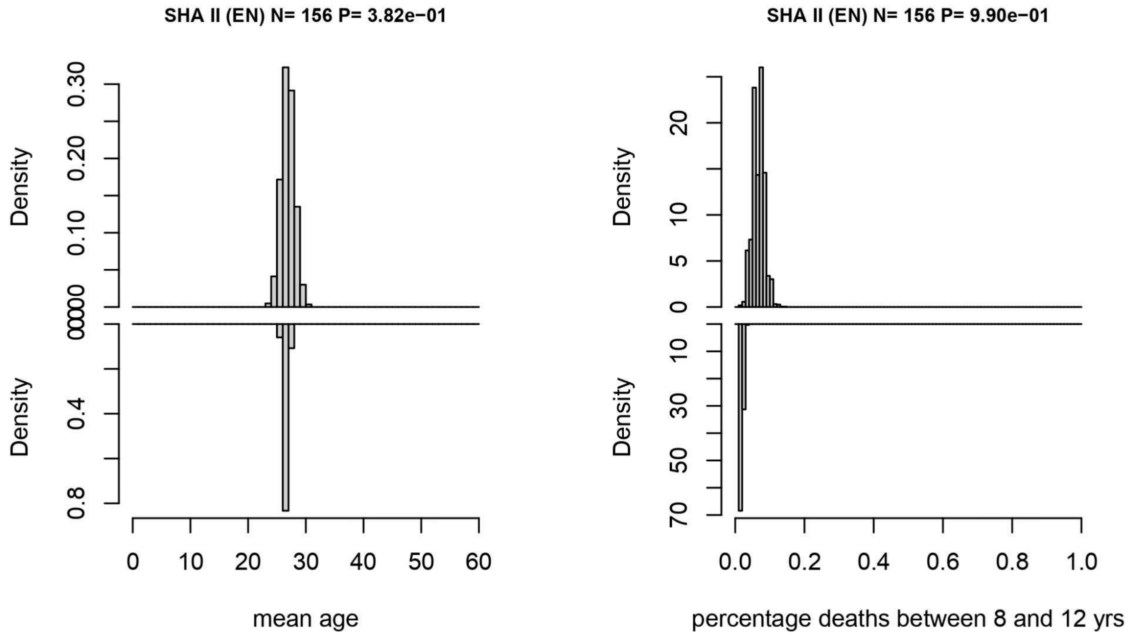

Figure S35. Age-at-death modelling for Shamanka II. Left: null distribution of mean age (top), test phase distribution of mean age (bottom). Right: null distribution of percentage of juvenile percentage (top), test phase distribution of juvenile percentage (bottom).

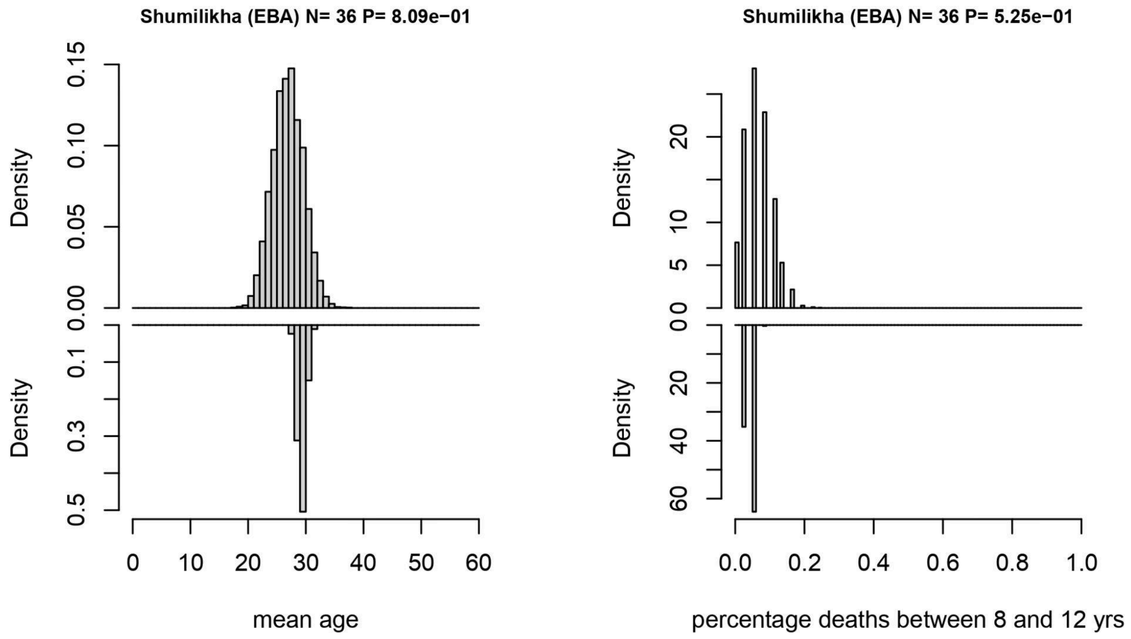

Figure S36. Age-at-death modelling for Shumilikha. Left: null distribution of mean age (top), test phase distribution of mean age (bottom). Right: null distribution of percentage of juvenile percentage (top), test phase distribution of juvenile percentage (bottom).

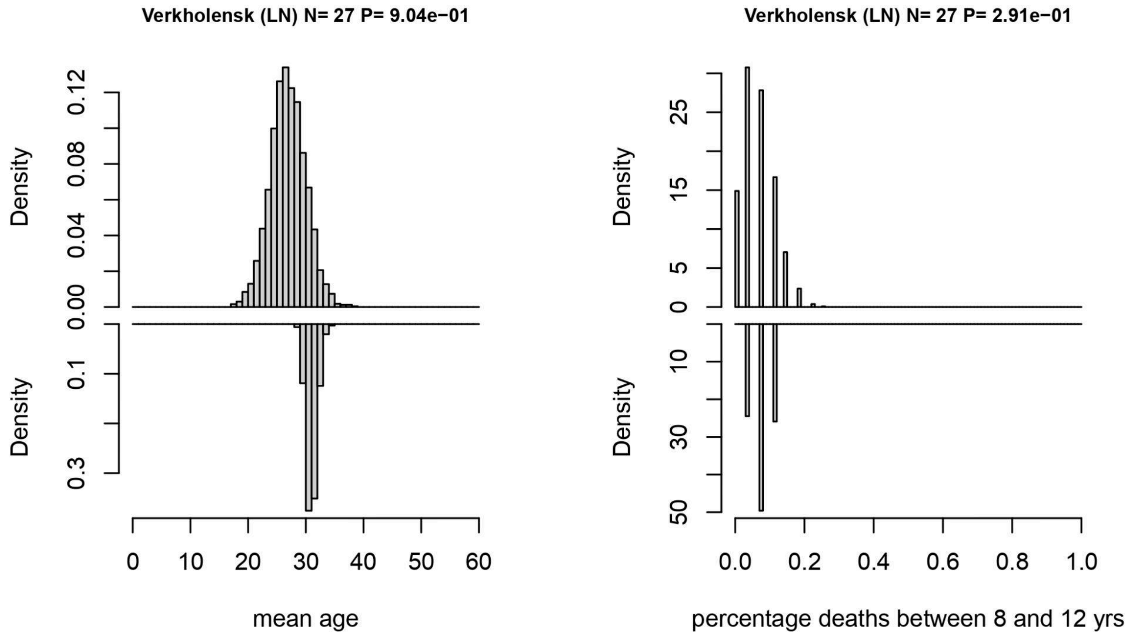

Figure S37. Age-at-death modelling for Verkholensk. Left: null distribution of mean age (top), test phase distribution of mean age (bottom). Right: null distribution of percentage of juvenile percentage (top), test phase distribution of juvenile percentage (bottom).

Supplementary Note 6: The effects of hg19 to hg38 liftover

In this study all newly generated genomes were mapped to the human genome reference build hg38, while the reference panel was mapped to build hg19 and lifted over to hg38. As hg38 provides improvements in complex genomic regions and since several regions were corrected, added, or removed for this build, it is possible that the use of a panel lifted over from hg19 could bias our results. In order to confirm that the effects of hg19 to hg38 liftover is negligible we investigated the impacts of liftover using both F statistics and various principle component analyses.

Firstly, to confirm that the PCA space is identical for the original panel mapped to hg37 and that same panel lifted over to hg38, we carried out smart PCA analyses for both panels. Each PCA plot was compared using a procrustes analysis as implemented in Vegan v2.6 (Extended Data Figure 2). From this test we found an almost complete alignment of points, with highly significant p-values for the two PCA plots. This finding strongly suggests that the overall structure of the data is preserved in PCA space even after lifting over to a new build.

Next, we investigated how mapping to each reference might affect our result using the test samples only. As depicted in Extended Data Figure 2, we found that points from the same sample were typically found closely together, suggesting minimal effects of mapping type on the placement of samples in PCA space. Furthermore, to confirm that this pattern is consistent in higher PCs as well, we plotted histograms of distances between points from the same sample in PC1 to PC10 (Figure S38). As depicted, this plot confirms the pattern observed in Extended Data Figure 2.

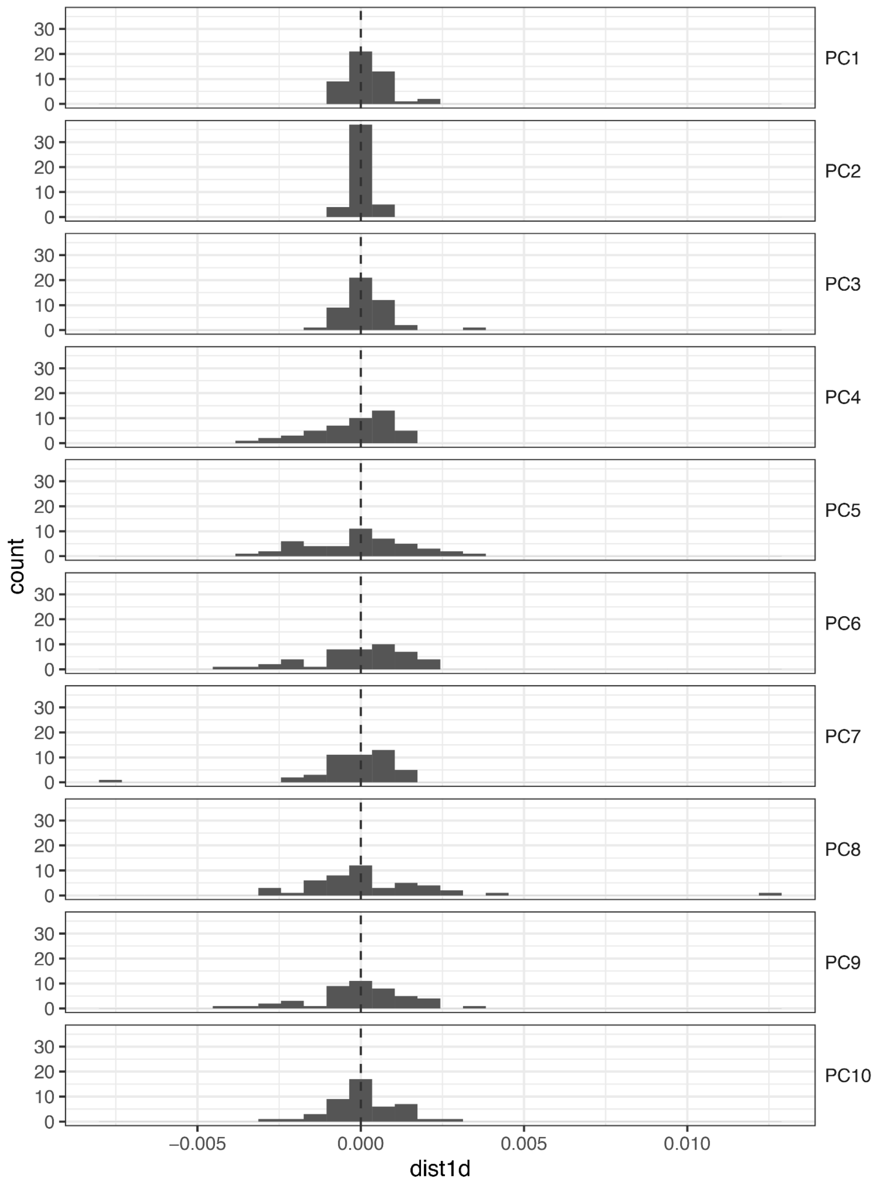

Figure S38. Distance between points mapped to hg19 and hg38, respectively. Histogram of the distances between points at PC1 to PC10.

Lastly, we investigated whether samples mapped to hg19 and lifted over to hg38 had a higher affinity to various populations from the reference panel, which were generated in the same way, than the test samples mapped directly to hg38. To test this, we carried out D-statistics using the following setup: *D(testSample (hg38), testSample (hg37->hg38 liftover); Pop3, Outgroup)*. In cases where the lifted over samples showed a higher affinity to the lifted over populations from the panel, we would expect a small shift to the left for all samples in the D-stats plot. However, as depicted in Figure S39, all values hovers around

zero, suggesting that liftover does not introduce a systematic bias in our data.

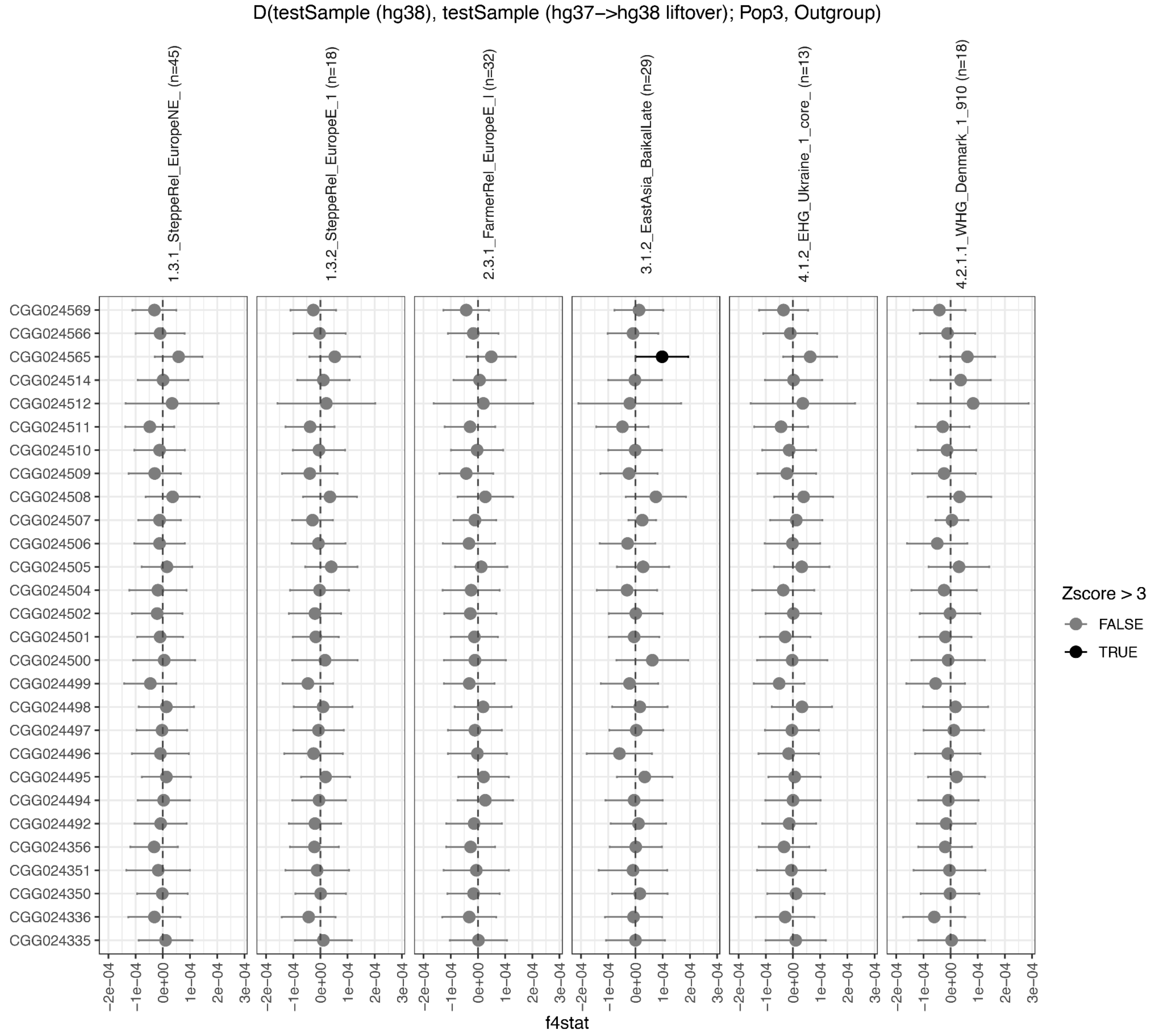

Figure S39. D-stats comparing the effect of mapping to hg19 and hg38. Sample-wise D-statistics.

References

1. Okladnikov, A. P. *Neolit I Bronzovyi Vek Pribaikaliya [Neolithic and Bronze Age of the Baikal Region]*. 411 (AS USSR Publications, Moscow, 1950).
2. Weber, A. W., Beukens, R. P., Bazaliiskii, V. I., Goriunova, O. I. & Savel'ev, N. A. Radiocarbon dates from Neolithic and Bronze Age hunter-gatherer cemeteries in the Cis-Baikal region of Siberia. *Radiocarbon* **48**, 127–166 (2006).
3. Weber, A. W. *et al.* Chronology of middle Holocene hunter–gatherers in the Cis-Baikal region of Siberia: Corrections based on examination of the freshwater reservoir effect. *Quaternary* **419**, 74–98 (2016).

4. Weber, A. W., Ramsey, C. B., Schulting, R. J., Bazaliiskii, V. I. & Goriunova, O. I. Middle Holocene hunter–gatherers of Cis-Baikal, Eastern Siberia: Chronology and dietary trends. *Archaeological Research in Asia* **25**, 100234 (2021).
5. Bazaliiskii, V. I. 3. Mesolithic and neolithic mortuary complexes in the Baikal region. in *Prehistoric Hunter-Gatherers of the Baikal Region, Siberia* 51–86 (University of Pennsylvania Press, Philadelphia, 2010).
6. Okladnikov, A. P. *Verkholenskii Mogil'nik—pamiatnik Drevnei Kul'tury Narodov Sibiri*. (Nauka, Novosibirsk, 1978).
7. Tiutrin, I. I. & Bazaliiskii, V. I. Mogil'nik v ust'e reki Idy v doline Angary. *Arkheologiia Paleoekologiia Ehnologiia Sib Dalnego Vostoka* **1**, 85–90 (1996).
8. Bronk Ramsey, C., Schulting, R. J., Bazaliiskii, V. I., Goriunova, O. I. & Weber, A. W. Spatio-temporal patterns of cemetery use among Middle Holocene hunter-gatherers of Cis-Baikal, Eastern Siberia. *Archaeological Research in Asia* **25**, 100253 (2021).
9. Waters-Rist, A., Bazaliiskii, V. I., Weber, A., Goriunova, O. I. & Katzenberg, M. A. Activity-induced dental modification in holocene siberian hunter-fisher-gatherers. *Am. J. Phys. Anthropol.* **143**, 266–278 (2010).
10. Naumova, O. Y. & Rychkov, S. Y. Siberian population of the New Stone Age: mtDNA haplotype diversity in the ancient population from the Ust'-Ida I burial ground, dated 4020-3210 BC by ^{14}C. *Anthropol. Anz.* **56**, 1–6 (1998).
11. Mooder, K. P., Schurr, T. G., Bamforth, F. J., Bazaliiski, V. I. & Savel'ev, N. A. Population affinities of Neolithic Siberians: a snapshot from prehistoric Lake Baikal. *Am. J. Phys. Anthropol.* **129**, 349–361 (2006).
12. Damgaard, P. *et al.* The first horse herders and the impact of early Bronze Age steppe expansions into Asia. *Science* **360**, (2018).
13. Svinin, V. V. Bronzovyi vek Priangar'ia: Mogil'nik Shumilikha. *Irkutskii gosudarstvennyi universitet, Irkutsk. [In Russian]* (1981).
14. Chard, C. S. NORTHEAST ASIA. *Asian Perspect.* **2**, 13–21 (1958).
15. Okladnikov, A. P. *Neoliticheskie pamiatniki nizhnei Angary (ot Serovo do Bratska)*

[Neolithic Sites of Lower Angara. (From Serovo to Bratsk)]. 327 (Nauka, Novosibirsk, 1976).

16. Lieverse, A. R. Human Osteological Evidence. in *Khuzhir-Nuge XIV, a Middle Holocene Hunter- Gatherer Cemetery on Lake* (eds Weber, A. W., Katzenberg, M. A. & Goriunova, O. I.) vol. 3 217–252 (EdmontonL The University of Alberta Press, Baikal, Siberia, 2007).
17. Lieverse, A. R. Human osteological evidence: health, activity, and demography. in *Kurma XI, a Middle Holocene Hunter-Gatherer Cemetery on Lake Baikal, Siberia: Archaeological and Osteological Materials* (eds Weber, A. W., Goriunova, O. I., McKenzie, H. G., Lieverse & A R) 173–202 (The University of Alberta Press, Edmonton, 2012).
18. Lieverse, A. R. *et al.* Demography, health, and activity of the Early Neolithic Shamanka II cemetery population. in *Shamanka II: An Early Neolithic Cemetery on the Southwest Shore of Lake Baikal, Siberia. Archaeological and Osteological Materials.* (eds Weber, A. W., Bazaliiskii, V. I. & Jessup, E.) 407–430 (Deutsches Archäologisches Institut, Berlin, 2024).
19. Roberts, C. A. *Infectious Disease: Introduction, Periostosis, Periostitis, Osteomyelitis, and Septic Arthritis*. 285–319 (Academic Press, London, 2019).
20. Seersholm, F. V. *et al.* Repeated plague infections across six generations of Neolithic Farmers. *Nature* **632**, 114–121 (2024).
21. Popli, D., Peyrégne, S. & Peter, B. M. KIN: a method to infer relatedness from low-coverage ancient DNA. *Genome Biol.* **24**, 10 (2023).
22. Allentoft, M. E. *et al.* Population genomics of post-glacial western Eurasia. *Nature* **625**, 301–311 (2024).
23. Keith, E. R., Podmore, R. G., Anderson, T. P. & Murdoch, D. R. Characteristics of Streptococcus pseudopneumoniae isolated from purulent sputum samples. *J. Clin. Microbiol.* **44**, 923–927 (2006).
24. Garriss, G. *et al.* Genomic Characterization of the Emerging Pathogen Streptococcus

pseudopneumoniae. *MBio* **10**, (2019).

25. Hull, N. C. & Schumaker, B. A. Comparisons of brucellosis between human and veterinary medicine. *Infect. Ecol. Epidemiol.* **8**, 1500846 (2018).
26. Tuon, F. F., Gondolfo, R. B. & Cerchiari, N. Human-to-human transmission of Brucella - a systematic review. *Trop. Med. Int. Health* **22**, 539–546 (2017).
27. Bendrey, R. *et al.* Approaching ancient disease from a One Health perspective: Interdisciplinary review for the investigation of zoonotic brucellosis. *Int. J. Osteoarchaeol.* **30**, 99–108 (2020).
28. D'Anastasio, R., Zipfel, B., Moggi-Cecchi, J., Stanyon, R. & Capasso, L. Possible brucellosis in an early hominin skeleton from sterkfontein, South Africa. *PLoS One* **4**, e6439 (2009).
29. Haeusler, M. Evolutionary origin of musculoskeletal problems. *Spinal evolution: Morphology, function, and pathology of the spine in hominoid evolution* 213–245 (2019).
30. L'Hôte, L. *et al.* An 8000 years old genome reveals the Neolithic origin of the zoonosis Brucella melitensis. *Nat. Commun.* **15**, 6132 (2024).
31. Long, G. S. *et al.* A 14th century CE Brucella melitensis genome and the recent expansion of the Western Mediterranean clade. *PLoS Pathog.* **19**, e1011538 (2023).
32. Kay, G. L., Sergeant, M. J., Giuffra, V. & Bandiera, P. Recovery of a medieval Brucella melitensis genome using shotgun metagenomics. MBio. 2014; 5: e01337–14. (2014).
33. Mutolo, M. J., Jenny, L. L., Buszek, A. R., Fenton, T. W. & Foran, D. R. Osteological and molecular identification of Brucellosis in ancient Butrint, Albania. *Am. J. Phys. Anthropol.* **147**, 254–263 (2012).
34. Sikora, M. *et al.* The spatiotemporal distribution of human pathogens in ancient Eurasia. *Nature* **643**, 1011–1019 (2025).
35. Wanke, M. M. Canine brucellosis. *Anim. Reprod. Sci.* **82-83**, 195–207 (2004).
36. Ye, Y.-B. *et al.* A specific reverse complement sequence for distinguishing Brucella canis from other Brucella species. *Front. Vet. Sci.* **9**, 983482 (2022).
37. Minogue, T. D. *et al.* Whole-genome sequences of 24 Brucella strains. *Genome*

Announc. **2**, (2014).

38. Santos, R. L., Souza, T. D., Mol, J. P. S., Eckstein, C. & Paíxão, T. A. Canine brucellosis: An update. *Front. Vet. Sci.* **8**, 594291 (2021).
39. Losey, R. J. *et al.* Burying dogs in ancient Cis-Baikal, Siberia: temporal trends and relationships with human diet and subsistence practices. *PLoS One* **8**, e63740 (2013).
40. Losey, R. J. *et al.* Canids as persons: Early Neolithic dog and wolf burials, Cis-Baikal, Siberia. *Journal of Anthropological Archaeology* **30**, 174–189 (2011).
41. Brock, F., Higham, T., Ditchfield, P. & Ramsey, C. B. Current Pretreatment Methods for AMS Radiocarbon Dating at the Oxford Radiocarbon Accelerator Unit (Orau. vol. Radiocarbon52 103–112 Preprint at (2010).
42. DeNiro, M. J. Post-mortem preservation and alteration of in vivo bone collagen isotope ratios in relation to paleodietary reconstruction. *Nature* **317**, 806–809 (1985).
43. van Klinken, G. J. Bone Collagen Quality Indicators for Palaeodietary and Radiocarbon Measurements. *J. Archaeol. Sci.* **26**, 687–695 (1999).
44. Bronk Ramsey, C. Bayesian analysis of radiocarbon dates. *Radiocarbon* **51**, 337–360 (2009).
45. Reimer, P., Austin, W. & Bard, E. The IntCal20 Northern Hemisphere Radiocarbon Age Calibration Curve (0–55 cal kBP. *Radiocarbon* **62**, 725–757 (2020).
46. Ramsey, C. B. Methods for Summarizing Radiocarbon Datasets. *Radiocarbon* **59**, 1809–1833 (2017).
47. Weber, A. W., Schulting, R. J., Ramsey, C. B. & Bazaliiskii, V. I. Biogeochemical data from the Shamanka II Early Neolithic cemetery on southwest Baikal: Chronological and dietary patterns. *Quat. Int.* **405, Part B**, 233–254 (2016).
48. Schulting, R. J., Ramsey, C. B., Bazaliiskii, V. I., Goriunova, O. I. & Weber, A. Freshwater Reservoir Offsets Investigated Through Paired Human-Faunal 14C Dating and Stable Carbon and Nitrogen Isotope Analysis at Lake Baikal, Siberia. *Radiocarbon* **56**, 991–1008 (2014).
49. Conceicao, E. L. T. Differential Evolution Optimization in Pure R [R package DEoptimR

version 1.1-3]. (2023).
